# Supplementary material for: RBL2-E2F-GCN5 guide cell fate decisions during tissue specification by regulating cell-cycle-dependent fluctuations of non-cell-autonomous signaling
Source: Cell Rep. 2023 Sep 19;42(9):113146. doi: 10.1016/j.celrep.2023.113146 (PMC12823551; doi:10.1016/j.celrep.2023.113146)
Supplement: Document S1. Figures S1–S7 and Table S3 [file mmc1.pdf]

Cell Reports, Volume *42*

Supplemental information

RBL2-E2F-GCN5 guide cell fate decisions during tissue specification by regulating cell-cycle-dependent fluctuations of non-cell-autonomous signaling

Stefania Militi, Reshma Nibhani, Morteza Jalali, and Siim Pauklin

SUPPLEMENTARY INFORMATION

Supplementary Table 3. Antibodies, primers and chemical compounds.

Antibody name	Techniques	Catalogue name	Company
Goat anti-human Nanog	IF	AF1997	R&D Systems
Mouse anti-human Oct4	IF	sc-5279	Santa Cruz Biotechnology
Goat anti-human SOX2	IF	AF2018	R&D Systems
Rabbit anti-human Eomes	IF	ab23345	Abcam
Goat anti-human Brachyury	IF	AF2085	R&D Systems
Goat anti-human SOX17	IF	AF1924	R&D Systems
PAX6 rabbit polyclonal	IF, WB	PRB-278P-100	Cambridge BioScience
pRb mouse monoclonal	IF, WB	554136 (G3-245)	BD Pharmingen
RBL1/p107 (C-18) rabbit polyclonal	IF, WB	sc-318	Santa Cruz Biotechnology
RBL2/p130 (C-20) rabbit polyclonal	IF, WB, CHIP	sc-317	Santa Cruz Biotechnology
SOX1 goat polyclonal	IF	AF3369	R&D Systems
Actin mouse monoclonal	WB	MAB1501	Chemicon
E2F1 (C-20)	WB, CHIP	sc-193	Santa Cruz Biotechnology
E2F4 (A-20)	WB, CHIP	sc-1082x	Santa Cruz Biotechnology
SOX1 goat polyclonal	IF, WB	AF3369	R&D Systems
p75 (C-20) goat polyclonal	WB	sc-6188	Santa Cruz Biotechnology
WNT4 (m-70) rabbit polyclonal	WB	sc-13962	Santa Cruz Biotechnology
WNT5A (H-58) rabbit polyclonal	WB	sc 30224	Santa Cruz Biotechnology
WNT8A rabbit polyclonal	WB	SAB1411397	Sigma
HES5 rabbit polyclonal	WB	ab25374	Abcam
DLL1 H-265 rabbit polyclonal	WB	sc-9102	Santa Cruz Biotechnology
DLL3 H-110 rabbit polyclonal	WB	sc-67270	Santa Cruz Biotechnology
P-ser33-B-cat rabbit polyclonal	WB	sc-16743-R	Santa Cruz Biotechnology
B-catenin (H-102) rabbit polyclonal	WB, CHIP	sc-7199	Santa Cruz Biotechnology
Beta Tubulin 3/ Tuj1 [GT1338] mouse monoclonal	IF	GTX631831-GTX	Stratech
CD133-BV786, Mouse Anti-Human, clone W6B3C1	FACS	BD 747640	BD Biosciences
Mouse IgG1-BV786l	FACS, isotype control for CD133	BD 563330	BD Biosciences
Alexa647 Mouse anti-SSEA-4 clone	FACS	BD 560219	BD Biosciences
Alexa647 Mouse IgG3	FACS, isotype control for SSEA4	BD 560803	BD Biosciences

Primer name	Techniques	
pRB	qPCR	F ccaggccccctacctttgtcacc<br>R ttgttggtgttggcagaccttct
RBL1/p107	qPCR	F ccaagaaagcgctctgctgtacaa<br>R acagacgcgtttggcagggg
RBL2/p130	qPCR	F cccctctgatggagggacgcc<br>R ttggctgtgacagtggcggt
PBGD	qPCR	F ggagccatgtctggtaacgg<br>R ccacgcgaatcactctcatct
Nanog	qPCR	F catgagtgtggatccagcttg<br>R cctgaataagcagatccatgg
OCT4	qPCR	F agtgagaggcaacctggaga<br>R acactcggaccacatccttc
SOX2	qPCR	F tggacagttacgcgcacat<br>R cgagtaggacatgctgtaggt
Eomes	qPCR	F atcattacgaaacagggcaggc<br>R cggggttggtatttgtgtgtaagg
Gsc	qPCR	F gaggagaaagtggaggtctggtt<br>R ctctgatgaggaccgcttctg
SOX17	qPCR	F cgcacggaatttgaacagta<br>R ggatcagggacctgtcacac
Brachyury T	qPCR	F tgcttccctgagacccagtt<br>R gatcacttctttcctttgcatcaag
Mesp1	qPCR	F gaagtggttccttggcagac<br>R tcctgcttgcctcaaagtgt
SOX1	qPCR	Sigma Quantitect primers
PAX6	qPCR	F ctttgcttgggaaatccgag<br>R agccaggttgcgaagaactc
SOX9	qPCR	F aggaagtcggtgaagaacggg<br>R ctctcgcttcaggtcagcct
SOX10	qPCR	F atccaggcccactacaagagc<br>R actggtccaactcagccacat
P75	qPCR	F acaagacctcatagccagcac<br>R ctgttggctccttgcttgttc
HNK1	qPCR	F cgacgacgacaacacctaca<br>R cctggtagcctcccttcaca
Olig3	qPCR	F agccgtctcaactcggtct<br>R catggctaggttcaggtcgtg
Otx2	qPCR	F atcccttgtccacctcctct<br>R gctggtgatgcataggggtc
HES5	qPCR	Sigma Quantitect primers
Delta1	qPCR	F acctcgcaacagaaaaccca<br>R gtgttcgtcacacacgaagc
Delta3	qPCR	F cggatgcactcaacaacct<br>R gaagatggcaggtagctcaa
WNT4	qPCR	F cgtcttcggcaaggtggtga<br>R ctgaccccatgcactgtcct
WNT5A	qPCR	F tgtgccacttgtatcaggacc<br>R tgcctatctgcatcaccctg
WNT8A	qPCR	F ctgtggctgtgatgggtcaa<br>R ttcatcagggctctggcatc
WNT3	qPCR	F ttgttccaactattgggggc<br>R gctgtgagcccagagatgtg
SFRP1	qPCR	F gtcatgcagttcttcggcttc<br>R ctcgttgtcacagggaggac
FRAT2	qPCR	F gtgctctcgggaaacctcat<br>R caaggagcctgagggctg
FOXD3	qPCR	F actgcttcgtcaagatcccc<br>R ctgtaagcgccgaagctct

KAL1	qPCR	F ttcaaagacgacgacccact<br>R ttttcgtgggtcatgccaga
TFAP2A	qPCR	F ccaagtccaacagcaatgcc<br>R gttgagacactcgggtggtg
TFAP2C	qPCR	F gccgtaatgaaccccactga<br>R accggcctccatttttcgat
OLIG3	qPCR	F atgcacgacctgaacctagc<br>R ccccatagatctcgccaacc
ROR1	qPCR	F cccagaagctgcgaactgta<br>R gtgtgtgtggggatactggg
CXCL12	qPCR	F cgtcagcctgagctacagat<br>R tagcttcgggtcaatgcaca
ZIC3	qPCR	F gcaaagtgtgcgacaagtcc<br>R tgcacagtaggttcggcatt
ASCL1	qPCR	F ctcaacttcagcggctttgg<br>R cgcagtgtctccaccttact
NEUROD1	qPCR	F cgctttgcaagggcttatcc<br>R aggcgactggtaggagtagg
NEUROG1	qPCR	F aatatctcccgggcgtctga<br>R gttcaagttgtgcatgcggt
FGF8	qPCR	F gctgcagaatgccaagtacg<br>R ggcgggtagttgaggaactc
SOX3	qPCR	F gataagcctacccttcccgc<br>R gtgtccctacggggttcttg
FOXD1	qPCR	F tctgtgagttcatcagcggc<br>R gcaggaagctgccgttgtc
ID1	qPCR	F ccagcacgtcatcgactaca<br>R acacaagatgcgatcgtccg
RORB	qPCR	F acgtcattgacctgcccaag<br>R cctggtgctaactgcccatt
PAX6	ChIP-qPCR	F ttcgggtctctccgatgaag<br>R gaaactagtcttgccgagtgc
SOX1	ChIP-qPCR	F gctagaagttgcagcctccga<br>R caagttgcagctccgcttttg
SOX9	ChIP-qPCR	F ggcattccgagagtacgaca<br>R gcagctgattggacccgatt
SOX10	ChIP-qPCR	F cctgtgttgctgcatcccc<br>R agggagtaggcgcttaagga
Olig3	ChIP-qPCR	F ctccgggtgagtcaaaccac<br>R gactcccgttttagctgactt
P75	ChIP-qPCR	F gagagtgaaccctgtggcg<br>R ttagagcctctcacccatccc
HoxD1	ChIP-qPCR	F tcaagggaaagacgtgagcc<br>R ttaccggggacgggtgagat
HoxA2	ChIP-qPCR	F acagaacttatgtggctggga<br>R gcattgtttgggactgtcgg
HES5	ChIP-qPCR	F gaagaagggtggggtccttg<br>R aagctagtgaaaggcctggg
DLL1	ChIP-qPCR	F cgcttgcatttcctccttgc<br>R ttttcagtcccgtgtttccg
DLL3	ChIP-qPCR	F gtttgggagggtggctttgt<br>R gtgcgctggtttgtgtgaat
WNT4	ChIP-qPCR	F gccaagagagcttccctaaact<br>R atccgaaacctcgcttctgg
WNT3	ChIP-qPCR	F tgaacccctcaaggaggaga<br>R acggagccgagtgtcatttg
WNT8A	ChIP-qPCR	F ctgggtggccctaaggttg<br>R ccatctctgcaacagtccct
SFRP1	ChIP-qPCR	F gaggccttggagaggaacat<br>R cagacatcacgcctacgcaa

Otx2	ChIP-qPCR	F cctttaccctttctggccgt<br>R gcgggttagggagtgactg
DLL1 F	Luciferase assay	TCTTACGCGTGAGGCGTAGTTACTTGGCTTTGCCTTAGAGCGGA
DLL1 R	Luciferase assay	TCTCGAGCTAGTGTATCTATGGGTTCCCCCTCACCATTTTCTTGTTT TTTCCTTGC
DLL3 F	Luciferase assay	TCTTACGCGTGATGTGAAGACGGAATTTCCTGCCCATTTGCTCCTC
DLL3 R	Luciferase assay	TCTCGAGCTAGTGAGAGAATGGCCCCGCCCCTTCAGGC
WNT4 F	Luciferase assay	TCTTACGCGTGCTAGTATGGGAGGATAAATCGAACAAGCACATAGAA AACCAAATGCAAA
WNT4 R	Luciferase assay	TCTCGAGCTAGCGGCGGCGGAGGCGGGCG
WNT8A F	Luciferase assay	TCTTACGCGTGTAAATAAATAAATAAATAAATAAATAAATAAAATAAAATAAAAGACAGATTCCCACCTAAGGAGCTGAAGTCCTAGTAA
WNT8A R	Luciferase assay	TCTCGAGCTAGATTTCTTTCTTCTATATGTCAATTTTGTCTCTTCCTGCTTCTTTGGCCC
**Source Name**	**[Working] uM**	**Class/Target**
(+)-JQ1	1	Bromodomains - BRD2, BRD3, BRD4, BRDT (BET)
(-)-JQ1 (inactive)	1	Bromodomains - Negative control
PFI-1	5	Bromodomains - BRD2, BRD3, BRD4, BRDT (BET)
I-BET	1	Bromodomains - BRD2/3/4
Bromosporine	1	Bromodomains - pan-Bromodomain
CBP/BRD4 (0383)	5	Bromodomains - CBP, BRD4(1)
SGC-CBP30	1	Bromodomains - CREBBP, EP300
I-CBP112	1	Bromodomains - CREBBP, EP300
RVX-208	5	Bromodomains - BRD2, BRD3, BRD4, BRDT (BET, BD2)
SMARCA	2.5	Bromodomains - SMARCA, PB1
PB1/SMARCA	1	Bromodomains - SMARCA, PB1
PFI-3	1	Bromodomains - SMARCA2/4, PB1(5)
GSK2801	1	Bromodomains - BAZ2A, BAZ2B
PFI-4	1	Bromodomains - BRPF1B
TRIM24/BRPF	10	Bromodomains - TRIM24/BRPF
OF-1	5	Bromodomains - pan-BRPF
Belinostat	5	HDAC - hydroxamic acids
CXD101	1	HDAC
Valproic acid	1000	HDAC - aliphatic acid compounds
Entinostat	0.5	HDAC - ortho-amino anilides
SAHA	2.5	HDAC - hydroxamic acids
Trichostatin A	0.5	HDAC - hydroxamic acids - Class I & II
SRT1720	1	HDAC - SIRT1 activator
EX 527	1	HDAC - SIRT1
CI-994	1	HDAC - 1,2,3,(8)

CPI-360	10	Histone methyltransferase - EZH2 and EZH1
CPI-413	10	Histone methyltransferase - EZH2 and EZH1
UNC0638	1	Histone methyltransferase - G9a, GLP
UNC0642	1	Histone methyltransferase - G9a, GLP
A-366	2	Histone methyltransferase - G9a, GLP
Chaetocin	0.05	Histone methyltransferase - SUV39H1
PFI-2	2	Histone methyltransferase - SETD7
SGC0946	7.5	Histone methyltransferase - DOT1L
GSK343	3	Histone methyltransferase - EZH2
UNC1999	1	Histone methyltransferase - EZH2
LLY-507	1	Histone methyltransferase - SMYD2
Tranylcypromine	20	Lysine demethylases - LSD1
GSK-LSD1 (irreversible)	0.5	Lysine demethylases - LSD1
GSK690	5	Lysine demethylases - LSD1
GSK J4	10	Lysine demethylases - JMJD3, UTX, JARID1B
GSK J5 (inactive)	10	Lysine demethylases - Negative control
IOX1 (5-carboxy-8HQ)	40	Lysine demethylases - pan-2-OG
Methylstat (Ester)	2.5	Histone demethylase
(E)-JIB-04	0.05	Histone demethylase - Pan JmjC
ML324	5	Histone demethylase - JMJD2E
IOX2	10	Prolyl-Hydroxylases - PHD2 (EGLN1)
OICR-9429	1	Methyl Lysine Binder - WDR5
UNC1215	5	Methyl Lysine Binder - L3MBTL3
5-Azacitidine	10	DNA methyltransferase (DNMT)
5-Azadeoxycitidine	5	DNA methyltransferase (DNMT) - DNMT1/3
Olaparib	1	Poly ADP ribose polymerase (PARP)
Rucaparib	10	Poly ADP ribose polymerase (PARP)
K00135	1	Kinase inhibitor - ATP competitive - PIM
5-Iodotubercidin	1	Kinase inhibitor - ATP mimetic - Haspin
C646	1	Histone acetyltransferase (HAT) - p300/CBP
DUAL1946	1	
GSK484	1	Peptidyl arginine deiminase (PAD4)
KDOBA67	10	Histone demethylase
BAZ2-ICR	1	Bromodomains - BAZ2A, BAZ2B
NI-57	1	Bromodomains - pan-BRPF
LP99	1	Bromodomains - BRD9, BRD7
SGC707	1	Arginine methyltransferase - PRMT3
RGFP966	10	HDAC - HDAC3
PCI-34051	5	HDAC - HDAC8

Rocilinostat	10	HDAC - HDAC6
Tubastatin A HCl	10	HDAC - HDAC6
KDOAM-25a	1	Lysine demethylases - JARID
KDM5-C70	10	Histone demethylase - JARID1
MAZ1805	1	t-RNA sythetase
MAZ1392	1	t-RNA sythetase
BI-9564	1	Bromodomains - BRD9, BRD7
NVS-CECR2-1	1	Bromodomains - CECR2
GSK106	1	Peptidyl arginine deiminase (PAD4)
J556-42R	1	Arginine methyltransferase - PRMT5
J556-63R	1	Arginine methyltransferase - PRMT5
J556-70R	1	Arginine methyltransferase - PRMT5
A-196	1	Histone methyltransferase - SUV420H1/H2
BAY-598	1	Histone methyltransferase - SMYD2
J556-143	1	Arginine methyltransferase - PRMT5
MS049	1	Arginine methyltransferase
MS023	1	Arginine methyltransferase - Type I PRMTs
MS003	1	Arginine methyltransferase - negative control
SGI-1776	10	Kinase inhibitor - Haspin
CHR-6494	1	Kinase inhibitor - Haspin
CPI-169	10	Histone methyltransferase - EZH2, EZH1
UNC2400	1	Histone methyltransferase - EZH2
GSK864	5	Dehydrogenase
GSK8814	10	Bromodomains - ATAD2
GSK8815	10	Bromodomains - ATAD2
GSK959	1	Bromodomains - BRPF1
NVS-CECR2-C	1	Bromodomains - CECR2
BAY-299	1	Bromodomains - BRD1, TAF1
PCI-24781	10	HDAC
Romidepsin	1	HDAC
Mocetinostat	10	HDAC
Santacruzamate	50	HDAC
KDOAM32	1	Lysine demethylases - JARID
MS409N	1	Arginine methyltransferase - PRMT4, PRMT6 inactive control
TP-064	1	Arginine methyltransferase - PRMT4
TP-064N	1	Arginine methyltransferase - PRMT4
A-395	1	Methyl Lysine Binder - EED
A-395N	1	Methyl Lysine Binder - EED
I-BRD9	10	Bromodomains - BRD9

TP-472	1	Bromodomains - BRD9
TP-472N	1	Bromodomains - BRD9
KDOPZ-32a	1	Lysine demethylases - KDM5
KDOOA012000	1	Lysine demethylases - KDM2
AMI-1	50	Arginine methyltransferase - PRMT
TMP269	10	HDAC - 4, 5, 7, 9
AGK2	10	HDAC - SIRT2
GSK6853	1	Bromodomains - BRPF1/2/3
GSK9311	1	Bromodomains - BRPF1/2/3
LLY-283	1	Arginine methyltransferase - PRMT5
TD001851a	1	Methyl Lysine Binder/tudor domain - Spin1
TDOSI000058a	1	Methyl Lysine Binder/tudor domain - Spin1
TD001863a	1	Methyl Lysine Binder/tudor domain - Spin1
TDOSI000062a	1	Methyl Lysine Binder/tudor domain - Spin1
TD001857a	1	Methyl Lysine Binder/tudor domain - Spin1
TD001856a	1	Methyl Lysine Binder/tudor domain - Spin1
TD001858a	1	Methyl Lysine Binder/tudor domain - Spin1
TMP195	1	HDAC - 4,5,7,9
GSK2879552	10	Lysine demethylases - LSD1
TDO20821a	1	Methyl Lysine Binder/tudor domain - Spin1
TDO20824a	1	Methyl Lysine Binder/tudor domain - Spin1
TDO20823a	1	Methyl Lysine Binder/tudor domain - Spin1
A-485	1	Histone acetyltransferase (HAT) p300/CBP
A-486	1	Histone acetyltransferase (HAT) p300/CBP
GSK4027	1	Bromodomains - PCAF, GCN5
GSK4028	1	Bromodomains - PCAF, GCN5
L-Moses	1	Bromodomains - PCAF, GCN5
D-Moses	1	Bromodomains - PCAF, GCN5
PFI-5	1	Histone methyltransferase - SMYD2
YX39-31b	1	Methyl Lysine Binder/tudor domain - Spin1
TDO208229	1	Methyl Lysine Binder/tudor domain - Spin1
TDO01856a	1	Methyl Lysine Binder/tudor domain - Spin1
TDO20826a	1	Methyl Lysine Binder/tudor domain - Spin1
Bortezomib	0.1	Protesome
Carfilzomib	0.1	Protesome
RTS-V5	1	Protesome and HDAC
dBRD9	1	Bromodomains - BRD9
BI-7273	0.1	Bromodomains - BRD9/7
CPI-621	0.1	Lysine demethylases - KDM5

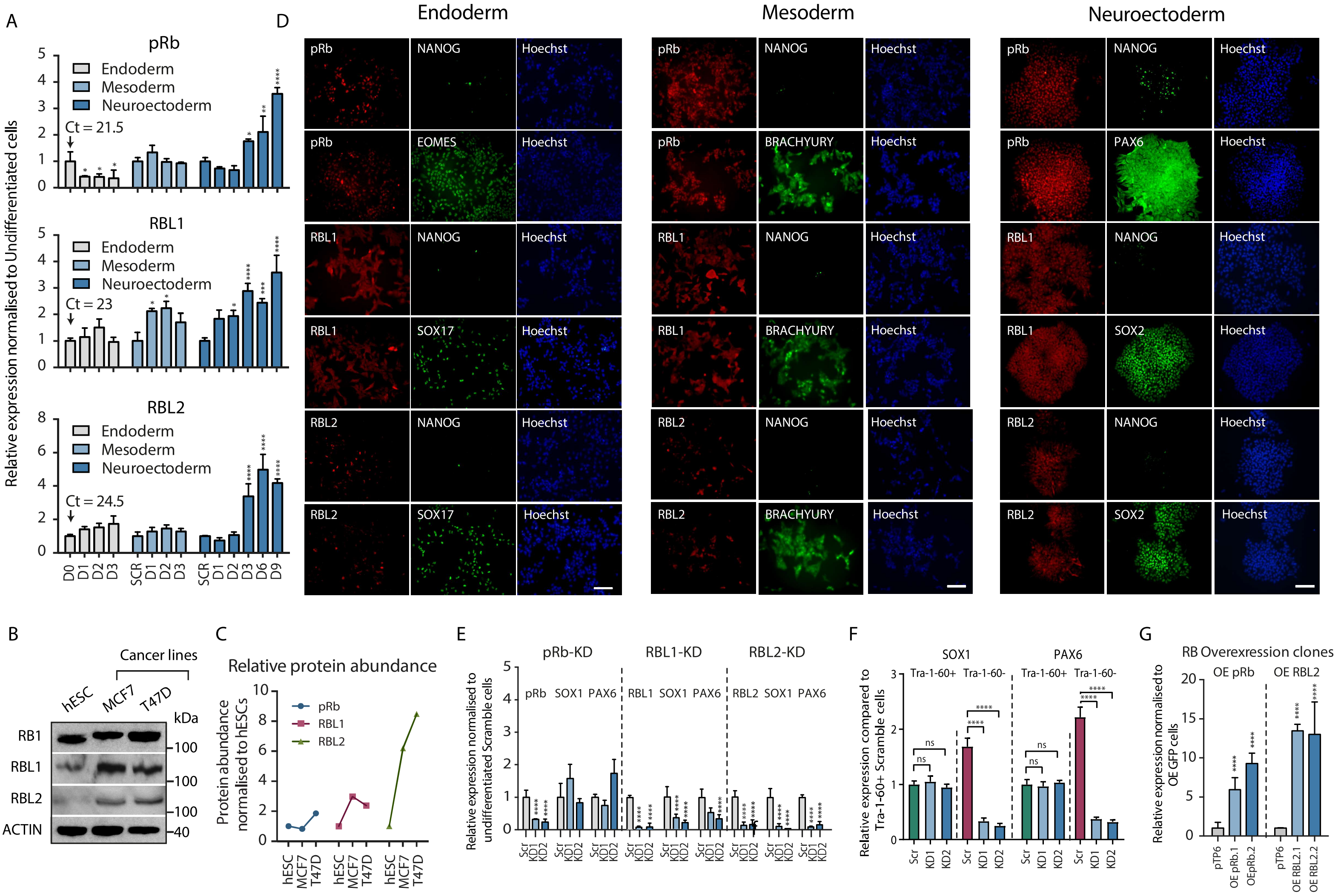

Figure S1: Functional studies of Retinoblastoma-family proteins in hPSCs, Related to Figure 1. (A) Rbl2 is upregulated during specification of human pluripotent stem cells to neuroectoderm. RB expression was analysed by Q-PCR. Ct values correspond to pRb, Rbl1 or Rbl2 expression in hPSCs. (B-C) RBs have different levels of expression in hPSCs. Western blot of RBs. Breast cancer lines were used as a comparison for relative protein expression. (C) Quantification of RB western blots by protein densitometry. (D) Immunostaining of RBs and germ layer specific markers during differentiation into endoderm, mesoderm and neuroectoderm. Scale bar 100 µm. (E) Effects of RB knockdown on background differentiation of hPSCs to neuroectoderm. Differentiation marker analysis by Q-PCR. (F) Effects of RBL2 knockdown on SOX1 and PAX6 expression in Tra-1-60 positive and negative cells. Cells were sorted or Tra-1-60 expression and analysed by qPCR. (G) Relative expression of pRb and Rbl1 in stable overexpression clones. All data are shown as mean+/-s.d. (n=3 biological replicates). Statistical analysis was performed by 2-way ANOVA with multiple comparisons with Tukey correction and **** marks adjusted P-value <0.0001, *** is adjusted P-value <0.001, ** is adjusted P-value <0.01, * is adjusted P-value <0.05.

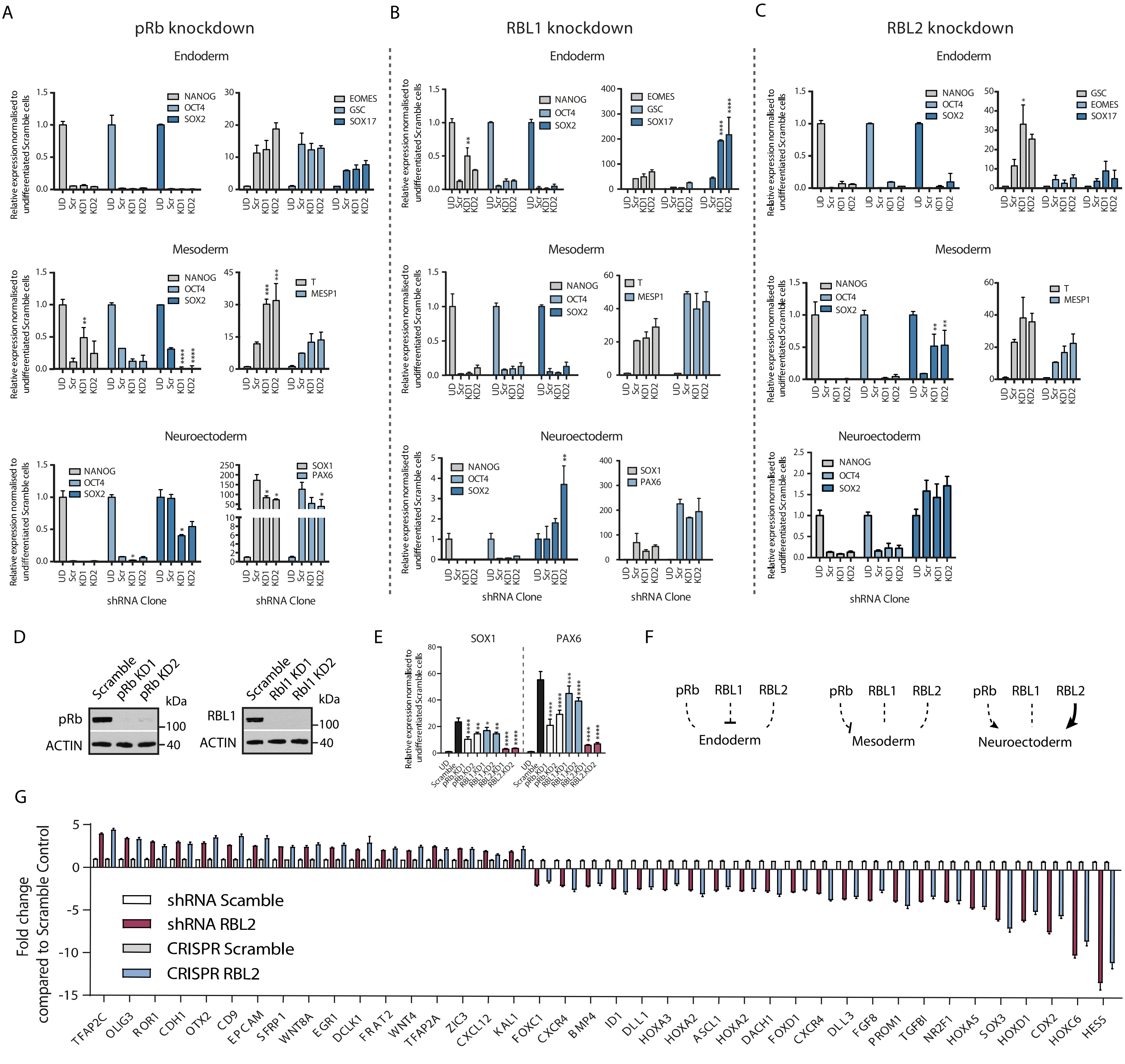

Figure S2: Analysis of RB KD on germ layer specification indicates a distinct function for RBL2 in neuroectoderm formation, Related to Figure 2. (A-C) Effect of RB KD during differentiation into three germ layers. Analysis of pluripotency and differentiation markers in (A) Rb KD cells, (B) RBL1 KD cells and (C) RBL2 KD cells. Significant differences compared to differentiated Scramble shRNA cells calculated by two-way ANOVA are marked. (D) Confirmation of pRb and RBL1 knockdown in stable knockdown HESCs. Western blot analysis of two knockdown lines compared to Scramble shRNA cells. (E) Comparison of the effects of Rb genes on neuroectoderm differentiation. Scramble and RB knockdown cells were side-by-side differentiated to neuroectoderm for 9 days and analysed by Q-PCR. Significant differences compared to differentiated Scramble shRNA cells calculated by two-way ANOVA are marked. (F) Schematic overview of the effects of Retinoblastoma-family proteins on germ layer differentiation. (G) Comparison of RBL2 knockdown effects on gene expression in RBL2 knockdowns achived by shRNA and CRISPR/Cas9. Cells were collected at day 4 of neuroectoderm differentiation and gene expression was normalised to control Scramble cells. All data are shown as mean+/-s.d. (n=3 biological replicates). Statistical analysis was performed by 2-way ANOVA with multiple comparisons with Tukey correction and **** marks adjusted P-value <0.0001, *** is adjusted P-value <0.001, ** is adjusted P-value <0.01, * is adjusted P-value <0.05.

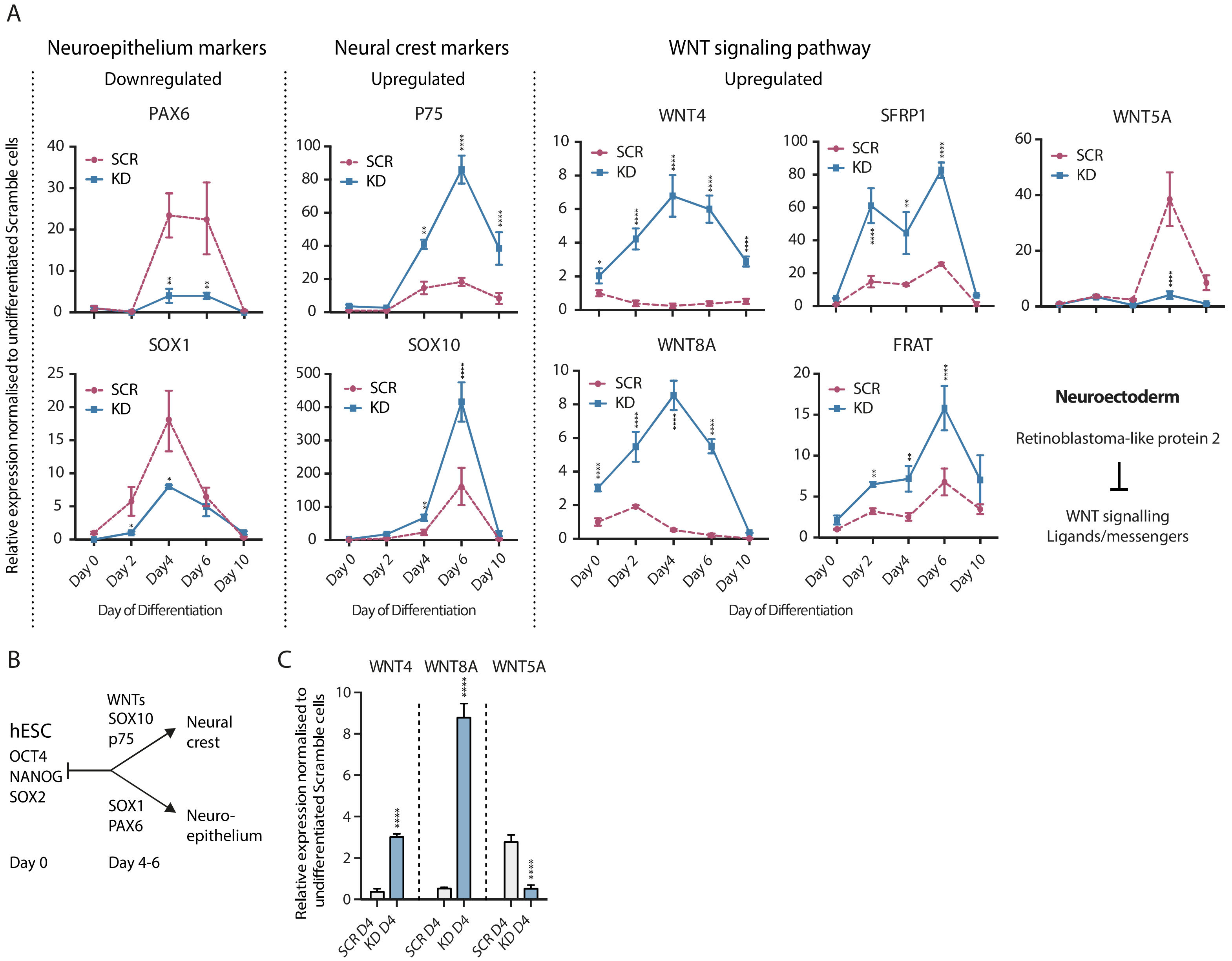

Figure S3: RBL2 represses WNT ligands WNT4 and WNT8A but leads to increased p75 and SOX10 expression, Related to Figure 2. (A) RBL2 controls the balance between neuroepithelial versus neural crest specification. Q-PCR throughout neuroectoderm differentiation of neuroepithelial markers SOX1, PAX6 and neural crest markers markers p75 and SOX10. Significant differences compared to Scramble shRNA cells calculated by two-way ANOVA are marked. (B) Schematic overview of the differentiation process and the markers characterising different cell types. (C) RBL2 KD increases the expression of canonical WNT pathway ligands WNT4 and WNT8A but decreases non-canonical WNT pathway ligand WNT5A during neuroectoderm differentiation. Significant differences compared to day 4 neuroectoderm Scramble shRNA cells calculated by two-way ANOVA are marked. All data are shown as mean+/-s.d. (n=3 biological replicates). Statistical analysis was performed by 2-way ANOVA with multiple comparisons with Tukey correction and **** marks adjusted P-value <0.0001, *** is adjusted P-value <0.001, ** is adjusted P-value <0.01, * is adjusted P-value <0.05.

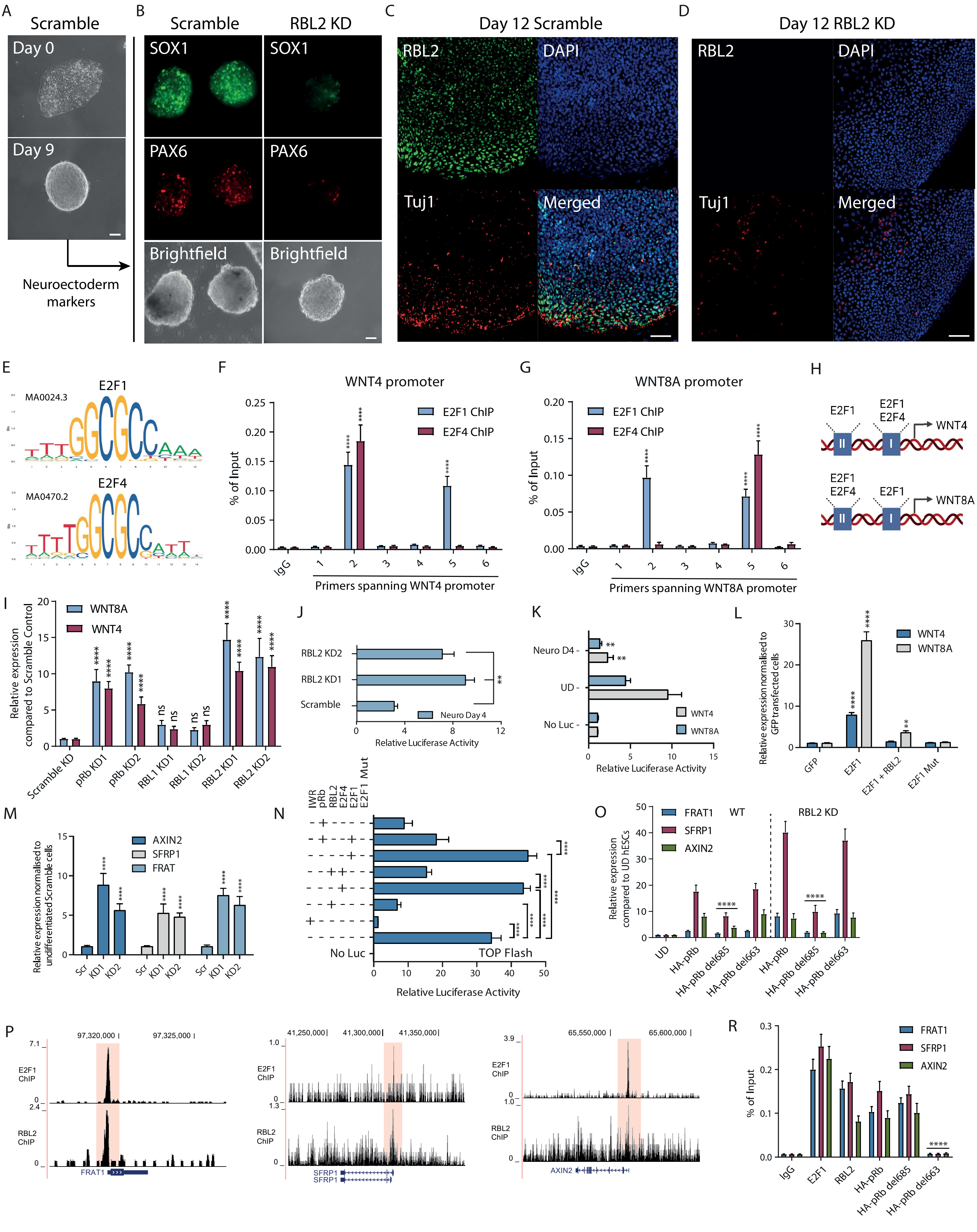

A
Scramble
Day 0
Day 9
Neuroectoderm markers
B
Scramble
RBL2 KD
SOX1
PAX6
Brightfield
C
Day 12 Scramble
RBL2
DAPI
Tuj1
Merged
D
Day 12 RBL2 KD
E
E2F1
MA0024.3
E2F4
MA0470.2
F
WNT4 promoter
% of Input
E2F1 ChIP
E2F4 ChIP
IgG
Primers spanning WNT4 promoter
G
WNT8A promoter
Primers spanning WNT8A promoter
H
E2F1
E2F4
WNT4
WNT8A
I
WNT8A
WNT4
Relative expression compared to Scramble Control
Scramble KD
pRb KD1
pRb KD2
RBL1 KD1
RBL1 KD2
RBL2 KD1
RBL2 KD2
ns
J
RBL2 KD2
RBL2 KD1
Scramble
Neuro Day 4
Relative Luciferase Activity
K
Neuro D4 -
UD -
No Luc -
WNT4
WNT8A
Relative Luciferase Activity
L
Relative expression normalised to GFP transfected cells
WNT4
WNT8A
GFP
E2F1
E2F1 + RBL2
E2F1 Mut
M
Relative expression normalised to undifferentiated Scramble cells
AXIN2
SFRP1
FRAT
Scr
KD1
KD2
N
IWR
pRb
RBL2
E2F4
E2F1
E2F1 Mut
No Luc
TOP Flash
Relative Luciferase Activity
O
FRAT1
SFRP1
AXIN2
WT
RBL2 KD
Relative expression compared to UD hESCs
UD
HA-pRb
HA-pRb del685
HA-pRb del663
P
E2F1 ChIP
RBL2 ChIP
FRAT1
SFRP1
AXIN2
R
% of Input
FRAT1
SFRP1
AXIN2
IgG
E2F1
RBL2
HA-pRb
HA-pRb del685
HA-pRb del663

Figure S4: RBL2 represses WNT ligands WNT4 and WNT8A through their proximal promoter regions, Related to Figure 3. (A) Representative images of pluripotent H9 hESCs at day 0 and neural organoids at day 9. Scale bar 50 µm. (B) Immunostaining of SOX1 and PAX6 proteins in neural organoids at day 9 shows reduced expression of SOX1 and PAX6 in RBL2 KD cells. Scale bar 50 µm. (C) RBL2 expression is elevated in Tuj1 expressing cells in day 12 organoids. Scale bar 50 µm. (D) RBL2 KD causes spatial effects on Tuj1 expressing cell patterning of day 12 organoids compared to control Scramble cells (C). Scale bar 50 µm. (E) The similarity of E2F1 and E2F4 binding motifs. (F-G) E2F1 and E2F4 binding to two sites on WNT4 and WNT8A loci. (H) Schematic model of E2F1 and E2F4 competitive binding on WNT4 and WNT8A loci. (I) Comparison of pRb, RBL1 and RBL2 knockdown effects on WNT8A and WNT4 expression. (J) β-catenin is transcriptionally more active in RBL2 KD cells. Scramble and RBL2 KD cells were transfected with a TOP Flash luciferase construct containing a β-catenin responsive element. Significant differences compared to day 4 neuroectoderm Scramble shRNA cells calculated by two-way ANOVA are marked. (K) Relative promoter activity of WNT4 and WNT8A in HESCs and day 4 neuroectoderm cells. Luciferase assay of promoter constructs. Significant differences compared to undifferentiated (UD) HESCs calculated by two-way ANOVA are marked. (L) E2F1 induced endogenous WNT4 and WNT8A expression while RBL2 represses this effect. Cells transfected with E2F1, RBL2 and E2F1 mutant constructs were analysed 48 hours after transfection by Q-PCR. Significant differences compared to GFP transfected cells calculated by two-way ANOVA are marked. (M) RBL2 KD results in upregulation of known target genes of β-catenin. Gene expression was analysed by Q-PCR. Significant differences compared to day 4 neuroectoderm Scramble shRNA cells calculated by two-way ANOVA are marked. (N) RBL2 regulates the activity of β-catenin. RBL2 KD cells were cotransfected with a TOP Flash luciferase construct containing a β-catenin responsive element and a combination of expression constructs for RBL2 and E2F proteins. (O) Effects of pRb domain mutants on the expression of WNT pathway components FRAT1, SFRP1 and AXIN2. (P) Genomic views of E2F1 and RBL2 binding on FRAT1, SFRP1 and AXIN2. (R) The binding of E2F1, RBL2 and pRb mutants to FRAT1, SFRP1 and AXIN2 loci. All data are shown as mean+/-s.d. (n=3 biological replicates). Statistical analysis was performed by 2-way ANOVA with multiple comparisons with Tukey correction and **** marks adjusted P-value <0.0001, *** is adjusted P-value <0.001, ** is adjusted P-value <0.01, * is adjusted P-value <0.05.

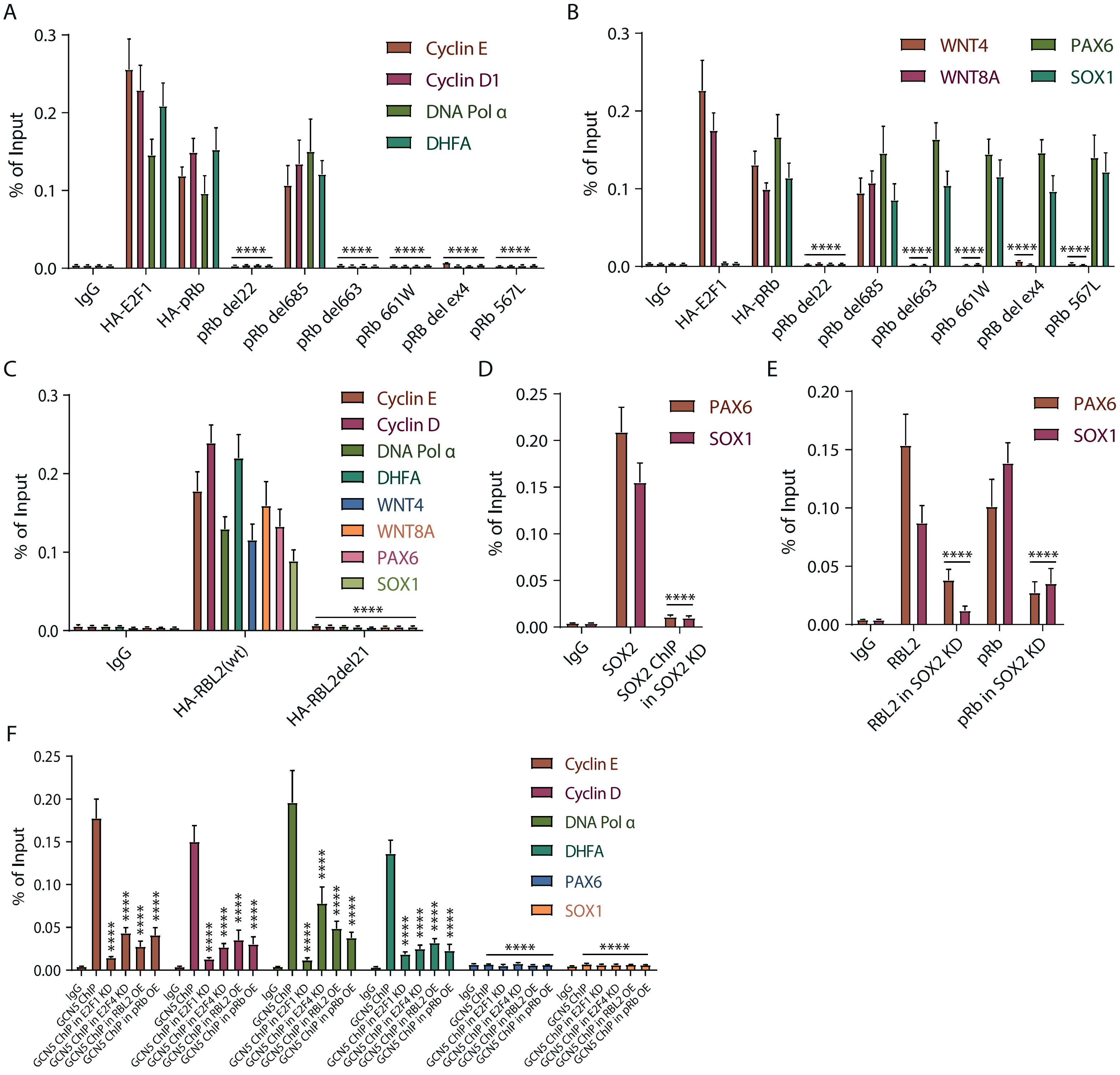

Figure S5: Characterising the regulation of RB target genes involved in cell cycle regulation and neuroectoderm differentiation, Related to Figure 3. (A) The binding of E2F1 and pRb mutants to cell cycle regulatory loci Cyclin E, Cyclin D1, DNA Pol α and DHFA loci. (B) The binding of E2F1 and pRb mutants to WNT ligand loci WNT4, WNT8A, and neuroectoderm differentiation genes PAX6 and SOX1. (C) RBL2 binds to cell cycle regulatory loci, WNT4, WN8A and neuroectoderm genes PAX6 and SOX1. (D) SOX2 binds to PAX6 and SOX1 loci during neuroectoderm differentiation at day 4. (E) SOX2 recruits RBL2 to PAX6 and SOX1. (F) GCN5 competes with RBL2 and pRb to bind to cell cycle regulatory loci via E2F1/4. All data are shown as mean+/-s.d. (n=3 biological replicates). Statistical analysis was performed by 2-way ANOVA with multiple comparisons with Tukey correction and **** marks adjusted P-value <0.0001, *** is adjusted P-value <0.001, ** is adjusted P-value <0.01, * is adjusted P-value <0.05.

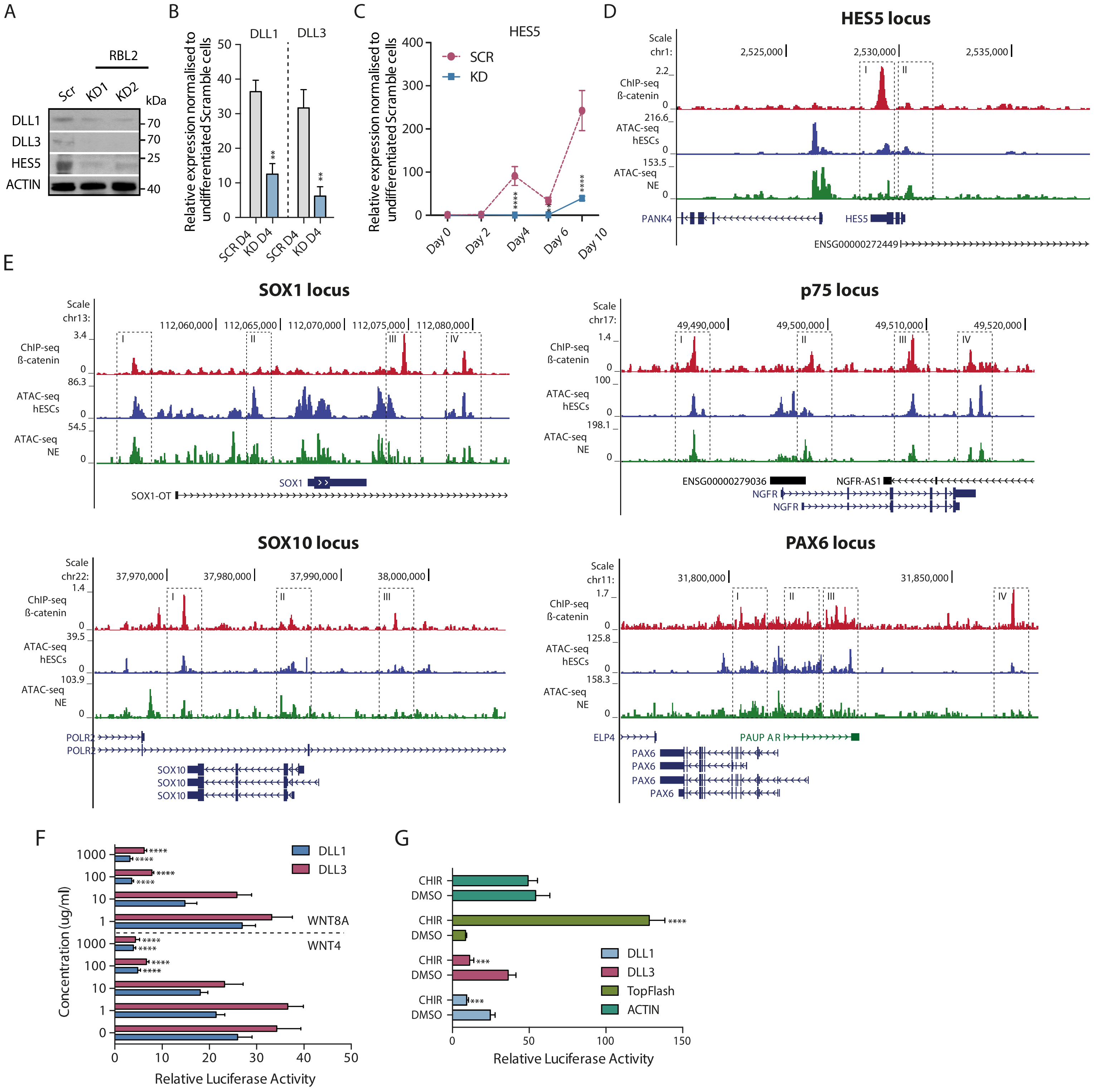

Figure S6: RBL2 regulates cell fate specification between neuroepithelium and neural crest through WNT and NOTCH ligands, Related to Figure 4. (A) Expression of NOTCH ligands is decreased in the absence of RBL2. Western blot analysis of NOTCH ligands and NOTCH target gene HES5 in Scramble and RBL2 KD cells. (B-C) RBL2 KD reduces the expression of NOTCH ligands DLL1, DLL3 and their target gene HES5 during neuroectoderm specification. Q-PCR analysis of NOTCH pathway members (B) in day 4 neuroectoderm or (C) during the time course of neuroectoderm differentiation. Significant differences compared to Scramble shRNA cells calculated by two-way ANOVA are marked. (D-E) Genomic regions of DLL1 and DLL3 loci. Genomic region of HES5 locus (D) and SOX1, p75, SOX10 and PAX6 locus (E) showing β-catenin ChIP-seq binding data in a cancer cell line together with hESC and neuroectoderm differentiating cells analysed by ATAC-seq. Genomic regions showing β-catenin binding peaks are highlighted with numbered dashed boxes. (F) WNT/β-catenin signalling reduces the promoter activity of DLL1 and DLL3 genes. Cells transfected with NOTCH promoter constructs were treated with a range of WNT4/WNT8A concentrations for 24h. Significant differences compared to DMSO treated samples calculated by two-way ANOVA are marked. (G) β-catenin activation by GSK3 inhibitor CHIR reduces the promoter activities of NOTCH genes. Cells were treated with CHIR for 24h. All data are shown as mean+/-s.d. (n=3 biological replicates). Statistical analysis was performed by 2-way ANOVA with multiple comparisons with Tukey correction and **** marks adjusted P-value <0.0001, *** is adjusted P-value <0.001, ** is adjusted P-value <0.01, * is adjusted P-value <0.05.

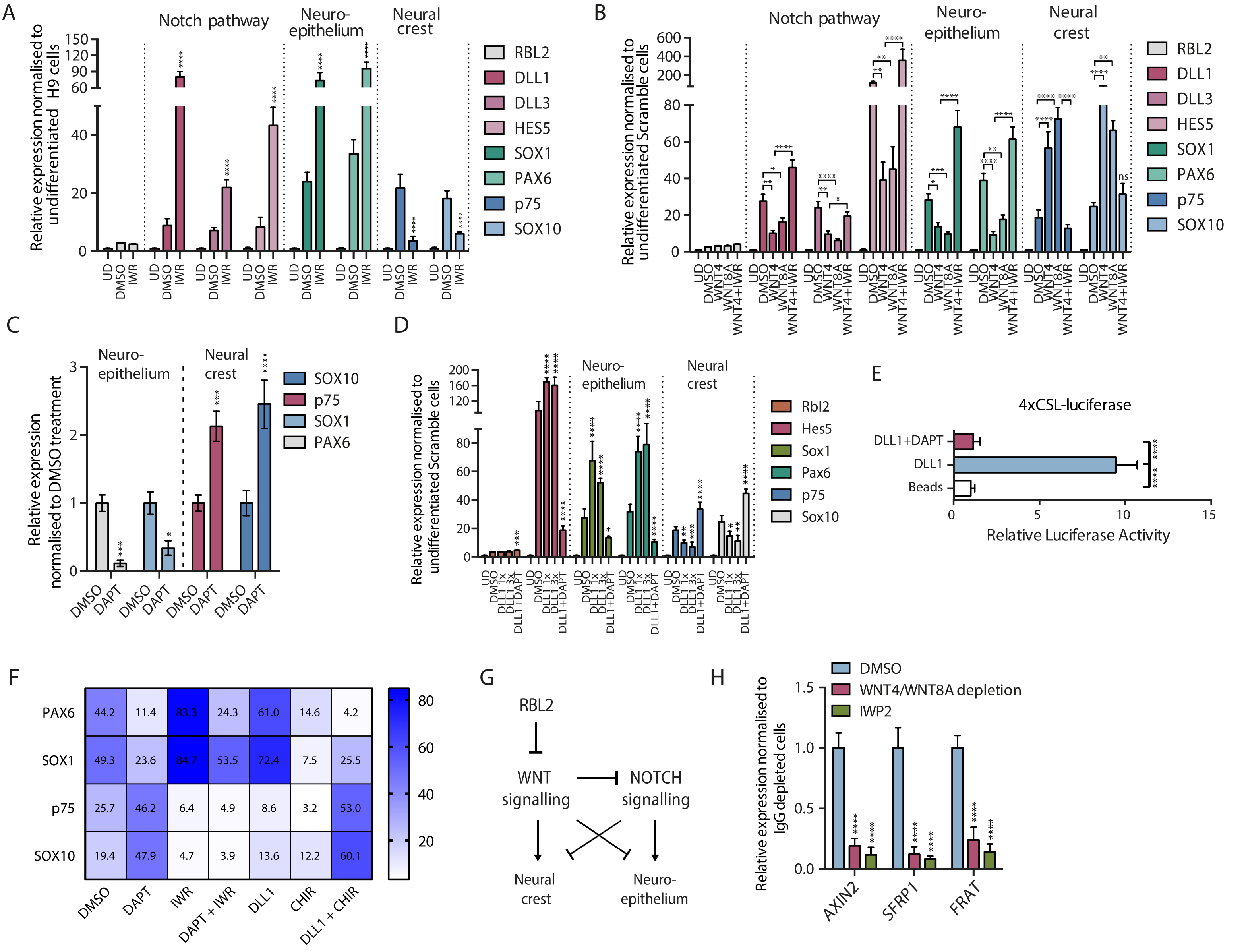

Figure S7: NOTCH signalling is indirectly regulated by RBL2 and mediates its effects on cell fate decisions in neuroectoderm, Related to Figure 4. (A) WNT inhibition reduces neural crest specification while increasing neuroepithelial fate and NOTCH pathway members. Day 3 neuroectoderm cells were treated for the days with WNT inhibitor and analysed by Q-PCR for NOTCH ligands, neuroepithelial markers and neural crest markers. Significant differences compared control DMSO treated cells calculated by two-way ANOVA are marked. (B) Purified WNT ligands WNT4 and WNT8A shift cellular specification toward neural crest fate while blocking NOTCH ligand expression. Differentiation marker analysis by Q-PCR after treatment of day 3 neuroectoderm cells with purified WNT4 and WNT8A for 3 days. (C) Inhibition of NOTCH signalling shifts the expression of developmental genes from neuroepithelial markers to neural crest markers. Q-PCR analysis of differentiation marker expression in day 4 neuroectoderm cells after NOTCH inhibition for 24h with 10µM DAPT. Significant differences compared to DMSO treated samples calculated by two-way ANOVA are marked. (D) Purified agarose-bound NOTCH ligand DLL1 shifts specification toward neuroepithelial cell fate. Differentiation marker analysis after treatment of cells with purified DLL1 for 24 hours. (E) Confirmation of NOTCH signalling activation by agarose-bound DLL1. Cells were transfected with a NOTCH responsive 4xCSL-luciferase construct were treated with agarose-bound DLL1 alone or with 10µM DAPT for 24h. Significant differences compared to control agarose beads calculated by two-way ANOVA are marked. (F) WNT and NOTCH cooperate to regulate the cell fate choice between neuroepithelium and neural crest. Cells were differentiated to day 3 neuroectoderm and then treated with WNT/NOTCH activators and inhibitors for 3 days followed by Q-PCR analysis. Significant differences compared to DMSO treated samples calculated by two-way ANOVA are marked. (G) Schematic overview of NOTCH signalling in regulating cell fate decisions. (H) Confirmation of WNT4/WNT8A depletion on the expression of known β-catenin target genes. Cells were incubated for 24h with media depleted from WNT4/WNT8A proteins or conditioned with WNT inhibitor IWP2 and analysed by Q-PCR. All data are shown as mean+/-s.d. (n=3 biological replicates). Statistical analysis was performed by 2-way ANOVA with multiple comparisons with Tukey correction and **** marks adjusted P-value <0.0001, *** is adjusted P-value <0.001, ** is adjusted P-value <0.01, * is adjusted P-value <0.05.
