# Supplementary material for: RBL2-E2F-GCN5 guide cell fate decisions during tissue specification by regulating cell-cycle-dependent fluctuations of non-cell-autonomous signaling
Source: Cell Rep. 2023 Sep 19;42(9):113146. doi: 10.1016/j.celrep.2023.113146 (PMC12823551; doi:10.1016/j.celrep.2023.113146)
Supplement: Document S2. Article plus supplemental information [file mmc4.pdf]

Cell Reports

Article

RBL2-E2F-GCN5 guide cell fate decisions during tissue specification by regulating cell-cycle-dependent fluctuations of non-cell-autonomous signaling

Graphical abstract

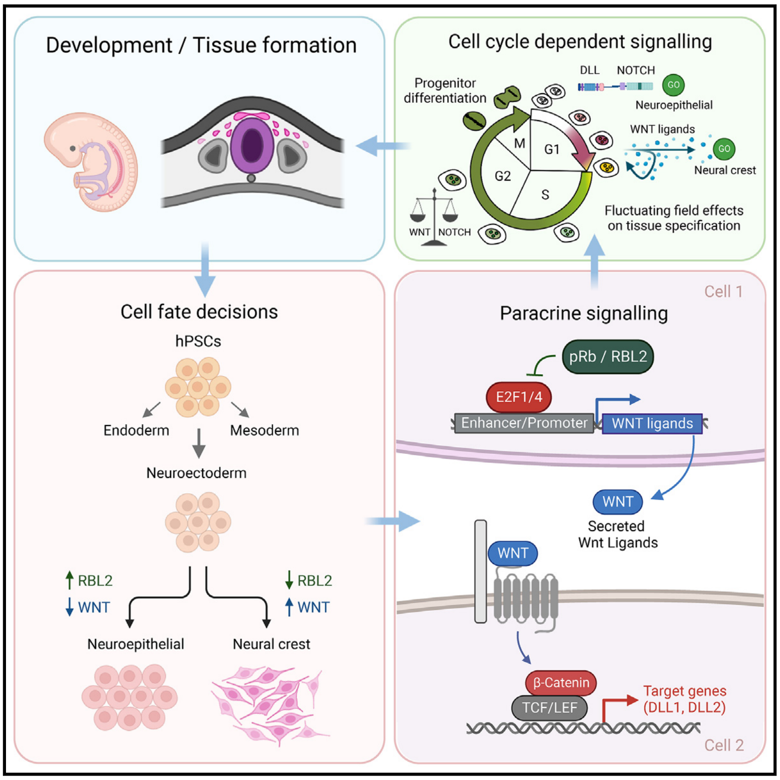

Highlights

- RBL2-E2F-GCN5 cell-autonomous axis regulates tissue formation non-cell autonomously
- RBL2-E2F cause temporal fluctuations of WNT/β-catenin and DLL/NOTCH signaling
- E2Fs-GCN5 induce WNTs in late G1 while E2Fs-RBs repress in early G1 to guide specification
- RBL2-E2F-GCN5 activity has field effects on surrounding cells through paracrine signaling

Authors

Stefania Militi, Reshma Nibhani, Morteza Jalali, Siim Pauklin

Correspondence

siim.pauklin@ndorms.ox.ac.uk

In brief

Militi et al. identified a function for the RB-E2F bona fide cell-autonomous cell-cycle regulatory axis in cell fate decisions by showing that RBL2 acts a regulator of field effects via WNT ligands. This reveals an unanticipated role of the RBL2/p130-E2F axis in stem cell and tissue progenitor differentiation.

Militi et al., 2023, Cell Reports *42*, 113146
September 26, 2023 © 2023 The Authors.
https://doi.org/10.1016/j.celrep.2023.113146

CellPress

Cell Reports

CellPress
OPEN ACCESS

Article

RBL2-E2F-GCN5 guide cell fate decisions during tissue specification by regulating cell-cycle-dependent fluctuations of non-cell-autonomous signaling

Stefania Militi,[1,4] Reshma Nibhani,[1,4] Morteza Jalali,[2] and Siim Pauklin[1,3,5,*]
[1]Botnar Research Centre, Nuffield Department of Orthopaedics, Rheumatology and Musculoskeletal Sciences, University of Oxford, Old Road, Headington, Oxford OX3 7LD, UK
[2]Anne McLaren Laboratory for Regenerative Medicine, Wellcome Trust-Medical Research Council Cambridge Stem Cell Institute, University of Cambridge, Cambridge, UK
[3]Senior author
[4]These authors contributed equally
[5]Lead contact
*Correspondence: siim.pauklin@ndorms.ox.ac.uk
https://doi.org/10.1016/j.celrep.2023.113146

SUMMARY

The retinoblastoma family proteins (RBs) and E2F transcription factors are cell-autonomous regulators of cell-cycle progression, but they also impact fate choice in addition to tumor suppression. The range of mechanisms involved remains to be uncovered. Here, we show that RBs, particularly RBL2/p130, repress WNT ligands such as WNT4 and WNT8A, thereby directing ectoderm specification between neural crest to neuroepithelium. RBL2 achieves this function through cell-cycle-dependent cooperation with E2Fs and GCN5 on the regulatory regions of WNT loci, which direct neuroepithelial versus neural crest specification by temporal fluctuations of WNT/β-catenin and DLL/NOTCH signaling activity. Thus, the RB-E2F bona fide cell-autonomous axis controls cell fate decisions, and RBL2 regulates field effects via WNT ligands. This reveals a non-cell-autonomous function of RBL2-E2F in stem cell and tissue progenitor differentiation that has broader implications for cell-cycle-dependent cell fate specification in organogenesis, adult stem cells, tissue homeostasis, and tumorigenesis.

INTRODUCTION

The retinoblastoma family of tumor suppressors (RBs: pRb, RBL1, and RBL2) are pivotal for cell-cycle progression in mammalian cells. They control the G1 to S phase transition by reducing the transcriptional activity of E2F proteins (E2Fs), thereby leading to transcriptional repression of target genes necessary for proliferation. In turn, the phosphorylation of RBs by cyclin D/CDK4-6 blocks interactions with E2Fs, permitting the induction of E2F-mediated transcription.[1–4] Mutations in RBs are known to be important in causing tumorigenesis, and, accordingly, such genetic aberrations are found in various human cancers including breast, pancreas, lung, blood, and brain malignancies.[5–11] Besides their tumor suppressive function, RBs are also central to early mammalian development. Genetic studies in the mouse have shown that the absence of pRb is embryonic lethal between embryonic day 13 (E13) and E15 of gestation due to abnormal hematopoietic, neuronal, and eye lens development provoked by defects in cellular differentiation.[12–17] pRb regulates lineage specification of osteoblasts and adipocytes by promoting the activity of developmental transcription factors such as Runx2 in osteogenic differentiation,[18,19] while it acts with E2F to suppress peroxisome proliferator-activated receptor c subunitIZI, the master activator of adipogenesis.[20,21] pRb also cooperates with the developmental transcription factor MyoD in myogenic differentiation.[22,23] Therefore, pRb seems to direct the differentiation of different cell types by controlling the activity of master regulators of differentiation.

In contrast, the function of the other members of the RB family in differentiation is less established. RBL1−/− mice are viable and fertile but have impaired growth and exert myeloid hyperplasia.[24] More strikingly, RBL2−/− embryos die between E11 and E13 due to disorganization in neural and dermatomyotomal structures,[25] which is characterized by reduced numbers of neurons in the spinal cord and the dorsal root ganglia and by decreased myocytes in the myotome. Furthermore, biallelic loss-of-function variants of RBL2 have been identified in humans with a neurodevelopmental disorder.[26] Thus, RBL1/2 are likely to have a function in cell fate decision, which might not directly overlap with pRb. However, second-site modifier genes that still exist have an epistatic relationship with RBL2, because RBL2 plays an essential role in normal development in the Balb/cJ genetic background in mice, but not in C57BL/6J strain. Mice lacking either RBL1 or RBL2 in a mixed 129/Sv:C57BL/6J genetic background have no overt phenotype and are viable and fertile.[27–30] Embryos lacking both pRb and RBL1 die *in utero* 2 days earlier than pRb-deficient embryos and exhibit apoptosis in the liver and central nervous system, suggesting some redundancy in function. Mutant mice

This is an open access article under the CC BY license (http://creativecommons.org/licenses/by/4.0/).

lacking both RBL2 and RBL1 die soon after birth and exhibit defective endochondral bone development. Taken together, these data suggest that RBL1/p107 and RBL2/p130 have relatively subtle roles in regulating the cell cycle and that a significant degree of overlap in function exists between the proteins.[27,30]

Interestingly, the targeted disruption of the three Rb-related genes in mouse embryonic stem cells (mESCs) strongly alters their capacity of differentiation, while the absence of RB protein function in human embryonic stem cells (hESCs) induces cell death.[31] Moreover, mouse embryonic fibroblasts with a knockout for the three RB genes display a loss of G1 control and cellular immortalization[32,33] that can be regarded as pathological self-renewal, which resembles the physiological self-renewal processes observed in embryonic or adult tissue-specific stem cells.[34] In line with its function in controlling the self-renewal properties of stem cells or progenitors, pRb can restrict reprogramming and tumorigenesis by inhibiting pluripotency networks.[35] Considered together, these reports strongly suggest a function for RBs in cell fate decisions of stem cells and progenitors during embryonic development and in adult organs. Nonetheless, the precise mechanisms by which RB proteins achieve this function remain unknown.

To further investigate these mechanisms, we decided to study the function of RB family tumor suppressor genes in hESCs and human induced pluripotent stem cells (hiPSCs). Pluripotent stem cells represent a useful *in vitro* model to examine cell fate decisions since their differentiation into the three primary germ layers—endoderm, mesoderm, and neuroectoderm—can be precisely controlled using a combination of growth factors in defined culture conditions.[36–40] Therefore, we utilized this system to investigate the molecular function of RBs in early cell fate decisions in both 2D cultures and the 3D organoid system,[41,42] the latter being particularly useful for early human development, which has been challenging to investigate for ethical reasons, inaccessibility of fetal tissue, and possible differences between human and conventional model systems like mouse.

Combining this approach with functional studies and molecular analyses, we uncovered that RBL2 controls neuroectoderm differentiation of hESCs through a paracrine mechanism, which involves the transcriptional repression of WNT ligands by the RBL2/pRb-E2F4/1 complex and General control non-depressible 5 (GCN5) histone acetyltransferase enzyme. Besides the classical cell-autonomous tumor suppressor function of RB-E2F axis, our results show non-cell-autonomous effects arising as a consequence of cell-cycle-dependent RBL2/pRb-E2F4/1 function that guides cell fate specification through temporal fluctuations of WNT/β-catenin and DLL/NOTCH developmental signaling pathways. This non-cell-autonomous function represents a function of RB-E2F tumor suppressor axis in stem cell and tissue progenitor differentiation that has broader implications for cell fate specification in organogenesis, adult stem cells and tissue homeostasis.

RESULTS

RBs in human PSCs

To investigate the role of RBs in early cell fate decisions, we first studied their expression in hESCs grown in defined culture conditions inductive for neuroectoderm, mesoderm, and endoderm differentiation. Immunostaining, qPCR, and western blot analyses revealed that pRb and RBL1 are co-expressed with pluripotency factors in hESCs, while RBL2 cDNA and protein has the lowest expression in pluripotent cells (Figures 1A, 1B, and S1A–S1C). Furthermore, pRb and RBL1 are expressed at relatively constant levels during the differentiation of hESCs into any of the three germ layers, while RBL2 expression at protein level increases specifically during neuroectoderm formation, similarly to a more modest increase for RBL1 (Figures 1A, 1B, and S1D). Therefore, RBL2 in particular has an increased expression in neuroectoderm, suggesting a role in the neuroectoderm germ layer.

To explore the function of RBs, we generated knockdown lines for each RB (RB-KD: pRb-KD, RBL1-KD, RBL2-KD) (Figure 1C) and analyzed their effects on undifferentiated cells and on germ layer formation. Decreased RB expression did not alter the self-renewal or the morphology of RB-KD-hESCs when compared with control (Figure 1D). However, we noticed germ-layer-specific changes in spontaneous background differentiation propensity of RB-KDs distinctly for neuroectoderm lineage as shown by the loss of SOX1 and PAX6 (Figure S1E). On the other hand, endoderm (EOMES, SOX17, GOOSECOID) and mesoderm (T, MESP1) markers showed only a modest reduction in their background expression, which was not statistically significant compared with Scramble control cells (data not shown). We further investigated the expression of SOX1 and PAX6 to find out if their expression is changing in pluripotent cells or the spontaneously differentiated cells. For this, we analyzed SOX1 and PAX6 expression by qPCR in Tra-1-60-positive and -negative cells using fluorescence-activated cell sorting (FACS; Figure S1F). We saw no significant changes in the expression of these markers in Tra-1-60+ RBL2-KD cells compared with Tra-1-60+ Scramble hESCs, whereas there was a difference in Tra-1-60− Scramble versus RBL2-KD cells, indicating that there indeed is a change in the background spontaneously differentiating cells but not the pluripotent undifferentiated hESCs.

Since the neuroectoderm markers Sox1 and Pax6 were most strongly decreased in RBL2-KD and to a lesser extent in RBL1-KD and pRb-KD, this prompted us to analyze gene expression at the genome-wide scale in undifferentiated RBL2-KDs to get a broader understanding on transcriptional changes (Figure 1E). Kyoto Encyclopedia of Genes and Genomes (KEGG) pathway analyses of transcriptomic data indicated that RBL2-KD results in upregulation of processes involving ubiquitin-mediated proteolysis and apoptosis (Table S1), which have also been observed for RB family members previously,[31] and cell motion according to gene ontology analysis. Furthermore, RBL2-KD cells showed an elevated expression of genes involved in the WNT developmental signaling pathway (Figures 1E and 1F). While the canonical WNT signaling components WNT4 and WNT8A were increased upon RBL2 loss as shown by gene enrichment analysis, the non-canonical pathway member WNT5A was decreased (Figures 1E and 1F), suggesting that RBL2 function may alter the balance between canonical to non-canonical WNT activity. In contrast to the canonical WNT pathway, the NOTCH pathway components such as Delta-like 1 (DLL1) and Delta-like 3 (DLL3) and a number of Hox genes were decreased

CellPress
OPEN ACCESS

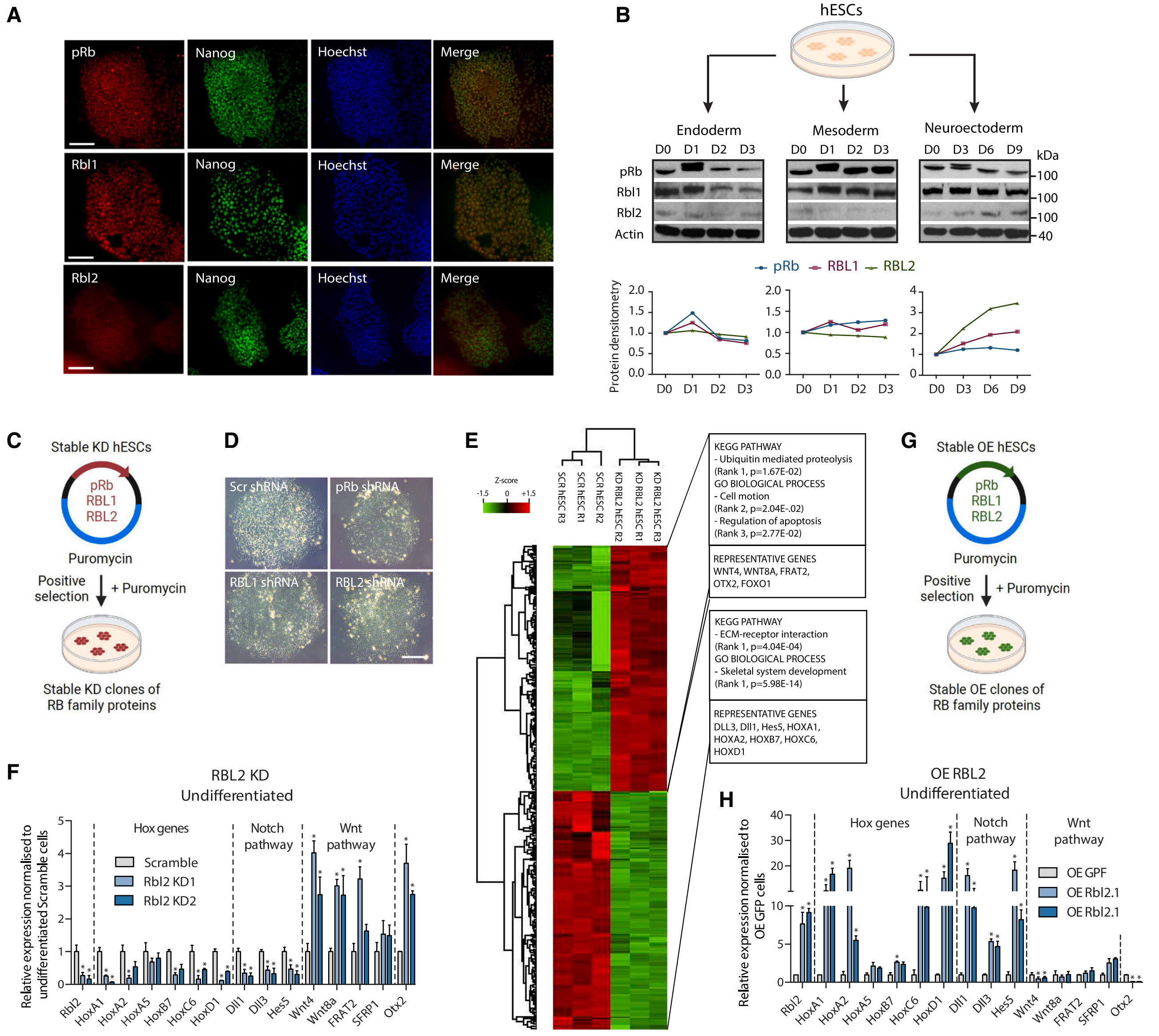

Figure 1. Retinoblastoma family proteins have distinct effects on cell fate specification of hPSCs

(A) RBs are expressed in hPSCs at varying levels. Scale bar, 100 μm.

(B) RBL2 has a distinct expression pattern during differentiation of hPSCs to endoderm, mesoderm, and neuroectoderm.

(C) Schematic overview of RBs knockdown.

(D) Morphology of hPSCs with a stable knockdown of RBs. Representative colonies of Scramble and RB-KD hPSCs. Scale bar, 100 μm.

(E) RBL2 KD causes a change in gene expression of developmental signaling pathways and differentiation markers.

(F) RBL2 KD alters the expression of Wnt, Hox, and Notch genes in hPSC culture. qPCR analysis of markers in RBL2-KDs.

(G) Schematic overview of overexpressing RBs.

(H) RBL2 OE causes background differentiation of hPSCs opposite to RBL2 KD. qPCR analysis of developmental markers in RBL2-OE hPSC culture. All data are shown as mean ± SD (n = 3). Statistical analysis was performed by two-way ANOVA with multiple comparisons with Tukey correction; ****$p_{adjusted} < 0.0001$, ***$p_{adjusted} < 0.001$, **$p_{adjusted} < 0.01$, *$p_{adjusted} < 0.05$. See also Figure S1.

(Figures 1E and 1F; Table S1). The WNT and NOTCH pathways, as well as Hox genes, are all key regulators of tissue formation during embryogenesis,[43,44] hence connecting RBL2 to developmental processes in an embryonic context.

To further verify the effects of RBL2 on gene expression, we performed stable overexpression of RBL2 (RBL2-OE) in human PSCs (hPSCs) (Figure 1G). This indicated the opposite effects to RBL2-KD by reducing the expression of WNT ligands in background spontaneously differentiating cells, while upregulating NOTCH ligands and several Hox genes (Figures 1H and S1G). Collectively, these results suggested that the expression of human RB family tumor suppressors does not abolish the

self-renewal capacity of hESCs, while RBL2 could impact the expression of WNT and NOTCH developmental signaling pathways and Hox genes in differentiating cells.

RBs have divergent functions during the differentiation of hPSCs into the primary germ layers

Next, we investigated the role of RBs in early cell fate decisions during neuroectoderm, mesoderm, and endoderm differentiation. During endoderm differentiation, pRb-KD did not significantly impact endoderm marker EOMES, GSC, and SOX17 expression or pluripotency markers (Figure S2A). RBL1-KD increased SOX17 expression and NANOG, but not EOMES, GSC, or pluripotency markers OCT4 and SOX2 (Figure S2B). RBL2-KD increased GSC expression, but not other markers (Figure S2C). In mesoderm differentiation, pRb-KD increased T and modestly MESP1, as well as NANOG, but decreased SOX2 expression (Figure S2A). RBL1-KD did not significantly impact mesoderm differentiation (Figure S2B), while RBL2-KD increased SOX2 in mesoderm (Figure S2C). During neuroectoderm, pRb-KD decreased SOX1, PAX6, and SOX2 (Figure S2A), while RBL1-KD did not significantly impact neuroectoderm differentiation (Figure S2B). On the other hand, RBL2-KD blocked the induction of SOX1 and PAX6 entirely during neuroectoderm differentiation (Figures 2A–2C, S2C, and S2E). However, the absence of these differentiation markers was not associated with a decrease of the neuroectoderm progenitor marker SOX2 (Figure S2C) or the upregulation of pluripotency markers OCT4/NANOG. Thus, reduction in RBL2 expression does not block neuroectoderm induction of hESCs but rather inhibits the progression of this differentiation toward a neuronal fate.

Taken together, these data suggest that RB proteins are individually dispensable for germ layer specification, which is in agreement with genetic studies in the mouse.[12–14] Nonetheless, the aberrant neuroectoderm differentiation observed upon RBL2 knockdown (Figure S2F) resembles the phenotype in mouse mutant for RBL2, where neural differentiation is also strongly affected.[24] Thus, our results obtained in hESCs could be conserved *in vivo* during early embryonic development.

RBL2 regulates neuronal versus neural crest cell fate during hPSC differentiation

These results prompted us to further delineate the nature of the cells focusing on RBL2-KD hESCs differentiating toward the neuroectoderm lineages. For that, we compared the transcriptomic profiles of RBL2-KD and KD-Scr hESCs differentiated toward neuroectoderm for 4 days. Enrichment analysis on the differentially expressed genes revealed that nervous system development was significantly suppressed by RBL2-KD ($p = 4.06E{-}7$, Figures 2E and 2F), thereby confirming at the genome-wide level that RBL2 is essential for neuroectoderm differentiation. To confirm the validity of the knockdown results, we compared the effects of RBL2/p130 depletion by short hairpin RNAs (shRNAs) and CRISPR-mediated repression of RBL2/p130 (Figure 2E). For all selected genes, we observed similar effects upon CRISPR-mediated RBL2 repression as for shRNA-mediated RBL2 repression (Figure S2G), indicating that the possibility of off-target effects by shRNAs in our results is negligible.

Detailed examination also revealed loss of neuroectoderm markers during differentiation of RBL2-KD cells, with a concomitant increase in neural crest regulators such as p75 and SOX10 (Figures 2D–2F).[45–48] These observations were validated by qPCR analyses, showing that the absence of RBL2 results in an increase in neural crest markers between days 4 and 10 of differentiation (Figures S3A and S3B). Therefore, the absence of RBL2 during neuroectoderm differentiation seems to induce a shift in the differentiation balance from neuroepithelium to neural crest fate.

Gene enrichment analyses also unearthed that the shift to neural crest gene expression was accompanied by upregulation of genes involved in controlling cell motion and chemotaxis (OTX2, PPAP2A, CD9, and DCLK1; Table S1). Cell migration is an important characteristic of neural crest cells[49] but also essential for metastatic processes and for the epithelial-to-mesenchymal transition that attributes malignant (stem cell-like) traits to cancer cells.[50] Thus, RBL2 could control mechanisms in early development, which may be related to its function as a tumor suppressor. Pathway analyses revealed that the mechanisms controlling neuroectoderm patterning were affected in RBL2-KD cells. RBL2-KD cells display an elevated expression of genes involved in the WNT canonical signaling pathway, such as WNT4 and WNT8A (Figures 2C and S3A–S3C; Table S1). In addition, DLL1 and DLL3 were decreased (Figures 2E; Table S1). Therefore, these results suggested that human RB family tumor suppressor RBL2 could block neural crest specification by controlling the expression of *WNT* and *NOTCH* genes, which are known to control the differentiation of the neuroectoderm germ layer *in vivo*.[43,44]

RBL2 controls the activity of the WNT signaling pathway during tissue specification by reducing the expression of WNT ligands

Next, we used organoids to characterize the effects of RBL2 in early tissue formation since it has emerged as a powerful model system for human development and disease, enabling genetic manipulation of 3D tissues.[41,42] Organoids are stem cell-derived miniature organs that recapitulate the cytoarchitecture of their *in vivo* counterparts in 3D tissue architecture and, thus, could reveal the impact of RBL2-KD on neural tissue specification (Figure 3A). We utilized H9 hESCs and three iPSC lines (KOLF, SFC841-03-01, and SFC840-03-03) for generating DOX-inducible RBL2KD cells via Cas9-KRAB-mediated transcriptional repression,[51] and neural induction by undirected differentiation in minimal medium followed by gene expression analysis to observe the effects of RBL2 in early neuroectodermal tissue specification (Figures 3B and 3C). Inducible knockdown of RBL2 led to an induction of a range of neural crest developmental genes, whereas RBL2-KD was accompanied by WNT8A and WNT4 upregulation in hESCs and the three iPSC lines (Figures 3B and 3C). RBL2 decreases WNT signaling activity during neuroectoderm specification (Figures 3D and 3E). These results are in line with neuroepithelial versus neural crest specification regulation by RBL2 in 2D conditions.

To study the relevance of spatial effects of RBL2-KD in neuroectodermal patterning, we first analyzed the expression of SOX1 and PAX6 in neuroectodermal organoids at day 9 (Figures S4A

CellPress
OPEN ACCESS

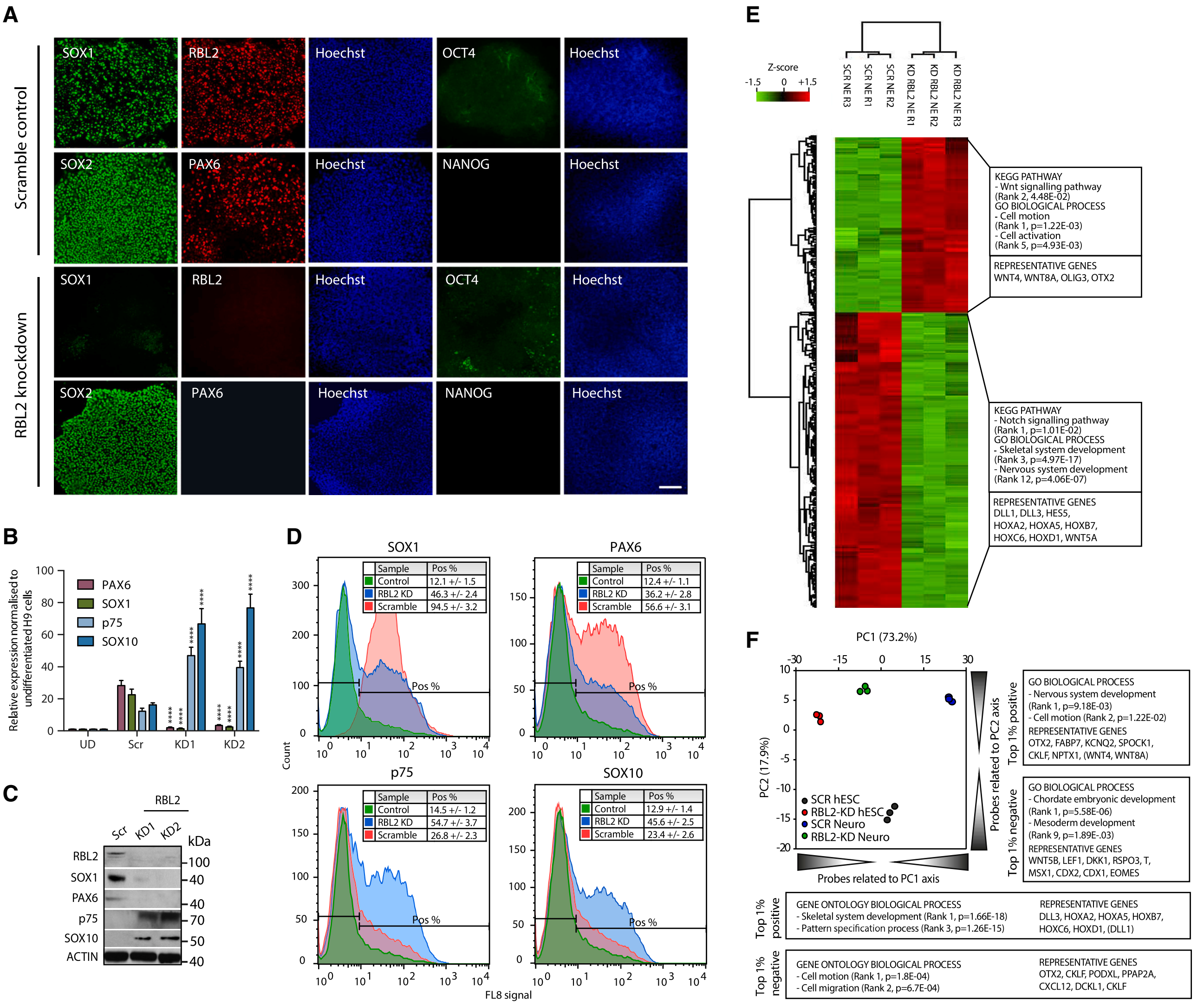

Figure 2. RBL2 regulates cell fate specification between neuroepithelium and neural crest

(A) RBL2 is necessary for neuroectoderm formation. Immunostaining of neuroectoderm markers in Scramble and RBL2 KD cells. Scale bar, 100 μm.

(B–D) RBL2 controls the balance between neuroepithelial versus neural crest specification. (B) qPCR, (C) western blot, and (D) flow cytometry analysis of SOX1, PAX6, p75, and SOX10 in RBL2 KD and Scramble at day 4 neuroectoderm.

(E) Loss of RBL2 in neuroectoderm specification changes the expression of Wnt and Notch pathway components and HoxA-D genes (see Table S1 for full results).

(F) Principal-component analysis (see Table S1 for full results). All data are shown as mean ± SD (n = 3). Statistical analysis was performed by two-way ANOVA with multiple comparisons with Tukey correction; ****$p_{adjusted} < 0.0001$, ***$p_{adjusted} < 0.001$, **$p_{adjusted} < 0.01$, *$p_{adjusted} < 0.05$. See also Figures S2 and S3.

and S4B). These results indicate a reduction of SOX1 and PAX6 expression upon RBL2-KD compared with Scramble control (Figure S4B), suggesting that dosage of RBL2 is critical for regulating neuroectodermal differentiation and/or patterning. We observed the start of ectoderm tissue patterning with a higher expression of RBL2 together with Tuj1 expression at day 12 of organoid growth, indicating elevated expression in neuronal identity-committed cells (Figures S4C and S4D). In contrast, RBL2-KD resulted in spatial disorganization of Tuj1-expressing cells compared with Scramble, indicating that RBL2-KD has spatial effects on early stages of neuroectodermal tissue patterning.

RBL2 is known to repress the transcriptional activity of E2F factors on target genes.[1] Consequently, we decided to delineate the primary mechanisms of RBL2-mediated cell fate decisions by focusing on the components of the WNT signaling pathway, which were upregulated upon RBL2-KD compared with wild-type (WT) cells. ATAC sequencing (ATAC-seq) data analysis from hESCs and neuroectoderm indicated the presence of E2F4 and E2F1 binding motifs at open chromatin regions on promoters nearby WNT8A and WNT4 loci (Figure 3F). E2F1 and E2F4 chromatin immunoprecipitation sequencing (ChIP-seq) data analyses from cancer cells (E2F1 ChIP-seq,[52,53] E2F4 ChIP-seq,[54] RBL2/p130[55]) further indicated the binding of

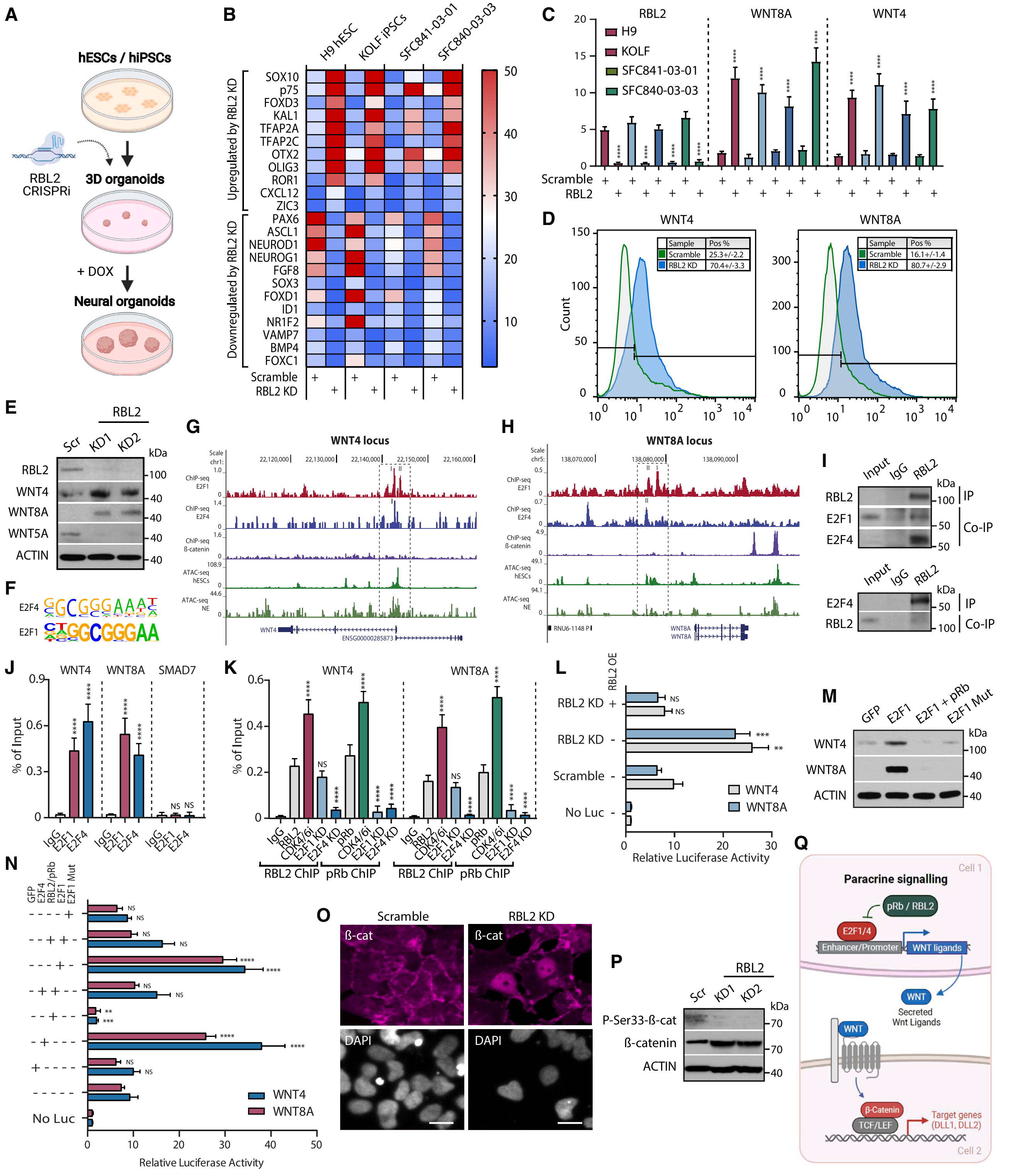

Figure 3. RBL2 represses WNT ligands by binding to regulatory regions in complex with E2F4 during neuroectoderm formation

(A) Schematic depiction of using CRISPRi of RBL2 in 3D neural organoids.

(B) CRISPRi-mediated RBL2-KD regulates neural tissue specification of hESC and hiPSC organoids by regulating key developmental genes.

(C) CRISPRi-mediated RBL2-KD in hESC and hiPSC organoids leads to increased WNT8A and WNT4 expression.

(D) WNT4 and WNT8A expression is elevated in RBL2-KD cells in day 6 neuroectoderm.

(legend continued on next page)

E2F1 and E2F4 in the proximity of WNT4 (Figure 3G) and WNT8A (Figure 3H). Considering the similarity of E2F1 and E2F4 binding motifs (Figure S4E), WNT4 and WNT8A have shared binding of E2F1 and E2F4 to their promoter region. Based on the ChIP-seq data, E2F1 is able to bind to two sites on WNT8A and two sites on WNT4 loci. These two E2F1 binding sites (marked with I and II in Figures 3G and 3H) are located relatively close to each other (~1 kb) in the proximity of transcription start sites of WNT4 and WNT8A loci. ChIP-seq data also indicate that E2F4 shares one of these binding sites with E2F1 on WNT8A promoter and WNT4 promoter (Figures 3G and 3H). We performed ChIP-qPCR experiments by using primers spanning the binding regions, and these verified the binding of E2F1 and E2F4 on the sites on WNT4 and WNT8A loci (Figures S4F and S4G). Our molecular model suggests a competitive binding of E2F1 and E2F4 on these sites at WNT4 and WNT8A promoters (Figure S4H).

Co-immunoprecipitation experiments in neuroectoderm cells suggested that RBL2 can be found in protein complexes containing E2F4 but much less abundantly with E2F1 (Figure 3I). Therefore, we next investigated the binding of E2F1, E2F4, and RBs to *WNT4* and *WNT8A* loci in hESCs differentiating to neuroectoderm in the organoid conditions. ChIP experiments followed by qPCR demonstrated that E2F4 and E2F1 bind to the proximal promoter regions on *WNT4* and *WNT8A* ligand loci (Figure 3J). Furthermore, RBL2 and pRb bind onto the same proximal promoter regions of WNT ligands *WNT4* and *WNT8A* (Figure 3K). CDK4/6 inhibition with the small-molecule inhibitor Palbociclib (PD-0332991) increased, whereas E2F1-KD reduced pRb binding and E2F4-KD reduced both RBL2 and pRb binding to *WNT4* and *WNT8A* loci (Figure 3K). We also compared the effect of depleting pRb, RBL1/p107, and RBL2/p130 on WNT8A and WNT4 expression as companion data for Figure 3K. The depletion of pRb and RBL2/p130 leads to the upregulation of WNT8A and WNT4 mRNA levels, whereas RBL1/p107 depletion has weaker effects (Figure S4I). These data suggest the presence of E2F4/1-RBL2 complexes on WNT4 and WNT8A genomic regions, and E2Fs recruit RBs onto chromatin.

Next, we generated *WNT4* and *WNT8A* promoter-luciferase constructs and co-transfected the resulting reporter genes into neuroectoderm cells generated from hESCs knocked down for RBL2 expression. *WNT4* and *WNT8A* promoters consistently showed higher activity in RBL2-KD cells compared with Scramble (Figure S4J), while RBL2 overexpression resulted in a decrease in the reporter's activity (Figure 3L). Similar results were obtained when the *WNT* reporter genes were transfected in WT hESCs and differentiated into neuroectoderm cells (Figure S4K). Interestingly, transfection of E2F1 was sufficient to increase the endogenous levels of WNT4 and WNT8A, and WNT signaling pathway components (Figures 3M and S4L), as well as the activity of *WNT* reporter genes (Figure 3N), while overexpression of RBL2 repressed the corresponding activation. Furthermore, an E2F1 mutant that is unable to bind DNA[56] did not induce the expression of these genes or promoter-luciferase constructs (Figures 3M and 3N). Taken together, these data suggest that RBL2 could repress the transcription of genes coding for WNT ligands by inhibiting the activity of E2Fs.

To further delineate the effects of RBL2 on β-catenin, we analyzed the activity of β-catenin during the differentiation of RBL2-KD hESCs. Immunostaining of endogenous β-catenin protein in RBL2 KD cells showed nuclear accumulation compared with Scramble cells (Figure 3O). Western blot analyses indicated that β-catenin phosphorylation on serine 33 (which destabilizes the protein and decreases its transcriptional activity) was lost in RBL2-KD neuroectoderm cells, while the total level of β-catenin was increased (Figure 3P), thereby suggesting that the increase in WNT ligand expression results in the upregulation of its downstream signaling pathway. Finally, E2F1 and E2F4 overexpression induced WNT reporter activity, while transient overexpression of RBL2 and pRb in the neuroectoderm inhibited these effects (Figure S4N), confirming the opposite functions of these transcription factors. Together, these data suggest that RBL2 and pRb control WNT signaling activity through the expression of WNT ligands during the differentiation of hPSCs toward the neuroectoderm lineages (Figure 3Q).

The RB family proteins participate in dual tumor suppressor functions, one linked to cell-cycle progression and the other to differentiation control.[57] The effects of cell-cycle gene regulation are mediated by the formation of RB/E2F/DNA complexes that are involved in gene expression repression, while pRB can cooperate with certain transcription factors to transcriptionally activate genes.[23,58–60] These functions are mediated by domains that are highly conserved between pRb, p107/RBL1, and p130/RBL2. Our experiments indicated that E2F1 and pRb/RBL2 form a complex on Wnt pathway components FRAT1, SFRP1, and AXIN2 loci (Figures S4O–S4R).

(E) RBL2-KD increases the expression of WNT4 and WNT8A during neuroectoderm differentiation.
(F) E2F4 and E2F1 binding motifs are found on open chromatin regions near WNT loci in hESCs by ATAC-seq analysis.
(G and H) Genomic regions of WNT4 and WNT8A loci. Genomic region of WNT4 locus (G) and WNT8A locus (H) showing E2F1, E2F4, β-catenin ChIP-seq binding with hESC and neuroectoderm differentiating cells analyzed by ATAC-seq. E2F1 and E2F4 binding peaks are highlighted with numbered dashed boxes.
(I) RBL2 forms a complex with E2Fs in neuroectoderm cells.
(J) E2F4 and E2F1 bind to promoter regions of WNT ligands in neuroectoderm analyzed by qPCR.
(K) RBL2 and pRb bind to promoter regions of WNT ligands.
(L) RBL2-KD causes derepression of WNT4 and WNT8A promoters.
(M and N) RBL2 regulates the transcription of WNT4 and WNT8A through a region in the proximity of the transcription start site. (M) Western blot analysis of intracellular WNT4 and WNT8A protein expression upon transfections of RBL2-KD cells. (N) RBL2-KD cells were cotransfected with WNT4 and WNT8A promoter constructs and OE constructs, and analyzed for luciferase activity.
(O) Stable RBL2-KD triggers nuclear accumulation of β-catenin protein. Scale bar, 10 μm.
(P) Endogenous β-catenin is less phosphorylated at Ser33 in RBL2-KD cells.
(Q) All data are shown as mean ± SD (n = 3). Statistical analysis was performed by two-way ANOVA with multiple comparisons with Tukey correction; $^{****}p_{adjusted} < 0.0001$, $^{***}p_{adjusted} < 0.001$, $^{**}p_{adjusted} < 0.01$, $^{*}p_{adjusted} < 0.05$. See also Figures S4 and S5.

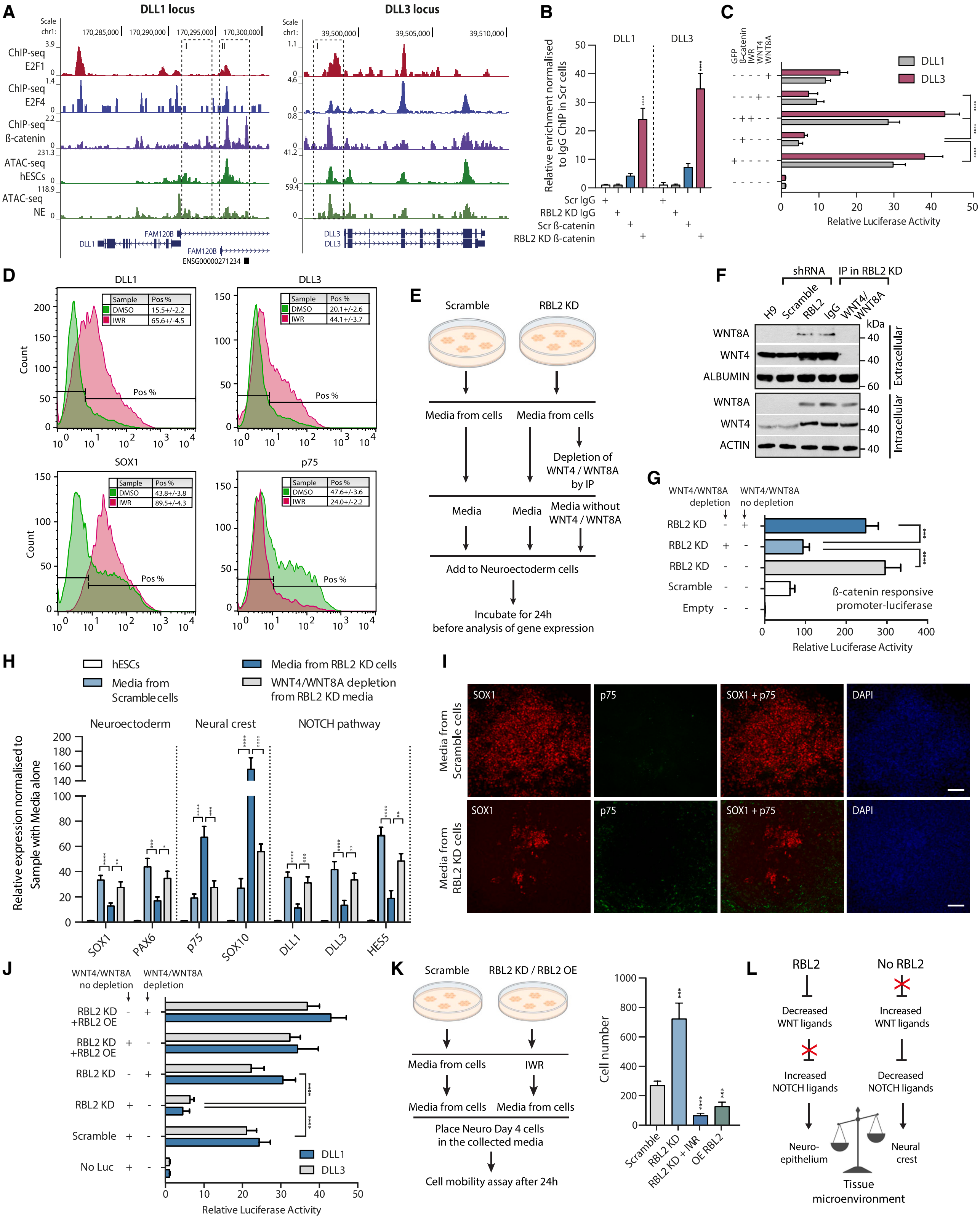

(legend on next page)

Next, we investigated the mechanism of how RBL2-KD affects specific cohorts of genes and whether the p130/RBL2-binding sites in the WNT8A and WNT4 promoters are acting in the same way or differently from the sites found on other genes.[61–64] The RB family proteins participate in dual tumor suppressor functions, one linked to cell-cycle progression and the other to differentiation control, and these functions can at least partially be genetically and mechanistically dissociated.[57] The functional domains are highly conserved also in RBL1 and RBL2 proteins.[57,65–69] Interestingly, E2F1, pRb wt, and pRb del685 bound to WNT4 and WNT8A loci as for cell-cycle regulatory genes (Figures S5A and S5B). Hence, WNT ligands could provide a link between coordination of cell-cycle progression and differentiation.

WT RBL2 bound to loci similarly to WT pRb and pRb del685 (Figure S5C). This raised the question of the identity of the sequence-specific transcription factor that facilitates binding of pRb mutants (e.g., pRb del685).[23,58–60,70] ATAC-seq, ChIP-seq, and ChIP-qPCR data suggested that SOX2 cooperates with RBL2 and pRb in promoting the expression of neuroectoderm differentiation factors PAX6 and SOX1 (Figures S5D and S5E). We also found that GCN5 binds to cyclin E, cyclin D1, DNA pol alpha, and DHFA regulatory regions to the same sites as E2F1 and E2F4 (Figure S5F).

Taken together, these data suggest that pRb and RBL2 perform two functions, namely, regulation of cell-cycle progression through its ability to repress E2F-dependent promoters (cell-cycle regulators and WNT4/WNT8A) and promotion of neuroepithelial differentiation through its ability to activate transcription in concert with non-E2F transcription factor SOX2.

RBL2 regulates cell fate decisions during tissue formation through WNT signaling, which in turn impacts NOTCH activity

Our transcriptomic analyses revealed that RBL2 loss is associated with a decrease in expression of NOTCH pathway ligands DLL1 and DLL3, as well as the downstream target HES5 (Figures S6A–S6C), suggesting that RBL2 may promote NOTCH ligand expression. However, RBL2 mainly functions as a transcriptional repressor, and thus, upregulation of NOTCH pathway genes could occur by an indirect mechanism. Interestingly, WNT/β-catenin signaling has been shown to control the expression of DLL1 and other NOTCH genes during development.[71,72] Thus, we hypothesized that RBL2 could regulate NOTCH signaling during neuroectoderm differentiation via the downregulation of canonical WNT ligands. To investigate this possibility, we analyzed β-catenin ChIP-seq data that indicated the binding of β-catenin to DLL1 and DLL3 loci (Figure 4A) and also in the proximity of *HES5*, *SOX1*, *PAX6*, *p75*, and *SOX10* loci (Figures S6D and S6E). Next, we performed β-catenin ChIP-QPCR in Scramble and RBL2-KD neuroectoderm cells and observed that the presence of the WNT effector on DLL1 and DLL3 loci strongly increased with RBL2 loss (Figure 4B). Moreover, promoter-luciferase constructs of DLL1 and DLL3 provided evidence that overexpression of β-catenin causes their transcriptional repression in the neuroectoderm context (Figure 4C), an effect that can be reversed by the WNT inhibitor IWR and mimicked by addition of purified WNT4 and WNT8A to the medium (Figures 4C and S6F) and by GSK3 inhibitor CHIR99021 (CHIR) (Figure S6G). Thus, the WNT/β-catenin pathway appears to control the expression of NOTCH signaling factors during neuroectoderm differentiation of hESCs.

To validate the functional interest of our observations, we tested the effects of WNT pathway inhibitors on the expression of NOTCH ligands and cell fate decisions during neuroectoderm differentiation. Blocking canonical WNT signaling with IWR caused an increase in NOTCH ligands DLL1 and DLL2 and NOTCH target gene HES5 expression (Figures 4D, S7A, and S7B), accompanied by an increase in neuroepithelial markers and a decrease in neural crest markers (Figures 4D, S7A, and S7B). The opposite results were obtained using purified WNT4 and WNT8A proteins (Figure S7B) or by inhibiting the NOTCH signaling pathway using the gamma-secretase inhibitor DAPT (Figure S7C). Activation of NOTCH signaling by coupling DLL1 to agarose beads increased neuroepithelial specification at the expense of neural crest formation, while the addition of NOTCH inhibitor DAPT blocked this effect (Figures S7D and S7E). However, NOTCH signaling is insufficient to promote neuroepithelial fate over the neural crest since the simultaneous activation of NOTCH and WNT signaling still induces neural crest

Figure 4. RBL2 directs tissue formation through changing the cellular microenvironment

(A) Genomic regions of DLL1 and DLL3 loci showing E2F1, E2F4, β-catenin ChIP-seq binding with hESC and neuroectoderm (NE) ATAC-seq.

(B) β-Catenin binds to promoters of Notch ligands DLL1 and DLL3 at day 4 neuroectoderm.

(C) WNT/β-catenin regulates the expression of DLL1 and DLL3 indirectly through repressing the activity of the WNT-β-catenin pathway.

(D) WNT signaling inhibition with IWR inhibitor decreases the expression of neural crest marker p75 and increases DLL1, DLL3, and neuroepithelial marker SOX1 in day 3 neuroectoderm cells.

(E) Schematic overview for analyzing the paracrine effects of RBL2.

(F) Confirmation of WNT4/WNT8A depletion from the medium.

(G) RBL2 regulates the amounts of WNT4 and WNT8A secreted into the extracellular milieu. Conditioned medium collected from Scramble or RBL2-KD cells with/without WNT4/WNT8A depletion was added for 4 h to cells previously transfected with a β-catenin-responsive luciferase construct.

(H) RBL2 controls cell fate decisions through paracrine effects of WNT ligands. Conditioned medium from Scramble, RBL2-KD cells, or RBL2-KD cells depleted from WNT4 and WNT8A was added to day 3 neuroectoderm cells and incubated for 24 h.

(I) RBL2 controls cell fate decisions during neuroepithelium versus neural crest formation through paracrine effects of WNT ligands. Scale bar, 100 μm.

(J) WNT4 and WNT8A depletion from medium blocks the effects of RBL2 on NOTCH ligands. Medium from RBL2-KD and/or RBL2-OE cells was placed on cells expressing DLL1 and DLL3 promoter constructs.

(K) RBL2 regulates cell motility. Transwell assays on neuroectoderm cells incubated with medium from RBL2-KD, RBL2-OE, and RBL2-KD + IWR.

(L) Schematic overview of RBL2 function in regulating tissue microenvironment. All data are shown as mean ± SD (n = 3). Statistical analysis was performed by two-way ANOVA with multiple comparisons with Tukey correction; ****$p_{adjusted} < 0.0001$, ***$p_{adjusted} < 0.001$, **$p_{adjusted} < 0.01$, *$p_{adjusted} < 0.05$. See also Figures S6 and S7.

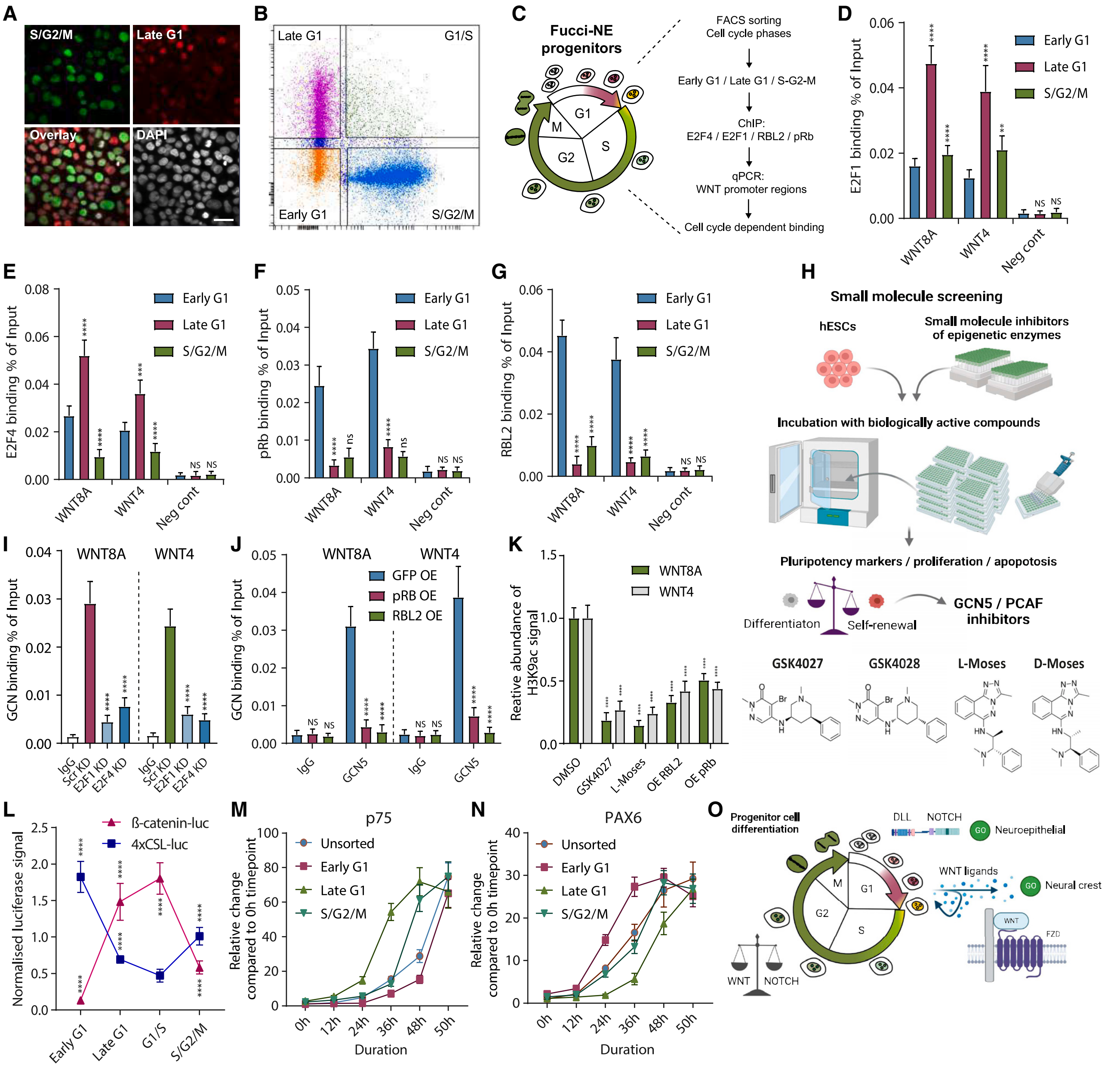

Figure 5. E2Fs-GCN5 induce WNT ligands in late G1 while E2Fs-RBs repress WNTs in early G1 to guide neural crest versus neuroepithelium specification

(A) Representative fluorescence microscopy images of FUCCI-expressing cells. Scale bar, 10 μm.

(B) Dot plot image with gates for sorting FUCCI-expressing cells.

(C) Schematics of cell sorting of FUCCI-neuroectoderm progenitor cells.

(D–G) Cells sorted into early G1, late G1, and S/G2/M phases based on FUCCI signals followed by ChIP-qPCR of E2F1 (D), E2F4 (E), pRb (F), or RBL2 (G) on WNT8A locus or WNT4 locus and a negative control region.

(H) Schematic of small-molecule compound library screening targeting epigenetic regulatory enzymes that identified GCN5/PCAF inhibitors GSK4027 and L-Moses.

(I) GCN5 binding to WNT8A and WNT4 loci is impacted by E2F1/4 KDs analyzed by ChIP-qPCR.

(J) GCN5 binding to WNT8A and WNT4 loci in neuroectoderm progenitor cells is reduced by pRb-OE and RBL2-OE.

(K) GCN5 inhibitors, RBL2-OE and pRb-OE, reduce H3K9ac abundance on WNT8A and WNT4 promoter regions.

(L) Cell-cycle phase-dependent fluctuation of β-catenin and NOTCH activity. FUCCI-neuroectoderm progenitor cells were transfected with TOP Flash and 4xCSL-luc constructs, FACS sorted based on FUCCI signal, and analyzed by luminometer by assaying luciferase activity.

(legend continued on next page)

formation (Figure S7F), suggesting a role for both pathways in differentiation.

Collectively, these results indicate that RBL2 controls the activity of WNT signaling during neuroectoderm differentiation, which in turn controls the expression of NOTCH pathway ligands and ultimately, in parallel to NOTCH signaling, regulates the specification of neural crest versus neuroepithelium (Figure S7G).

RBL2 directs tissue development through paracrine mechanisms

The results suggest that RBL2 exerts a non-cell-autonomous effect on cell fate choice. However, RBL2 could also have a cell-autonomous function involving additional mechanisms. To challenge this hypothesis, we collected the supernatant of neuroectoderm Scramble or RBL2-KD cells and then used the resulting conditioned medium to differentiate a fresh batch of hESCs into neuroectoderm (Figure 4E). Moreover, to specifically test the importance of WNT4 and WNT8A ligands, we depleted these factors from conditioned medium by immunoprecipitation (Figures 4E and 4F). Conditioned medium from RBL2-KD cells resulted in a stronger induction of β-catenin-dependent luciferase activity, while depletion of WNT4/WNT8A considerably diminished this effect (Figure 4G) and resulted in a decrease in the expression of known β-catenin target genes (Figure S7H), thereby confirming that these growth factors mediate the activation of the WNT pathway. Gene expression analysis showed that conditioned medium from RBL2-KD increased the expression of neural crest markers (p75, SOX10), while expression of neuroectoderm markers (SOX1 and PAX6) and NOTCH ligands (DLL1 and DLL3) were downregulated when compared with conditioned medium from Scramble cells (Figures 4H and 4I). Interestingly, these effects were attenuated by depletion of WNT4 and WNT8A from the medium (Figure 4H). The conditioned medium from RBL2-KD cells decreased the transcriptional activity of DLL1 and DLL3 in the NOTCH pathway, while WNT4/WNT8A depletion abolished this effect (Figure 4J). Finally, conditioned medium from RBL2-KD cells increases the migration of neuroectoderm cells compared with medium collected from Scramble KD cells, whereas RBL2 OE had the opposite effect similarly to WNT pathway inhibition with IWR (Figure 4K). The migration capacity is a characteristic of neural crest cells.

Taken together, these data suggest that RBL2 could regulate cell fate decisions of neuroectoderm progenitors in part by controlling the balance of developmental signaling molecules in the extracellular microenvironment (Figure 4L).

E2Fs-GCN5 induce WNT ligands in late G1 while E2Fs-RBs repress WNTs in early G1 to guide neural crest versus neuroepithelium specification in progenitor cells

Our past research has indicated the connection between cell fate decisions and the cell cycle in hESCs,[37] and hence, we hypothesized that the regulation of cell fate decisions in neuroectoderm progenitors specific to neural crest and neuroepithelial routes has a cell-cycle-dependent mechanism. We utilized the Fluorescent Ubiquitination-based Cell Cycle Indicator (FUCCI) system,[37,39] in the context of neuroectoderm differentiation in 3D organoid conditions (Figures 5A–5C). We differentiated FUCCI-hESCs to neuroectoderm progenitors for 4 days and sorted the cells to early G1, late G1, and S/G2/M phases, which was followed by ChIP-qPCR analyses of E2F1/4 (Figures 5D and 5E) and pRb/RBL2 (Figures 5F and 5G) on *WNT8A* and *WNT4* loci. E2F1 and E2F4 bound to WNT8A and WNT4 loci irrespective of the cell-cycle phase, although the enrichment was higher in late G1 phase cells compared with early G1 and S/G2/M phases (Figures 5D and 5E). In contrast, RBL2 and pRb showed strongest binding in early G1 phase, which was particularly reduced in late G1 phase (Figures 5F and 5G).

Next, we performed a small-molecule compound screening in H9 OCT4-GFP cells to identify epigenetic regulators, histone, or DNA modifying enzymes that could impact the pluripotency and differentiation of hESCs by measuring the expression pluripotency markers OCT4, SSEA4, and CD133/PROM1 the cell population (Figure 5H). In the screening, we used a library of 142 small-molecule compounds with verified specificity and biological activity to a broad range of epigenetic modifying enzymes (see supplemental information). For the screening, we plated H9 hESCs into 96-well plates, treated the cells for 5 days with each individual small-molecule compound, and then measured pluripotency marker OCT4, CD133/PROM1, and SSEA4 expression, cell numbers, and cell death by DAPI signal via flow cytometry (Figure 5H). Hence, the screening allows for the detection of differential effects on the subpopulations of pluripotent hESCs and differentiated cells.

From these analyses, we identified GCN5 inhibitors GSK4027 and L-Moses as effective compounds that reduced the percentage of OCT4+/CD133+/SSEA4+ cells, while the corresponding inactive negative control compounds GSK4028 and D-Moses did not have this effect (Figure 5H). GCN5 is the shared catalytic subunit of the ATAC and SAGA complexes that regulate histone acetylation of regulatory regions on chromatin that regulates gene transcription.[73–75] Therefore, the loss of pluripotency marker-expressing cells compared upon GCN5/PCAF inhibition could indicate the possible cooperation of GCN5 with E2Fs also during neuroectoderm differentiation. To test this hypothesis, we performed GCN5 ChIP-qPCR in neuroectoderm cells and found that while GCN5 binds to the same regions on WNT8A and WNT4 loci as E2Fs and RBs, the knockdown of E2F1 and E2F4 reduces the binding of GCN5 to these regulator regions (Figure 5I). Hence, E2F1 and E2F4 bind and recruit GCN5 to WNT8A and WNT4 regulatory regions. In turn, GCN5 binding was reduced upon the overexpression of RBL2 and pRb, indicating a competitive binding of RBs and GCN5 to WNT8A and WNT4 loci (Figure 5J). The

(M and N) The cell cycle regulates the initiation of neuroepithelial and neural crest specification by a temporal separation of signaling activities.

(O) Schematic depiction of cell-cycle-dependent activity of DLL/NOTCH and WNT/β-catenin signaling in neuroectoderm progenitors, which can create cell-cycle-dependent field effects through secreted WNTs and spatiotemporal effects on neuroepithelial versus neural crest specification. All data are shown as mean ± SD (n = 3). Statistical analysis was performed by two-way ANOVA with multiple comparisons with Tukey correction; ****$p_{adjusted} < 0.0001$, ***$p_{adjusted} < 0.001$, **$p_{adjusted} < 0.01$, *$p_{adjusted} < 0.05$.

overexpression of RBL2 and pRb decreased the relative abundance of H3K9ac on WNT8A and WNT4 loci similarly to GCN5 inhibitors GSK4027 and L-Moses (Figure 5K).

Next, we investigated the activity of WNT/β-catenin and DLL/NOTCH signaling pathways by using the Top Flash luciferase and the 4xCSL-luciferase constructs. For this, we transfected the FUCCI-neuroectoderm cells with each DNA construct together with a constitutively active control Renilla luciferase construct, and after FACS, we measured the luciferase signals (Figure 5L). We uncovered a cell-cycle-dependent activity for β-catenin-dependent transcription and NOTCH-dependent transcription, whereas WNT/β-catenin signaling is most active in late G1 and G1/S transition and DLL/NOTCH signaling in early G1 phase (Figure 5L). Lastly, we investigated the propensity of day 2 neuroectoderm cells to initiate the expression of neuroepithelial and neural crest genes. We sorted live FUCCI-neuroectoderm day 2 progenitor cells to distinct cell-cycle phases and tracked the induction of p75 and PAX6 gene expression at different time points. These results indicated that cells in the late G1 phase are particularly efficient and rapid in inducing p75 expression, whereas cells starting at other cell-cycle phases are lagging behind in p75 induction, particularly for early G1 phase cells (Figure 5M). On the other hand, cells starting at early G1 phase are particularly efficient in rapidly inducing PAX6 expression compared with other cell-cycle phases (Figure 5N). These results indicate that the RB-E2F axis mediates temporal fluctuations of WNT/β-catenin and DLL/NOTCH signaling activity during the cell cycle of neuroectoderm progenitors, which is involved in directing cell fate decisions.

Taken together, we have identified a function for the RB-E2F bona fide cell-autonomous cell-cycle regulatory axis in cell fate decisions by showing that E2F-RB-GCN5 circuitry is a regulator of field effects via WNT ligands that can impact tissue formation. This non-cell-autonomous function reveals an unanticipated role of RB-E2F tumor suppressor axis in stem cell and tissue progenitor differentiation that has broader implications for cell fate specification in organogenesis, adult stem cells, and tissue homeostasis.

DISCUSSION

Our data have identified a function for the tumor suppressor RBL2 in regulating cell fate decisions during PSC differentiation. This involves the creation of a specific extracellular signaling environment through balancing the expression of cell-cell signaling molecules of the WNT and NOTCH ligand families.

The paracrine regulation of tissue formation by RBL2 demonstrates that RBL2 is not simply regulating the cell cycle as a tumor suppressor in a cell-autonomous manner but could also have a function in regulating the extracellular niche of stem cells and progenitors. The control of extracellular signals by RBs is directly relevant for embryonic development since RBL2 knockout mice exhibit abnormal neuronal patterning characterized by diminished numbers of neurons in the spinal cord and the dorsal root ganglia.[24] However, these effects seem to depend on the mouse strain and are present in Balbc,[24] but not observed in some other mouse strains.[27–30] It is possible that RB genes have partial functional overlap *in vivo*, as both complexes between E2F/pRb and E2F/p130 have been shown to form during neuronal differentiation,[76] suggesting their role in neuronal development or maintenance of terminal differentiation, while pRb and p107 seem to inhibit E2F activity during lens fiber cell differentiation.[77] Furthermore, WNT signaling has been implicated in neuronal differentiation,[78–80] while WNT4 regulation has been previously connected to E2F1.[81,82] Nonetheless, the molecular mechanisms that have been identified so far have primarily focused on the direct regulation of key transcription factors directing neurogenesis such as Dlx1/2.[83]

E2F4 is usually described as a transcriptional repressor,[84,85] but in our experiments, it had an inductive effect of WNT ligands, while it switches to a repressor function upon the cooperation with RBs. E2F4 has recently been shown to be important for the proliferation and the survival of mouse embryonic stem cells where E2F4 acts in part as a transcriptional activator that promotes the expression of cell-cycle genes and other loci by cooperating with histone acetyltransferases.[86] In our screening experiment for discovering epigenetic regulatory enzymes that would control pluripotency, we identified GCN5, known as a subunit of the ATAC complex that regulates histone acetylation.[73–75] We further showed that it cooperates with E2Fs on *WNT8A* and *WNT4* loci by regulating H3K9 acetylation, thus indicating a mechanism where E2Fs bind to either GCN and RBs in a cell-cycle-dependent manner, as shown by using the FUCCI system in neuroectodermal progenitor cells. In the past we have uncovered a cell-cycle-dependent regulation of cell fate choice in hESCs by using the FUCCI system,[37,39] and our current data provide evidence for a cell-cycle-mediated cell fate decision process in a neuroectoderm, which is a different cellular context. These data indicate that a cell-cycle-mediated initiation of cell fate could be a broadly occurring mechanism in progenitor cells during development but possibly also in adult tissue-specific stem cells.

Our results suggest that developmental anomalies could be provoked by deregulation of morphogen gradients of key signaling pathways such as WNT and NOTCH. This could also be relevant for a diversity of human diseases. Indeed, several genes, which exerted differential gene expression in our microarray due to RBL2 loss, including WNT4, WNT5A, DLL3, and OTX, are known to cause human developmental abnormalities upon deregulation (Table S2). This raises the intriguing possibility that defects in tumor suppressor RBL2 function could affect a diversity of tissues where WNT and NOTCH have key functions. Regarding the functional crosstalk between WNT and NOTCH *in vivo*, these pathways form a dual signaling system that mediates lateral inhibition of boundary cell specification in the zebrafish hindbrain,[87] which has a striking similarity to mechanisms at the dorsoventral boundary in the *Drosophila* wing imaginal disc.[88,89] NOTCH has been shown to inhibit mammalian neuronal differentiation by maintaining neural progenitors[90–92] and functions via HES1 and HES5, which can functionally compensate each other in this process.[93] While blocking neuronal differentiation, NOTCH restricts differentiation to glial differentiation,[90,92,94,95] underlining its importance in controlling tissue formation in the neuroectoderm lineage. NOTCH activation *in vivo* in the mouse embryonic forebrain before neurogenesis promotes radial glial formation, the first specialized cell type

evident in the forebrain, while postnatally NOTCH activity results in the formation of astrocytes.[95] NOTCH seems to crosstalk extensively with WNT *in vivo*, since the WNT pathway can alter the anti-neural activity of NOTCH.[96] Altogether these data underline the evolutionary conservation of the crosstalk of these two important signaling pathways in neuroectoderm development.

Our analyses have so far revealed a strong phenotype only during neuronal differentiation in RBL2-KD hESCs, but RBs could function in diverse developing organs including hematopoietic stem cells, adipocytes, skeletal muscle, and osteoblasts.[18] Functional redundancy between RBs could mask their function during early differentiation. Accordingly, knockout of the three RB genes in mouse ESCs limit their capacity of differentiation[32] while inhibition of pRb activity by overexpression of a mutant form of the SV40 T antigen results in cell death in hESCs. Thus, the different RB proteins can have overlapping and complementary functions.

Finally, tissues *in vivo* contain a variety of stromal cell types including fibroblasts, endothelial cells in the blood and lymphatic circulatory systems, adipocytes and various bone-marrow-derived cells including macrophages, neutrophils, and mesenchymal stem cells, between which there is likely to be crosstalk and therefore an effect on tumor cells via diverse secretory and intercellular factors.[50] Furthermore, the *in vivo* microenvironment and extracellular matrix consists of various other signaling factors including cell adhesion molecules, tight junction proteins, cytokines, and growth factors.[97] It will be interesting to learn how the other components of the tissue microenvironment are affected by RBs during developmental processes, normal adult tissue homeostasis, and tumorigenic processes.

Limitations of the study

The lack of genome-wide binding analyses of RBL2, E2Fs, and GCN5 in different stages of neuroectoderm differentiation would give a broader overview of gene regulation with timing and dynamical mechanisms of repression and induction. This differential regulation is supported by our results on a subset of cell-cycle regulatory genes, WNT ligands, and neuroectoderm genes. The effects of RBL2 on cell patterning could be more extensively studied in 3D organoids to gain insight to the spatiotemporal effects of the cell-autonomous and non-cell-autonomous effects of RBL2 in human ectodermal tissue patterning.

STAR★METHODS

Detailed methods are provided in the online version of this paper and include the following:

- KEY RESOURCES TABLE
- RESOURCE AVAILABILITY
  - Lead contact
  - Materials availability
  - Data and code availability
- EXPERIMENTAL MODEL AND STUDY PARTICIPANT DETAILS
  - Cell lines
- METHOD DETAILS
  - Cell lines and cell culture
  - Differentiation of hPSCs to endoderm, mesoderm and neuroectoderm in 2D conditions
  - 3D neural organoids of hESCs and hiPSCs
  - Generating retinoblastoma family protein knockdown cells
  - CRISPRi mediated knockdown of RBL2
  - Generating RB and RBL2 overexpressing cells
  - RNA isolation and cDNA synthesis
  - Immunostaining
  - qPCR
  - Chromatin immunoprecipitation
  - Transcriptomic analysis
  - Differential expression analysis of transcriptomic data
  - Gene enrichment analysis
  - Principal-component analysis
  - DAPT treatment assay
  - Recombinant proteins
  - DNA constructs
  - Treatment of cells with NOTCH ligand DLL1
  - Depletion of WNT ligands from media
  - Cell incubation with collected media
  - Luciferase assay
  - Transwell assays
  - Western blot analysis
  - Protein co-immunoprecipitation
  - ATAC-sequencing
  - ATAC-sequencing analysis
  - ChIP-sequencing and ATAC-sequencing visualization
  - The small molecule screening library
  - Screening of the chemical compounds
- QUANTIFICATION AND STATISTICAL ANALYSIS

SUPPLEMENTAL INFORMATION

Supplemental information can be found online at https://doi.org/10.1016/j.celrep.2023.113146.

ACKNOWLEDGMENTS

We thank Udo Oppermann, Martin Pook, James Dunford, Martin Philpott, Adam Cribbs, and members of the Pauklin lab for help. We thank Ludovic Vallier and Alessandro Bertero for help and support with the initial stages of the project. This work was supported by the Federation of European Biochemical Societies long-term fellowship and Cancer Research UK Career Development Fellowship grant ID C59392/A25064 to S.P. and the Cambridge Cancer Centre Clinical Research Training Fellowship to M.J.

AUTHOR CONTRIBUTIONS

S.M., R.N., and M.J. performed experiments and provided feedback on the manuscript. S.P. conceived the research, performed the experiments, and wrote the manuscript.

DECLARATION OF INTERESTS

The authors declare no competing interests.

Received: January 15, 2023
Revised: May 30, 2023
Accepted: August 31, 2023
Published: September 19, 2023

REFERENCES

1. Fiorentino, F.P., Symonds, C.E., Macaluso, M., and Giordano, A. (2009). Senescence and p130/Rbl2: a new beginning to the end. Cell Res. *19*, 1044–1051. https://doi.org/10.1038/cr.2009.96.
2. Vélez-Cruz, R., and Johnson, D.G. (2017). The Retinoblastoma (RB) Tumor Suppressor: Pushing Back against Genome Instability on Multiple Fronts. Int. J. Mol. Sci. *18*, 1776. https://doi.org/10.3390/ijms18081776.
3. Indovina, P., Marcelli, E., Casini, N., Rizzo, V., and Giordano, A. (2013). Emerging roles of RB family: new defense mechanisms against tumor progression. J. Cell. Physiol. *228*, 525–535. https://doi.org/10.1002/jcp.24170.
4. Dick, F.A., Goodrich, D.W., Sage, J., and Dyson, N.J. (2018). Non-canonical functions of the RB protein in cancer. Nat. Rev. Cancer *18*, 442–451. https://doi.org/10.1038/s41568-018-0008-5.
5. Lee, E.Y., To, H., Shew, J.Y., Bookstein, R., Scully, P., and Lee, W.H. (1988). Inactivation of the retinoblastoma susceptibility gene in human breast cancers. Science *241*, 218–221. https://doi.org/10.1126/science.3388033.
6. Harbour, J.W., Lai, S.L., Whang-Peng, J., Gazdar, A.F., Minna, J.D., and Kaye, F.J. (1988). Abnormalities in structure and expression of the human retinoblastoma gene in SCLC. Science *241*, 353–357. https://doi.org/10.1126/science.2838909.
7. Horowitz, J.M., Park, S.H., Bogenmann, E., Cheng, J.C., Yandell, D.W., Kaye, F.J., Minna, J.D., Dryja, T.P., and Weinberg, R.A. (1990). Frequent inactivation of the retinoblastoma anti-oncogene is restricted to a subset of human tumor cells. Proc. Natl. Acad. Sci. USA *87*, 2775–2779. https://doi.org/10.1073/pnas.87.7.2775.
8. Horowitz, J.M., Yandell, D.W., Park, S.H., Canning, S., Whyte, P., Buchkovich, K., Harlow, E., Weinberg, R.A., and Dryja, T.P. (1989). Point mutational inactivation of the retinoblastoma antioncogene. Science *243*, 937–940. https://doi.org/10.1126/science.2521957.
9. Marshall, C.J. (1991). Tumor suppressor genes. Cell *64*, 313–326. https://doi.org/10.1016/0092-8674(91)90641-b.
10. Weinberg, R.A. (1991). Tumor suppressor genes. Science *254*, 1138–1146. https://doi.org/10.1126/science.1659741.
11. Xu, H.J., Hu, S.X., Hashimoto, T., Takahashi, R., and Benedict, W.F. (1989). The retinoblastoma susceptibility gene product: a characteristic pattern in normal cells and abnormal expression in malignant cells. Oncogene *4*, 807–812.
12. Jacks, T., Fazeli, A., Schmitt, E.M., Bronson, R.T., Goodell, M.A., and Weinberg, R.A. (1992). Effects of an Rb mutation in the mouse. Nature *359*, 295–300. https://doi.org/10.1038/359295a0.
13. Korenjak, M., and Brehm, A. (2005). E2F-Rb complexes regulating transcription of genes important for differentiation and development. Curr. Opin. Genet. Dev. *15*, 520–527. https://doi.org/10.1016/j.gde.2005.07.001.
14. Lee, E.Y., Chang, C.Y., Hu, N., Wang, Y.C., Lai, C.C., Herrup, K., Lee, W.H., and Bradley, A. (1992). Mice deficient for Rb are nonviable and show defects in neurogenesis and haematopoiesis. Nature *359*, 288–294. https://doi.org/10.1038/359288a0.
15. Clarke, A.R., Maandag, E.R., van Roon, M., van der Lugt, N.M., van der Valk, M., Hooper, M.L., Berns, A., and te Riele, H. (1992). Requirement for a functional Rb-1 gene in murine development. Nature *359*, 328–330. https://doi.org/10.1038/359328a0.
16. Nikitin, A., and Lee, W.H. (1996). Early loss of the retinoblastoma gene is associated with impaired growth inhibitory innervation during melanotroph carcinogenesis in Rb+/- mice. Genes Dev. *10*, 1870–1879. https://doi.org/10.1101/gad.10.15.1870.
17. Zacksenhaus, E., Jiang, Z., Chung, D., Marth, J.D., Phillips, R.A., and Gallie, B.L. (1996). pRb controls proliferation, differentiation, and death of skeletal muscle cells and other lineages during embryogenesis. Genes Dev. *10*, 3051–3064. https://doi.org/10.1101/gad.10.23.3051.
18. Calo, E., Quintero-Estades, J.A., Danielian, P.S., Nedelcu, S., Berman, S.D., and Lees, J.A. (2010). Rb regulates fate choice and lineage commitment in vivo. Nature *466*, 1110–1114. https://doi.org/10.1038/nature09264.
19. Thomas, D.M., Carty, S.A., Piscopo, D.M., Lee, J.S., Wang, W.F., Forrester, W.C., and Hinds, P.W. (2001). The retinoblastoma protein acts as a transcriptional coactivator required for osteogenic differentiation. Mol. Cell *8*, 303–316.
20. Fajas, L., Egler, V., Reiter, R., Hansen, J., Kristiansen, K., Debril, M.B., Miard, S., and Auwerx, J. (2002). The retinoblastoma-histone deacetylase 3 complex inhibits IZIgamma and adipocyte differentiation. Dev. Cell *3*, 903–910.
21. Fajas, L., Landsberg, R.L., Huss-Garcia, Y., Sardet, C., Lees, J.A., and Auwerx, J. (2002). E2Fs regulate adipocyte differentiation. Dev. Cell *3*, 39–49.
22. Rajabi, H.N., Takahashi, C., and Ewen, M.E. (2014). Retinoblastoma protein and MyoD function together to effect the repression of Fra-1 and in turn cyclin D1 during terminal cell cycle arrest associated with myogenesis. J. Biol. Chem. *289*, 23417–23427. https://doi.org/10.1074/jbc.M113.532572.
23. Gu, W., Schneider, J.W., Condorelli, G., Kaushal, S., Mahdavi, V., and Nadal-Ginard, B. (1993). Interaction of myogenic factors and the retinoblastoma protein mediates muscle cell commitment and differentiation. Cell *72*, 309–324. https://doi.org/10.1016/0092-8674(93)90110-c.
24. LeCouter, J.E., Kablar, B., Whyte, P.F., Ying, C., and Rudnicki, M.A. (1998). Strain-dependent embryonic lethality in mice lacking the retinoblastoma-related p130 gene. Development *125*, 4669–4679. https://doi.org/10.1242/dev.125.23.4669.
25. LeCouter, J.E., Kablar, B., Hardy, W.R., Ying, C., Megeney, L.A., May, L.L., and Rudnicki, M.A. (1998). Strain-dependent myeloid hyperplasia, growth deficiency, and accelerated cell cycle in mice lacking the Rb-related p107 gene. Mol. Cell Biol. *18*, 7455–7465.
26. Brunet, T., Radivojkov-Blagojevic, M., Lichtner, P., Kraus, V., Meitinger, T., and Wagner, M. (2020). Biallelic loss-of-function variants in RBL2 in siblings with a neurodevelopmental disorder. Ann. Clin. Transl. Neurol. *7*, 390–396. https://doi.org/10.1002/acn3.50992.
27. Cobrinik, D., Lee, M.H., Hannon, G., Mulligan, G., Bronson, R.T., Dyson, N., Harlow, E., Beach, D., Weinberg, R.A., and Jacks, T. (1996). Shared role of the pRB-related p130 and p107 proteins in limb development. Genes Dev. *10*, 1633–1644. https://doi.org/10.1101/gad.10.13.1633.
28. Herrera, R.E., Sah, V.P., Williams, B.O., Mäkelä, T.P., Weinberg, R.A., and Jacks, T. (1996). Altered cell cycle kinetics, gene expression, and G1 restriction point regulation in Rb-deficient fibroblasts. Mol. Cell Biol. *16*, 2402–2407. https://doi.org/10.1128/MCB.16.5.2402.
29. Hurford, R.K., Jr., Cobrinik, D., Lee, M.H., and Dyson, N. (1997). pRB and p107/p130 are required for the regulated expression of different sets of E2F responsive genes. Genes Dev. *11*, 1447–1463. https://doi.org/10.1101/gad.11.11.1447.
30. Lee, M.H., Williams, B.O., Mulligan, G., Mukai, S., Bronson, R.T., Dyson, N., Harlow, E., and Jacks, T. (1996). Targeted disruption of p107: functional overlap between p107 and Rb. Genes Dev. *10*, 1621–1632. https://doi.org/10.1101/gad.10.13.1621.
31. Conklin, J.F., Baker, J., and Sage, J. (2012). The RB family is required for the self-renewal and survival of human embryonic stem cells. Nat. Commun. *3*, 1244. https://doi.org/10.1038/ncomms2254.
32. Sage, J., Mulligan, G.J., Attardi, L.D., Miller, A., Chen, S., Williams, B., Theodorou, E., and Jacks, T. (2000). Targeted disruption of the three Rb-related genes leads to loss of G(1) control and immortalization. Genes Dev. *14*, 3037–3050.
33. Dannenberg, J.H., van Rossum, A., Schuijff, L., and te Riele, H. (2000). Ablation of the retinoblastoma gene family deregulates G(1) control causing immortalization and increased cell turnover under growth-restricting conditions. Genes Dev. *14*, 3051–3064.

34. Goding, C.R., Pei, D., and Lu, X. (2014). Cancer: pathological nuclear reprogramming? Nat. Rev. Cancer *14*, 568–573. https://doi.org/10.1038/nrc3781.

35. Kareta, M.S., Gorges, L.L., Hafeez, S., Benayoun, B.A., Marro, S., Zmoos, A.F., Cecchini, M.J., Spacek, D., Batista, L.F.Z., O'Brien, M., et al. (2015). Inhibition of pluripotency networks by the Rb tumor suppressor restricts reprogramming and tumorigenesis. Cell Stem Cell *16*, 39–50. https://doi.org/10.1016/j.stem.2014.10.019.

36. Thomson, J.A., Itskovitz-Eldor, J., Shapiro, S.S., Waknitz, M.A., Swiergiel, J.J., Marshall, V.S., and Jones, J.M. (1998). Embryonic stem cell lines derived from human blastocysts. Science *282*, 1145–1147.

37. Pauklin, S., and Vallier, L. (2013). The cell-cycle state of stem cells determines cell fate propensity. Cell *155*, 135–147. https://doi.org/10.1016/j.cell.2013.08.031.

38. Tchieu, J., Zimmer, B., Fattahi, F., Amin, S., Zeltner, N., Chen, S., and Studer, L. (2017). A Modular Platform for Differentiation of Human PSCs into All Major Ectodermal Lineages. Cell Stem Cell *21*, 399–410.e7. https://doi.org/10.1016/j.stem.2017.08.015.

39. Pauklin, S., Madrigal, P., Bertero, A., and Vallier, L. (2016). Initiation of stem cell differentiation involves cell cycle-dependent regulation of developmental genes by Cyclin D. Genes Dev. *30*, 421–433. https://doi.org/10.1101/gad.271452.115.

40. Bertero, A., Madrigal, P., Galli, A., Hubner, N.C., Moreno, I., Burks, D., Brown, S., Pedersen, R.A., Gaffney, D., Mendjan, S., et al. (2015). Activin/nodal signaling and NANOG orchestrate human embryonic stem cell fate decisions by controlling the H3K4me3 chromatin mark. Genes Dev. *29*, 702–717. https://doi.org/10.1101/gad.255984.114.

41. Guy, B., Zhang, J.S., Duncan, L.H., and Johnston, R.J., Jr. (2021). Human neural organoids: Models for developmental neurobiology and disease. Dev. Biol. *478*, 102–121. https://doi.org/10.1016/j.ydbio.2021.06.012.

42. Benito-Kwiecinski, S., and Lancaster, M.A. (2020). Brain Organoids: Human Neurodevelopment in a Dish. Cold Spring Harb. Perspect. Biol. *12*, a035709. https://doi.org/10.1101/cshperspect.a035709.

43. Philippidou, P., and Dasen, J.S. (2013). Hox genes: choreographers in neural development, architects of circuit organization. Neuron *80*, 12–34. https://doi.org/10.1016/j.neuron.2013.09.020.

44. Hayward, P., Kalmar, T., and Arias, A.M. (2008). Wnt/Notch signalling and information processing during development. Development *135*, 411–424. https://doi.org/10.1242/dev.000505.

45. Menendez, L., Yatskievych, T.A., Antin, P.B., and Dalton, S. (2011). Wnt signaling and a Smad pathway blockade direct the differentiation of human pluripotent stem cells to multipotent neural crest cells. Proc. Natl. Acad. Sci. USA *108*, 19240–19245. https://doi.org/10.1073/pnas.1113746108.

46. Chambers, S.M., Fasano, C.A., Papapetrou, E.P., Tomishima, M., Sadelain, M., and Studer, L. (2009). Highly efficient neural conversion of human ES and iPS cells by dual inhibition of SMAD signaling. Nat. Biotechnol. *27*, 275–280. https://doi.org/10.1038/nbt.1529.

47. Betancur, P., Bronner-Fraser, M., and Sauka-Spengler, T. (2010). Assembling neural crest regulatory circuits into a gene regulatory network. Annu. Rev. Cell Dev. Biol. *26*, 581–603. https://doi.org/10.1146/annurev.cellbio.042308.113245.

48. Lai, X., Liu, J., Zou, Z., Wang, Y., Wang, Y., Liu, X., Huang, W., Ma, Y., Chen, Q., Li, F., et al. (2021). SOX10 ablation severely impairs the generation of postmigratory neural crest from human pluripotent stem cells. Cell Death Dis. *12*, 814. https://doi.org/10.1038/s41419-021-04099-4.

49. Theveneau, E., and Mayor, R. (2012). Neural crest delamination and migration: from epithelium-to-mesenchyme transition to collective cell migration. Dev. Biol. *366*, 34–54. https://doi.org/10.1016/j.ydbio.2011.12.041.

50. Polyak, K., and Weinberg, R.A. (2009). Transitions between epithelial and mesenchymal states: acquisition of malignant and stem cell traits. Nat. Rev. Cancer *9*, 265–273. https://doi.org/10.1038/nrc2620.

51. Mandegar, M.A., Huebsch, N., Frolov, E.B., Shin, E., Truong, A., Olvera, M.P., Chan, A.H., Miyaoka, Y., Holmes, K., Spencer, C.I., et al. (2016). CRISPR Interference Efficiently Induces Specific and Reversible Gene Silencing in Human iPSCs. Cell Stem Cell *18*, 541–553. https://doi.org/10.1016/j.stem.2016.01.022.

52. Donato, E., Croci, O., Sabò, A., Muller, H., Morelli, M.J., Pelizzola, M., and Campaner, S. (2017). Compensatory RNA polymerase 2 loading determines the efficacy and transcriptional selectivity of JQ1 in Myc-driven tumors. Leukemia *31*, 479–490. https://doi.org/10.1038/leu.2016.182.

53. Cao, A.R., Rabinovich, R., Xu, M., Xu, X., Jin, V.X., and Farnham, P.J. (2011). Genome-wide analysis of transcription factor E2F1 mutant proteins reveals that N- and C-terminal protein interaction domains do not participate in targeting E2F1 to the human genome. J. Biol. Chem. *286*, 11985–11996. https://doi.org/10.1074/jbc.M110.217158.

54. Lo, K.A., Bauchmann, M.K., Baumann, A.P., Donahue, C.J., Thiede, M.A., Hayes, L.S., des Etages, S.A.G., and Fraenkel, E. (2011). Genome-wide profiling of H3K56 acetylation and transcription factor binding sites in human adipocytes. PLoS One *6*, e19778. https://doi.org/10.1371/journal.pone.0019778.

55. Chicas, A., Wang, X., Zhang, C., McCurrach, M., Zhao, Z., Mert, O., Dickins, R.A., Narita, M., Zhang, M., and Lowe, S.W. (2010). Dissecting the unique role of the retinoblastoma tumor suppressor during cellular senescence. Cancer Cell *17*, 376–387. https://doi.org/10.1016/j.ccr.2010.01.023.

56. Helin, K., Wu, C.L., Fattaey, A.R., Lees, J.A., Dynlacht, B.D., Ngwu, C., and Harlow, E. (1993). Heterodimerization of the transcription factors E2F-1 and DP-1 leads to cooperative trans-activation. Genes Dev. *7*, 1850–1861.

57. Sellers, W.R., Novitch, B.G., Miyake, S., Heith, A., Otterson, G.A., Kaye, F.J., Lassar, A.B., and Kaelin, W.G., Jr. (1998). Stable binding to E2F is not required for the retinoblastoma protein to activate transcription, promote differentiation, and suppress tumor cell growth. Genes Dev. *12*, 95–106. https://doi.org/10.1101/gad.12.1.95.

58. Chen, P.L., Riley, D.J., Chen, Y., and Lee, W.H. (1996). Retinoblastoma protein positively regulates terminal adipocyte differentiation through direct interaction with C/EBPs. Genes Dev. *10*, 2794–2804. https://doi.org/10.1101/gad.10.21.2794.

59. Chen, P.L., Riley, D.J., Chen-Kiang, S., and Lee, W.H. (1996). Retinoblastoma protein directly interacts with and activates the transcription factor NF-IL6. Proc. Natl. Acad. Sci. USA *93*, 465–469. https://doi.org/10.1073/pnas.93.1.465.

60. Novitch, B.G., Mulligan, G.J., Jacks, T., and Lassar, A.B. (1996). Skeletal muscle cells lacking the retinoblastoma protein display defects in muscle gene expression and accumulate in S and G2 phases of the cell cycle. J. Cell Biol. *135*, 441–456. https://doi.org/10.1083/jcb.135.2.441.

61. Adams, P.D., and Kaelin, W.G., Jr. (1995). Transcriptional control by E2F. Semin. Cancer Biol. *6*, 99–108. https://doi.org/10.1006/scbi.1995.0013.

62. Ohtani, K., DeGregori, J., and Nevins, J.R. (1995). Regulation of the cyclin E gene by transcription factor E2F1. Proc. Natl. Acad. Sci. USA *92*, 12146–12150. https://doi.org/10.1073/pnas.92.26.12146.

63. Botz, J., Zerfass-Thome, K., Spitkovsky, D., Delius, H., Vogt, B., Eilers, M., Hatzigeorgiou, A., and Jansen-Dürr, P. (1996). Cell cycle regulation of the murine cyclin E gene depends on an E2F binding site in the promoter. Mol. Cell Biol. *16*, 3401–3409. https://doi.org/10.1128/MCB.16.7.3401.

64. Geng, Y., Eaton, E.N., Picón, M., Roberts, J.M., Lundberg, A.S., Gifford, A., Sardet, C., and Weinberg, R.A. (1996). Regulation of cyclin E transcription by E2Fs and retinoblastoma protein. Oncogene *12*, 1173–1180.

65. Sellers, W.R., Rodgers, J.W., and Kaelin, W.G., Jr. (1995). A potent transrepression domain in the retinoblastoma protein induces a cell cycle arrest when bound to E2F sites. Proc. Natl. Acad. Sci. USA *92*, 11544–11548. https://doi.org/10.1073/pnas.92.25.11544.

66. Adnane, J., Shao, Z., and Robbins, P.D. (1995). The retinoblastoma susceptibility gene product represses transcription when directly bound to the promoter. J. Biol. Chem. *270*, 8837–8843. https://doi.org/10.1074/jbc.270.15.8837.

67. Bremner, R., Cohen, B.L., Sopta, M., Hamel, P.A., Ingles, C.J., Gallie, B.L., and Phillips, R.A. (1995). Direct transcriptional repression by pRB and its reversal by specific cyclins. Mol. Cell Biol. *15*, 3256–3265. https://doi.org/10.1128/MCB.15.6.3256.

68. Weintraub, S.J., Chow, K.N., Luo, R.X., Zhang, S.H., He, S., and Dean, D.C. (1995). Mechanism of active transcriptional repression by the retinoblastoma protein. Nature *375*, 812–815. https://doi.org/10.1038/375812a0.

69. Sellers, W.R., and Kaelin, W.G. (1996). RB [corrected] as a modulator of transcription. Biochim. Biophys. Acta *1288*, M1–M5. https://doi.org/10.1016/0304-419x(96)00014-5.

70. Singh, P., Coe, J., and Hong, W. (1995). A role for retinoblastoma protein in potentiating transcriptional activation by the glucocorticoid receptor. Nature *374*, 562–565. https://doi.org/10.1038/374562a0.

71. Galceran, J., Sustmann, C., Hsu, S.C., Folberth, S., and Grosschedl, R. (2004). LEF1-mediated regulation of Delta-like1 links Wnt and Notch signaling in somitogenesis. Genes Dev. *18*, 2718–2723. https://doi.org/10.1101/gad.1249504.

72. Lobry, C., Oh, P., and Aifantis, I. (2011). Oncogenic and tumor suppressor functions of Notch in cancer: it's NOTCH what you think. J. Exp. Med. *208*, 1931–1935. https://doi.org/10.1084/jem.20111855.

73. Haque, M.E., Jakaria, M., Akther, M., Cho, D.Y., Kim, I.S., and Choi, D.K. (2021). The GCN5: its biological functions and therapeutic potentials. Clin. Sci. *135*, 231–257. https://doi.org/10.1042/CS20200986.

74. Petty, E.L., and Pillus, L. (2021). Cell cycle roles for GCN5 revealed through genetic suppression. Biochim. Biophys. Acta. Gene Regul. Mech. *1864*, 194625. https://doi.org/10.1016/j.bbagrm.2020.194625.

75. Koutelou, E., Farria, A.T., and Dent, S.Y.R. (2021). Complex functions of Gcn5 and Pcaf in development and disease. Biochim. Biophys. Acta. Gene Regul. Mech. *1864*, 194609. https://doi.org/10.1016/j.bbagrm.2020.194609.

76. Corbeil, H.B., Whyte, P., and Branton, P.E. (1995). Characterization of transcription factor E2F complexes during muscle and neuronal differentiation. Oncogene *11*, 909–920.

77. Rampalli, A.M., Gao, C.Y., Chauthaiwale, V.M., and Zelenka, P.S. (1998). pRb and p107 regulate E2F activity during lens fiber cell differentiation. Oncogene *16*, 399–408. https://doi.org/10.1038/sj.onc.1201546.

78. Elizalde, C., Campa, V.M., Caro, M., Schlangen, K., Aransay, A.M., Vivanco, M.d., and Kypta, R.M. (2011). Distinct roles for Wnt-4 and Wnt-11 during retinoic acid-induced neuronal differentiation. Stem Cell. *29*, 141–153. https://doi.org/10.1002/stem.562.

79. Hübner, R., Schmöle, A.C., Liedmann, A., Frech, M.J., Rolfs, A., and Luo, J. (2010). Differentiation of human neural progenitor cells regulated by Wnt-3a. Biochem. Biophys. Res. Commun. *400*, 358–362. https://doi.org/10.1016/j.bbrc.2010.08.066.

80. Lange, C., Mix, E., Rateitschak, K., and Rolfs, A. (2006). Wnt signal pathways and neural stem cell differentiation. Neurodegener. Dis. *3*, 76–86. https://doi.org/10.1159/000092097.

81. Devgan, V., Mammucari, C., Millar, S.E., Brisken, C., and Dotto, G.P. (2005). p21WAF1/Cip1 is a negative transcriptional regulator of Wnt4 expression downstream of Notch1 activation. Genes Dev. *19*, 1485–1495. https://doi.org/10.1101/gad.341405.

82. Suzuki, D.E., Ariza, C.B., Porcionatto, M.A., and Okamoto, O.K. (2011). Upregulation of E2F1 in cerebellar neuroprogenitor cells and cell cycle arrest during postnatal brain development. In vitro cellular & developmental biology. Animal *47*, 492–499. https://doi.org/10.1007/s11626-011-9426-3.

83. Ghanem, N., Andrusiak, M.G., Svoboda, D., Al Lafi, S.M., Julian, L.M., McClellan, K.A., De Repentigny, Y., Kothary, R., Ekker, M., Blais, A., et al. (2012). The Rb/E2F pathway modulates neurogenesis through direct regulation of the Dlx1/Dlx2 bigene cluster. J. Neurosci. *32*, 8219–8230. https://doi.org/10.1523/JNEUROSCI.1344-12.2012.

84. Hsu, J., and Sage, J. (2016). Novel functions for the transcription factor E2F4 in development and disease. Cell Cycle *15*, 3183–3190. https://doi.org/10.1080/15384101.2016.1234551.

85. Dick, F.A., and Rubin, S.M. (2013). Molecular mechanisms underlying RB protein function. Nat. Rev. Mol. Cell Biol. *14*, 297–306. https://doi.org/10.1038/nrm3567.

86. Hsu, J., Arand, J., Chaikovsky, A., Mooney, N.A., Demeter, J., Brison, C.M., Oliverio, R., Vogel, H., Rubin, S.M., Jackson, P.K., and Sage, J. (2019). E2F4 regulates transcriptional activation in mouse embryonic stem cells independently of the RB family. Nat. Commun. *10*, 2939. https://doi.org/10.1038/s41467-019-10901-x.

87. Amoyel, M., Cheng, Y.C., Jiang, Y.J., and Wilkinson, D.G. (2005). Wnt1 regulates neurogenesis and mediates lateral inhibition of boundary cell specification in the zebrafish hindbrain. Development *132*, 775–785. https://doi.org/10.1242/dev.01616.

88. Rulifson, E.J., and Blair, S.S. (1995). Notch regulates wingless expression and is not required for reception of the paracrine wingless signal during wing margin neurogenesis in Drosophila. Development *121*, 2813–2824.

89. Heitzler, P., and Simpson, P. (1991). The choice of cell fate in the epidermis of Drosophila. Cell *64*, 1083–1092.

90. Morrison, S.J., Perez, S.E., Qiao, Z., Verdi, J.M., Hicks, C., Weinmaster, G., and Anderson, D.J. (2000). Transient Notch activation initiates an irreversible switch from neurogenesis to gliogenesis by neural crest stem cells. Cell *101*, 499–510.

91. Dorsky, R.I., Chang, W.S., Rapaport, D.H., and Harris, W.A. (1997). Regulation of neuronal diversity in the Xenopus retina by Delta signalling. Nature *385*, 67–70. https://doi.org/10.1038/385067a0.

92. Furukawa, T., Mukherjee, S., Bao, Z.Z., Morrow, E.M., and Cepko, C.L. (2000). rax, Hes1, and notch1 promote the formation of Muller glia by postnatal retinal progenitor cells. Neuron *26*, 383–394.

93. Ohtsuka, T., Ishibashi, M., Gradwohl, G., Nakanishi, S., Guillemot, F., and Kageyama, R. (1999). Hes1 and Hes5 as notch effectors in mammalian neuronal differentiation. EMBO J. *18*, 2196–2207. https://doi.org/10.1093/emboj/18.8.2196.

94. Tanigaki, K., Nogaki, F., Takahashi, J., Tashiro, K., Kurooka, H., and Honjo, T. (2001). Notch1 and Notch3 instructively restrict bFGF-responsive multipotent neural progenitor cells to an astroglial fate. Neuron *29*, 45–55.

95. Gaiano, N., Nye, J.S., and Fishell, G. (2000). Radial glial identity is promoted by Notch1 signaling in the murine forebrain. Neuron *26*, 395–404.

96. Ramain, P., Khechumian, K., Seugnet, L., Arbogast, N., Ackermann, C., and Heitzler, P. (2001). Novel Notch alleles reveal a Deltex-dependent pathway repressing neural fate. Curr. Biol. *11*, 1729–1738.

97. Joyce, J.A., and Pollard, J.W. (2009). Microenvironmental regulation of metastasis. Nat. Rev. Cancer *9*, 239–252. https://doi.org/10.1038/nrc2618.

98. Pantazis, C.B., Yang, A., Lara, E., McDonough, J.A., Blauwendraat, C., Peng, L., Oguro, H., Kanaujiya, J., Zou, J., Sebesta, D., et al. (2022). A reference human induced pluripotent stem cell line for large-scale collaborative studies. Cell Stem Cell *29*, 1685–1702.e22. https://doi.org/10.1016/j.stem.2022.11.004.

99. Dafinca, R., Scaber, J., Ababneh, N., Lalic, T., Weir, G., Christian, H., Vowles, J., Douglas, A.G.L., Fletcher-Jones, A., Browne, C., et al. (2016). C9orf72 Hexanucleotide Expansions Are Associated with Altered Endoplasmic Reticulum Calcium Homeostasis and Stress Granule Formation in Induced Pluripotent Stem Cell-Derived Neurons from Patients with Amyotrophic Lateral Sclerosis and Frontotemporal Dementia. Stem Cell. *34*, 2063–2078. https://doi.org/10.1002/stem.2388.

CellPress
OPEN ACCESS

100. Fernandes, H.J.R., Hartfield, E.M., Christian, H.C., Emmanoulidou, E., Zheng, Y., Booth, H., Bogetofte, H., Lang, C., Ryan, B.J., Sardi, S.P., et al. (2016). ER Stress and Autophagic Perturbations Lead to Elevated Extracellular alpha-Synuclein in GBA-N370S Parkinson's iPSC-Derived Dopamine Neurons. Stem Cell Rep. *6*, 342–356. https://doi.org/10.1016/j.stemcr.2016.01.013.

101. Hockemeyer, D., Wang, H., Kiani, S., Lai, C.S., Gao, Q., Cassady, J.P., Cost, G.J., Zhang, L., Santiago, Y., Miller, J.C., et al. (2011). Genetic engineering of human pluripotent cells using TALE nucleases. Nat. Biotechnol. *29*, 731–734. https://doi.org/10.1038/nbt.1927.

102. Krentz, N.A.J., Nian, C., and Lynn, F.C. (2014). TALEN/CRISPR-mediated eGFP knock-in add-on at the OCT4 locus does not impact differentiation of human embryonic stem cells towards endoderm. PLoS One *9*, e114275. https://doi.org/10.1371/journal.pone.0114275.

103. Veeman, M.T., Slusarski, D.C., Kaykas, A., Louie, S.H., and Moon, R.T. (2003). Zebrafish prickle, a modulator of noncanonical Wnt/Fz signaling, regulates gastrulation movements. Curr. Biol. *13*, 680–685. https://doi.org/10.1016/s0960-9822(03)00240-9.

104. Saxena, M.T., Schroeter, E.H., Mumm, J.S., and Kopan, R. (2001). Murine notch homologs (N1-4) undergo presenilin-dependent proteolysis. J. Biol. Chem. *13*, 40268–40273. https://doi.org/10.1074/jbc.M107234200.

105. Ramirez, F., Dundar, F., Diehl, S., Gruning, B.A., and Manke, T. (2014). deepTools: a flexible platform for exploring deep-sequencing data. Nucleic Acids Res. *42*, W187–W191.

106. Bolger, A.M., Lohse, M., and Usadel, B. (2014). Trimmomatic: a flexible trimmer for Illumina sequence data. Bioinformatics *30*, 2114–2120. https://doi.org/10.1093/bioinformatics/btu170.

107. Dobin, A., Davis, C.A., Schlesinger, F., Drenkow, J., Zaleski, C., Jha, S., Batut, P., Chiasson, M., and Gingeras, T.R. (2013). STAR: ultrafast universal RNA-seq aligner. Bioinformatics *29*, 15–21.

108. Li, H., and Durbin. (2009). R. Fast and accurate short read alignment with Burrows-Wheeler transform. Bioinformatics *25*, 1754–1760.

109. Quinlan, A.R., and Hall, I.M. (2010). BEDTools: a flexible suite of utilities for comparing genomic features. Bioinformatics *26*, 841–842.

110. Heinz, S., Benner, C., Spann, N., Bertolino, E., Lin, Y.C., Laslo, P., Cheng, J.X., Murre, C., Singh, H., and Glass, C.K. (2010). Simple combinations of lineage-determining transcription factors prime cis-regulatory elements required for macrophage and B cell identities. Mol. Cell *38*, 576–589.

111. Thorvaldsdottir, H., Robinson, J.T., and Mesirov, J.P. (2013). Integrative Genomics Viewer (IGV): high-performance genomics data visualization and exploration. Brief Bioinform. *14*, 178–192.

112. Robinson, M.D., McCarthy, D.J., and Smyth, G.K. (2010). edgeR: a Bioconductor package for differential expression analysis of digital gene expression data. Bioinformatics *26*, 139–140. https://doi.org/10.1093/bioinformatics/btp616.

113. Pantazis CB, Yang A., Lara E, McDonough JA, Blauwendraat C, Peng L, Oguro H, Zou J, Sebesta D, Pratt G, et al A reference induced pluripotent stem cell line for large-scale collaborative studies. Preprint at bioRxiv 10.1101/2021.12.15.472643

114. Vallier, L., Touboul, T., Brown, S., Cho, C., Bilican, B., Alexander, M., Cedervall, J., Chandran, S., Ahrlund-Richter, L., Weber, A., and Pedersen, R.A. (2009). Signaling pathways controlling pluripotency and early cell fate decisions of human induced pluripotent stem cells. Stem Cell. *27*, 2655–2666. https://doi.org/10.1002/stem.199.

115. Lancaster, M.A., Renner, M., Martin, C.A., Wenzel, D., Bicknell, L.S., Hurles, M.E., Homfray, T., Penninger, J.M., Jackson, A.P., and Knoblich, J.A. (2013). Cerebral organoids model human brain development and microcephaly. Nature *501*, 373–379. https://doi.org/10.1038/nature12517.

116. Sutcliffe, M., and Lancaster, M.A. (2019). A Simple Method of Generating 3D Brain Organoids Using Standard Laboratory Equipment. Methods Mol. Biol. *1576*, 1–12. https://doi.org/10.1007/7651_2017_2.

117. Brown, S., Teo, A., Pauklin, S., Hannan, N., Cho, C.H.H., Lim, B., Vardy, L., Dunn, N.R., Trotter, M., Pedersen, R., and Vallier, L. (2011). Activin/Nodal signaling controls divergent transcriptional networks in human embryonic stem cells and in endoderm progenitors. Stem Cell. *29*, 1176–1185. https://doi.org/10.1002/stem.666.

118. Xu, X., Bieda, M., Jin, V.X., Rabinovich, A., Oberley, M.J., Green, R., and Farnham, P.J. (2007). A comprehensive ChIP-chip analysis of E2F1, E2F4, and E2F6 in normal and tumor cells reveals interchangeable roles of E2F family members. Genome Res. *17*, 1550–1561. https://doi.org/10.1101/gr.6783507.

119. Du, P., Kibbe, W.A., and Lin, S.M. (2008). lumi: a pipeline for processing Illumina microarray. Bioinformatics *24*, 1547–1548. https://doi.org/10.1093/bioinformatics/btn224.

120. Smyth, G.K. (2004). Linear models and empirical bayes methods for assessing differential expression in microarray experiments. Stat. Appl. Genet. Mol. Biol. *3*, Article3. https://doi.org/10.2202/1544-6115.1027.

121. Benjamini, Y., and Hochberg, Y. (1995). Controlling the False Discovery Rate: A Practical and Powerful Approach to Multiple Testing. J. R. Stat. Soc. Series B Methodological *57*, 289–300. https://doi.org/10.1111/j.2517-6161.1995.tb02031.x.

122. Huang, D.W., Sherman, B.T., and Lempicki, R.A. (2009). Bioinformatics enrichment tools: paths toward the comprehensive functional analysis of large gene lists. Nucleic Acids Res. *37*, 1–13. https://doi.org/10.1093/nar/gkn923.

123. Huang, D.W., Sherman, B.T., and Lempicki, R.A. (2009). Systematic and integrative analysis of large gene lists using DAVID bioinformatics resources. Nat. Protoc. *4*, 44–57. https://doi.org/10.1038/nprot.2008.211.

124. Vallier, L., Rugg-Gunn, P.J., Bouhon, I.A., Andersson, F.K., Sadler, A.J., and Pedersen, R.A. (2004). Enhancing and diminishing gene function in human embryonic stem cells. Stem Cell. *22*, 2–11. https://doi.org/10.1634/stemcells.22-1-2.

125. Estarás, C., Benner, C., and Jones, K.A. (2015). SMADs and YAP compete to control elongation of beta-catenin:LEF-1-recruited RNAPII during hESC differentiation. Mol. Cell *58*, 780–793. https://doi.org/10.1016/j.molcel.2015.04.001.

126. Valcourt, J.R., Huang, R.E., Kundu, S., Venkatasubramanian, D., Kingston, R.E., and Ramanathan, S. (2021). Modulating mesendoderm competence during human germ layer differentiation. Cell Rep. *37*, 109990. https://doi.org/10.1016/j.celrep.2021.109990.

STAR★METHODS

KEY RESOURCES TABLE

REAGENT or RESOURCE	SOURCE	IDENTIFIER
Antibodies		
Goat anti-human Nanog	R&D Systems	Cat# AF1997
Mouse anti-human Oct4	Santa Cruz Biotechnology	Cat# sc-5279
Goat anti-human SOX2	R&D Systems	Cat# AF2018
Rabbit anti-human Eomes	Abcam	Cat# ab23345
Goat anti-human Brachyury	R&D Systems	Cat# AF2085
Goat anti-human SOX17	R&D Systems	Cat# AF1924
PAX6 rabbit polyclonal	Cambridge BioScience	Cat# PRB-278P-100
pRb mouse monoclonal	BD Pharmingen	Cat# 554136 (G3-245)
RBL1/p107 (C-18) rabbit polyclonal	Santa Cruz Biotechnology	Cat# sc-318
RBL2/p130 (C-20) rabbit polyclonal	Santa Cruz Biotechnology	Cat# sc-317
SOX1 goat polyclonal	R&D Systems	Cat# AF3369
Actin mouse monoclonal	Chemicon	Cat# MAB1501
E2F1 (C-20)	Santa Cruz Biotechnology	Cat# sc-193
E2F4 (A-20)	Santa Cruz Biotechnology	Cat# sc-1082x
SOX1 goat polyclonal	R&D Systems	Cat# AF3369
p75 (C-20) goat polyclonal	Santa Cruz Biotechnology	Cat# sc-6188
WNT4 (m-70) rabbit polyclonal	Santa Cruz Biotechnology	Cat# sc-13962
WNT5A (H-58) rabbit polyclonal	Santa Cruz Biotechnology	Cat# sc 30224
WNT8A rabbit polyclonal	Sigma	Cat# SAB1411397
WNT7B goat polyclonal	R&D Systems	Cat# AF3460
HES5 rabbit polyclonal	Abcam	Cat# ab25374
DLL1 H-265 rabbit polyclonal	Santa Cruz Biotechnology	Cat# sc-9102
DLL3 H-110 rabbit polyclonal	Santa Cruz Biotechnology	Cat# sc-67270
P-ser33-B-cat rabbit polyclonal	Santa Cruz Biotechnology	Cat# sc-16743-R
B-catenin (H-102) rabbit polyclonal	Santa Cruz Biotechnology	Cat# sc-7199
Beta Tubulin 3/Tuj1 [GT1338] mouse monoclonal	Stratech	Cat# GTX631831-GTX
Histone H3	Abcam	Cat# ab1791
Histone H3 (tri methyl K4)	Abcam	Cat# ab8580
Histone H3 (tri methyl K27)	Diagenode	Cat# C15200181 (MAb-181-050)
Histone H3 (mono methyl K4)	Abcam	Cat# ab8895
Histone H3 (acetyl K27)	Active Motif	Cat# 39135
Histone H3 (tri methyl K36)	Abcam	Cat# ab9050
SMAD2/3	Bio-techne	Cat# AF3797
Oct-3/4 (C-10)	Santa Cruz	Cat# sc-5279
Actin, clone C4	Chemicon	Cat# MAB1501
Goat α-mouse IgM Alexa Fluor 647	Invitrogen	Cat# A21238
Donkey α-mouse IgG Alexa Fluor 647	Invitrogen	Cat# A31571
Donkey α-goat Alexa Fluor 647	Invitrogen	Cat# A21447
Mouse Anti-Human CD133-BV786, clone W6B3C1	BD Biosciences	Cat# BD 747640
Mouse anti-SSEA-4 Alexa Fluor 647, clone MC813-70	BD Biosciences	Cat# BD 560796
Mouse IgG1-BV786, k Isotype Control	BD Biosciences	Cat# BD 563330

(*Continued on next page*)

Continued		
REAGENT or RESOURCE	SOURCE	IDENTIFIER
Mouse IgG3 Alexa Fluor 647, k Isotype Control	BD Biosciences	Cat# BD 560803
Goat IgG control	R&D Systems	Cat# AB-108-C
IgG from goat serum	Sigma-Aldrich	Cat# I5256-10MG
IgG from mouse serum	Sigma-Aldrich	Cat# I5381-1MG
IgG from rabbit serum	Sigma-Aldrich	Cat# I5006-10MG
Ki67	Abcam	Cat# ab15580
Chemicals, peptides, and recombinant proteins		
Activin A	Qkine	Cat# QK001
SB431542	Tocris	Cat# 1614
Animal-free FGF-Basic TS	Proteintech	Cat# HZ-1285
Pierce EGS Crosslinker	Thermo Fisher Scientific	Cat# 21565
Formaldehyde solution	Sigma-Aldrich	Cat# F8775
Gemcitabine 10mM/1mL	Selleck Chemicals	Cat# S1714
G418 disulfate salt solution	Sigma-Aldrich	Cat# G8168
Puromycin	Sigma-Aldrich	Cat# P8833
EGS (ethylene glycol bis(succinimidyl succinate))	ThermoFisher	Cat# 21565
Formaldehyde	Millipore	Cat# 104003
Glycine	Sigma-Aldrich	Cat# G8898
dATP	New England BioLabs	Cat# N0440S
T4 DNA Ligase, HC (30 U/μL)	ThermoFisher	Cat# EL0013
T4 DNA Ligase Buffer	ThermoFisher	Cat# 46300018
T4 DNA Ligase Reaction Buffer	New England BioLabs	Cat# B0202S
DNA Polymerase I (E. coli)	New England BioLabs	Cat# M0209L
Dynabeads^{TM} Protein G	ThermoFisher	Cat# 10009D
AMPURE XP beads	Beckman Coulter	Cat# A63881
Dynabeads^{TM} M-280 Streptavidin	ThermoFisher	Cat# 11206D
Proteinase K	Life Technologies	Cat# AM2548
Phenol:chloroform:IAA	ThermoFisher	Cat# AM9730
2% Agarose Gel Cassettes	Sage Science	Cat# BDF2010
Mayers Hematoxylin	Sigma-Aldrich	Cat# MHS16
Alcoholic-Eosin	ThermoFisher	Cat# 71204
WNT4 protein	RnD Systems	6076-Wn
WNT8A protein	Genemed PlexBio	90007–02
WNT7B protein	Abcam	ab289780
Critical commercial assays		
NEBNext rRNA Depletion Kit v2 (Human/ Mouse/Rat)	New England Biolabs	Cat#E7400L
Nebnext Ultra II Directional RNA Library Prep Kit for Illumina	New England Biolabs	Cat# E7760S
NEBNext Ultra II DNA Library Prep Kit for Illumina	New England Biolabs	Cat# E7645L
Nebnext High-Fidelity 2X PCR Master Mix	New England Biolabs	Cat# M0541S
Nebnext NGS DNA Library Preparation for Illumina	New England Biolabs	Cat# E7335S
NEBuilder HiFi DNA Assembly Master Mix	New England Biolabs	Cat# E2621L
Nextera XT Index Kit v2 Set	Illumina	Cat# FC-131-2001
Pierce BCA Protein Assay	Thermo Scientific	Cat# 23228

(*Continued on next page*)

Continued

REAGENT or RESOURCE	SOURCE	IDENTIFIER
Pierce SuperSignal West Pico PLUS	Thermo Scientific	Cat# 34580
QIAGEN Plasmid Maxi Kit	Qiagen	Cat# 12162
QIAprep Spin Miniprep Kit	Qiagen	Cat# 27106
QIAquick PCR Purification Kit	Qiagen	Cat# 28106
Direct-zol RNA Miniprep	Zymo Research	Cat# R2050
Nuclear Complex Co-IP Kit	Active Motif	Cat# 54001
DNA Clean & Concentrator-5	Zymo research	Cat# 4013
Nextera DNA sample preparation kit	Illumina, Inc	Cat# FC-121-1030
Dual-Luciferase® Reporter Assay System	Promega	Cat# E1910
PureLink® RNA Mini kit	Thermo Fisher Scientific	Cat# 12183018A
SuperScript™ the First-Strand Synthesis System for RT-PCR kit	Thermo Fisher Scientific	Cat# 11904018
Power SYBR Green Master Mix	Thermo Fisher Scientific	Cat# 4385616
Deposited data		
Gene expression data	ArrayExpress	Accession number: E-MTAB-3586
Experimental models: Cell lines		
H9 hESCs	WiCell	WiCell Research Institute
KOLF2-C1	Wellcome Sanger Institute	Pantazis et al.[98]
SFC841-03-01	OPDC/StemBANCC	Dafinca et al.[99]
SFC840-03-03	OPDC/StemBANCC	Fernandes et al.[100]
Recombinant DNA		
OCT4-eGFP-PGK-Puro	Addgene	31937; Hockemeyer et al.[101]
pTALEN_V2-OCT4F	Provided by Prof. Francis Lynn, The University of British Columbia	Krentz et al.[102]
pTALEN_V2-OCT4R	Provided by Prof. Francis Lynn, The University of British Columbia	Krentz et al.[102]
pCCC-Oct4 construct	Provided by Prof. Francis Lynn, The University of British Columbia	Krentz et al.[102]
Mission pLKO.1-puro Non-Target shRNA Control Plasmid	Merck	Cat# SHC016-1EA
pRb shRNA constructs	Merck	Cat# SHCLNG NM_00032
RBL1 shRNA constructs	Merck	Cat# SHCLNG NM_002895
RBL2 shRNA constructs	Merck	Cat# SHCLNG NM_005611
pRb OE	Source BioScience Lifesciences	Cat# B0065
RBL2 OE	Source BioScience Lifesciences	Cat# T8278
M50 Super 8x TOPFlash	Veeman et al.[103]	Addgene plasmid # 12456
M51 Super 8x FOPFlash (TOPFlash mutant)	Veeman et al.[103]	Addgene plasmid # 12457
4xCSL-luciferase	Saxena et al.[104]	Addgene plasmid # 41726
pAAVS1-NDi-CRISPRi (Gen2)	Mandegar et al.[51]	Addgene plasmid # 73498
pgRNA-CKB	Mandegar et al.[51]	Addgene plasmid # 73501
Software and algorithms		
ImageJ	NIH	https://imagej.nih.gov/ij/
FlowJo	FLOWJO LLC	https://www.flowjo.com/
STRING	string-db	http://string-db.org/
GraphPad Prism	GraphPad Software Inc.	http://www.graphpad.com/scientific-software/prism/
Mascot, version 2.6.0	Matrix Science	https://www.matrixscience.com/
ImageJ	NIH	https://imagej.nih.gov/ij/
FlowJo	FLOWJO LLC	https://www.flowjo.com/

(*Continued on next page*)

Continued		
REAGENT or RESOURCE	SOURCE	IDENTIFIER
STRING	string-db	http://string-db.org/
deepTools	Ramírez et al.[105]	https://deeptools.readthedocs.io/en/develop/
Picard	N/A	https://broadinstitute.github.io/picard/)
MACS2	N/A	https://github.com/macs3-project/MACS
FastQC	N/A	https://www.bioinformatics.babraham.ac.uk/projects/fastqc/
Trimmomatic	Bolger et al.[106]	http://www.usadellab.org/cms/?page=trimmomatic
STAR	Dobin et al.[107]	https://github.com/alexdobin/STAR
bwa	Li and Durbin[108]	https://github.com/lh3/bwa
bedtools	Quinlan and Hall[109]	https://bedtools.readthedocs.io/en/latest/
Homer findMotifs.pl	Heinz et al.[110]	http://homer.ucsd.edu/homer/motif/
IGV	Thorvaldsdottir et al.[111]	https://software.broadinstitute.org/software/igv/
edgeR	Robinson et al.[112]	https://bioconductor.org/packages/release/bioc/html/edgeR.html
Other		
3X IBLOT2 TRNS STK, PVDF, REG 3X IB24001	Life Technologies	Cat# IB24001X3
iBlot^{TM} 2 Transfer Stacks, PVDF, mini	Thermo Fisher Scientific	Cat# IB24002
Invitrogen Novex NuPAGE MES SDS Running Buffer (20X)	Thermo Fisher Scientific	Cat# NP0002
Invitrogen Novex NuPAGE 4 12% Bis Tris Protein Gels, 1.0mm, 10 well3X IBLOT2 TRNS STK, PVDF, REG 3X IB24001	Thermo Fisher Scientific	Cat# NP0321BOX
Invitrogen novex NuPAGE LDS Sample Buffer (4X) iBlot^{TM} 2 Transfer Stacks, PVDF, mini	Thermo Fisher Scientific	Cat# NP0007
Invitrogen novex NuPAGE MOPS SDS Running Buffer (20X) Invitrogen Novex NuPAGE MES SDS Running Buffer (20X)	Thermo Scientific	Cat# NP0001
Invitrogen SuperScript IV Reverse Transcriptase Invitrogen	Thermo Fisher Scientific	Cat# 18-090-050
Invitrogen T4 DNA Ligase Buffer Invitrogen novex NuPAGE LDS Sample Buffer (4X)	Thermo Fisher Scientific	Cat# 46-300-018
Corning Ultra-Low Attachment 75cm2 Rectangular Canted Neck Cell Culture Flask with Vent	Scientific Laboratory Supplies Limited	Cat# 3814
Corning Costar 6 Well Clear Flat Bottom Ultra Low Attachment Multiple Well Plates Wrapped Sterile	Scientific Laboratory Supplies Limited	Cat# 3471
6-Well Ultra-Low Adherent Plate	STEMCELL Technologies	Cat# 100-0083
Corning 96 Well Clear Flat Bottom Ultra Low Attachment Microplate Wrapped with Lid Sterile	Sigma-Aldrich	Cat# 3474
Corning Primaria Surface Modified Cell Culture Dish, 100 × 20mm (Diam x H), 58.95cm2 Cell Growth Area	Scientific Laboratory Supplies Limited	Cat# 353803
Corning Primaria 6 Well Cell Clear Flat Bottom Surface Modified Culture Plate with Lid Sterile	Scientific Laboratory Supplies Limited	Cat# 353846

(*Continued on next page*)

Continued		
REAGENT or RESOURCE	SOURCE	IDENTIFIER
Corning Primaria 24 Well Cell Clear Flat Bottom Surface Modified Culture Plate with Lid Sterile	Scientific Laboratory Supplies Limited	Cat# 353847
Corning® Primaria^{TM} 96 Well Clear Flat Bottom Microtest Microplate, with Lid, Sterile	Scientific Laboratory Supplies Limited	Cat# 353872
Nunc® Easy Flask^{TM} Non-Treated Culture Flasks, Polystyrene, Sterile, 75 cm2	Thermo Scientific	Cat# 156800
Nunc® Easy Flask^{TM} Non-Treated Culture Flasks, Polystyrene, Sterile, 175 cm2	Thermo Scientific	Cat# 159926
Lipofectamine^{TM} 3000 Transfection Reagent	Thermo Fisher Scientific	Cat# L3000008
Applied Biosystems Power SYBR Green PCR Master Mix	Thermo Fisher Scientific	Cat# 4368706
Power SYBR Green PCR Master Mix-1 x 5 mL	Life Technologies	Cat# 4368702
Agilent High Sensitivity DNA Kit	Agilent Technologies	Cat# 5067-4626
Alui, Size = 5,000 units	New England Biolabs	Cat# R0137L
Essential 8 medium	Thermo Fisher Scientific	Cat# A1517001
B-27^{TM} Supplement (50X), serum free	Thermo Fisher Scientific	Cat# 17504001
COMPLETE EDTA-FREE (20 TABLETS)	Roche	Cat# 11873580001
DMEM, high glucose, GlutaMAX^{TM} Supplement, pyruvate	Life Technologies	Cat# 31966047
Dulbecco's Modified Eagle's Medium/ Nutrient Mixture F-12 HamB-27^{TM} Supplement (50X), serum free	Sigma-Aldrich	Cat# D8437-6X500ML
FETAL BOVINE SERUM HEAT INACTIVATEDCOMPLETE EDTA-FREE (20 TABLETS)	Merck	Cat# F9665-500ML
Fetal Bovine Serum, qualified, heat inactivated, E.U.-approved, South America Origin (500 mL)	Life Technologies	Cat# 10500064
MEM Non-Essential Amino Acids Solution (100X)	Life Technologies	Cat# 11140035
MEM Vitamin Solution (100X)	Life Technologies	Cat# 11120037
Opti-MEM^{TM} I Reduced Serum Medium	Thermo Scientific	Cat# 31985062
Invitrogen Ambion Proteinase K Solution (20 mg/mL)	Thermo Fisher Scientific	Cat# AM2546
TrypLE Express Enzyme (1X), No Phenol Red	Thermo Fisher Scientific	Cat# 12-604-021
METHANOL FOR HPLC $\geq$99.9%	Sigma-Aldrich	Cat# 34860-1L-R
Protein A/G Plus-agarose beads	Santa Cruz Biotechnology	Cat# sc-2003

RESOURCE AVAILABILITY

Lead contact

Further information and requests for resources and reagents should be directed to and will be fulfilled by the lead contact, Siim Pauklin (siim.pauklin@ndorms.ox.ac.uk).

Materials availability

Newly generated materials associated with the paper should be requested by contacting the lead contact.

Data and code availability

- The gene expression data can be accessed through ArrayExpress (E-MTAB-3586). Other data is available upon requests from the lead contact.
- This paper does not report original code.
- Any additional information required to reanalyze the data reported in this work paper is available from the lead contact upon request.

EXPERIMENTAL MODEL AND STUDY PARTICIPANT DETAILS

Cell lines

In this study we used H9 hESCs (H9 from WiCell) and hiPSCs (KOLF2-C1 from Wellcome Sanger Institute[98,113]; OPDC/StemBANCC name SFC841-03-01[99]; OPDC/StemBANCC name SFC840-03-03[100]).

METHOD DETAILS

Cell lines and cell culture

H9 hESCs (H9 from WiCell) and hiPSCs (KOLF2-C1 from Wellcome Sanger Institute[113,98]; OPDC/StemBANCC name SFC841-03-01[99]; OPDC/StemBANCC name SFC840-03-03[100]) were maintained in Essential 8 medium (Thermo Fisher Scientific) and passaged using EDTA.

Differentiation of hPSCs to endoderm, mesoderm and neuroectoderm in 2D conditions

H9 cells were differentiated into endoderm, mesoderm and neuroectoderm as described previously.[114] Cells were cultured in CDM supplemented with SB-431542 (10 μM; Tocris) and FGF2 (12 ng/mL) for neuroectoderm, in CDM+PVA supplemented with Activin A (100 ng/mL), FGF2 (20 ng/mL), BMP4 (10 ng/mL), Ly294002 (10 μM; Promega) and CHIR99021 (3 μM; Selleck) for mesoderm and in CDM-PVA supplemented with Activin A (100 ng/mL), FGF2 (20 ng/mL), BMP4 (10 ng/mL) and Ly294002 (10 μM; Promega) for endoderm. Daily media changes were made during the entire differentiation protocol.

3D neural organoids of hESCs and hiPSCs

Cells were differentiated in cerebral organoid conditions as described previously,[115,116] with minor modifications. On day 0 of organoid culture, hPSCs were treated with EDTA and then Accutase, both for 4 min at 37°C, to generate single cells. To generate Embryoid Bodies (EBs), 9000 cells were plated in each well of an U-bottomed ultra-low binding 96-well plate (Corning) in 150μL of hESC media with FGF2 (4 ng/mL) and 50μM Y-27623 ROCK inhibitor (Calbiochem), and the plate was incubated for 3 days. On day 3 half of the medium from each well was aspirated and 150 μL of fresh hESC medium without bFGF or ROCK inhibitor was added to the wells. On day 6 the EBs (500–600 μm in size) were transferred to 24-well ultra-low attachment plates (Corning) in 500 μL of neural induction (NI) media containing DMEM/F12, 1% (v/v) N2 supplement (Invitrogen), 1% Glutamax (Invitrogen), 1% MEM-NEAA, and 1 μg/ml Heparin (Sigma). These began forming neuroepithelial tissues, which were fed every other day for 5 days. For changing the media, about half of the media from the wells were aspirated and 500 μL of fresh NI medium added. On Day 11 of the protocol, tissues were transferred to droplets of Matrigel (BD Biosciences) by pipetting into cold Matrigel on a sheet of Parafilm with small 3mm dimples. The droplets were allowed to polymerize for 20–30 min at 37°C and were subsequently removed from the Parafilm by gently spraying off the droplets when adding the differentiation media containing a 1:1 mixture of DMEM/F12 and Neurobasal containing 0.5% N2 supplement (Invitrogen), 1% B27 supplement without vitamin A (Invitrogen), 3.5μl/L 2-mercaptoethanol, 2.5 μg/mL insulin (Sigma), 1% GlutaMAX supplement (Invitrogen), 0.5% MEM-NEAA, and 1:100 Penicillin/Streptomycin. The media was changed every other day by tilting the dish to one side and waiting for the tissues to sediment to the other side, and then aspirating the media without touching the neuroepithelia. 5mL of the same fresh media was added to the wells. After 4 days of stationary growth, the tissue droplets were grown on a standard orbital shaker at 85 rpm with media in wells as above except B27 supplement with vitamin A (Invitrogen) was used.

Generating retinoblastoma family protein knockdown cells

For RB single knockdown, previously validated shRNA expression vectors (Sigma-Aldrich, Cat no. SHCLNG NM_00032, SHCLNG NM_002895, SHCLNG NM_005611, directed against pRb, RBL1 or RBL2 respectively, were transfected into H9 hPSCs with lipofectamine 2000[37] and grown for 3 days. Cells were then cultured in the presence of puromycin until antibiotic-resistant colonies appeared. These were picked and characterized for knockdown efficiency. We characterized two knockdown clones generated from separate shRNA constructs in more detail.

CRISPRi mediated knockdown of RBL2

hPSCs were transfected with Dox-inducible CRISPR interference (CRISPRi) knock in construct into the AAVS1 locus. pAAVS1-NDi-CRISPRi (Gen2) was a gift from Bruce Conklin (Addgene plasmid # 73498; http://n2t.net/addgene:73498; RRID:Addgene_73498).[51]

Stable cell lines were differentiated with guide RNA construct pgRNA-CKB with an RBL2 gRNA cloned into the construct. pgRNA-CKB was a gift from Bruce Conklin (Addgene plasmid # 73501; http://n2t.net/addgene:73501; RRID:Addgene_73501).

Generating RB and RBL2 overexpressing cells

For RB and RBL2 overexpression, sequence-validated Gateway attL-flanked entry clones (Source BioScience Lifesciences, Cat no. B0065, T8278, for RB and RBL2 overexpression respectively), were transferred into a Gateway-compatible pTP6 vector containing a CAG promoter. The inserts were confirmed by sequencing. Vectors were transfected into H9 hPSCs by lipofection[37] and grown for 3 days. Thereafter, cells with a stable integration were selected by continuous presence of puromycin. Individual clones were picked, propagated and used for subsequent analyses.

RNA isolation and cDNA synthesis

Total RNA was isolated by RNeasy RNA Extraction Kit (Qiagen) according to manufacturer's guidelines. RNA was then eluted in 30μL of water and the concentration was measured using Nanodrop. The master mix was prepared as follows: 8μL 5x First-Strand Buffer (Invitrogen), 0.5μL Random primers (0.5 μg/mL) (Promega Cat. C1181), 1μl dNTP mix (10 mM each) (Promega Cat.U1515), 2 μL 0.1 M DTT, 0.5 μL RNase Out, 0.25 μL Superscript III Reverse Transcriptase (Life Technologies). 500 ng of total RNA into a separate tube with 11.75 μL RNase-free water. RNA was heated to 65°C for 5 min and allowed to chill on ice for 2 min 8.25 μL of the master mix were added to RNA. The reaction was incubated at 25°C for 10 min and then at 42°C for 50 min. The reaction was then inactivated by heating at 70°C for 15 min.

Immunostaining

Methods for immunostaining have been described previously.[37,39,40,114] Cells were fixed for 20 min at 4°C in PBS 4% PFA (electron microscopy grade), rinsed three times with PBS, and blocked and permeabilized at the same time for 30 min at room temperature using PBS with 10% Donkey Serum (Biorad) and 0.1% Triton X-100 (Sigma). Incubation with primary antibodies diluted in PBS 1% Donkey Serum 0.1% Triton X-100 was performed overnight at 4°C. Samples were washed three times with PBS, and then incubated with AlexaFluor secondary antibodies for 1 h at room temperature protected from light. Cells were finally washed three times with PBS, and Hoechst (Sigma) was added to the first wash to stain nuclei. Images were acquired using an LSM 700 confocal microscope (Leica).

qPCR

Methods for qPCR have been described previously.[37,39,40,114] qPCR data are presented as the mean of three independent experiments and error bars indicate standard deviations. Antibodies and primer sequences have been listed in Table S3

Chromatin immunoprecipitation

hPSCs were washed with PBS and detached from the plate by incubating them for 10 min at 37°C in Cell Dissociation Buffer (GIBCO). ChIP was carried out as described before[39,40,117] with some modifications. The ChIP experiments were performed in triplicate. All steps were performed on ice or at 4°C and ice-cold buffers and PBS were supplemented with 1 mg/ml Leupeptin, 0.2mM PMSF, and 10mM NaButyrate were used unless otherwise stated. Approximately 5x106 cells were used per sample and cross-linked with 1% formaldehyde for 15 min. Cross-linking was stopped by incubating samples with glycine at a final concentration of 0.125M for 5 min at room temperature, and the cells were washed with PBS followed by pelleting at 250g for 5 min. The pellet was re-suspended in 2mL ChIP Cell Lysis Buffer (CLB: 10 mM Tris pH8, 10 mM NaCl, 0.2% NP-40) and incubated for 10 min to lyse the plasma membranes. Nuclei were pelleted at 600g for 5 min, lysed in 1.25mL of ChIP Nuclear Lysis Buffer (NLB: 50 mM Tris pH8, 10mM EDTA, 1% SDS) for 10 min, and then 0.75mL of ChIP Dilution Buffer (DB: 20 mM Tris pH8, 2mM EDTA, 150mM NaCl, 0.01% SDS, 1% Triton X-100) was added to the samples. Chromatin was sonicated in 15mL Diagenode Bioruptor Pico water bath sonicator with an automated water cooling system, by performing 30 cycles of 30 s ON, 45 s OFF. This protocol resulted in the homogeneous generation of fragments of 100-400bp. Samples were clarified by centrifugation at 16000*g* for 10 min, and diluted with 3.5mL of DB. After pre-clearing with 10μg of non-immune IgG for 1h and 50μL of Protein G Agarose for 2h, ChIP was performed overnight in rotation using specific antibodies (Table S3) or non-immune IgG as a control. After incubation for 1 h with 30μL of Protein G Agarose, beads were washed twice with ChIP Washing Buffer 1 (WB1: 20mM Tris pH8, 2mM EDTA, 50mM NaCl, 0.1% SDS, 1% Triton X-100), once with ChIP Washing Buffer 2 (WB2: 10mM Tris pH8, 1mM EDTA, 0.25M LiCl, 1% NP-40, 1% Deoxycholic acid), and twice with Tris-EDTA (TE: 10mM Tris pH8, 1mM EDTA). Precipitated DNA was eluted with 150μL of ChIP Elution Buffer (EB: 100mM NaHCO3) twice for 15 min at room temperature in rotation, and processed as follows in parallel with 300μL of sonicated chromatin non-used for ChIP (Input). Cross-linking was reverted by adding NaCl to a final concentration of 300mM for protein-DNA de-crosslinking and incubated at 65°C for 5 h and 1μg RNase A (Sigma) to digest contaminating RNA. Finally, 60μg of Proteinase K (Sigma) were added overnight at 45°C. DNA was extracted by sequential phenol-chloroform and chloroform extractions, and precipitated overnight at −80°C in 100mM NaAcetate, 66% ethanol and 50μg of glycogen (Ambion) as a carrier. After centrifugation at 16,000g for 1 h at 4°C, DNA pellets were washed once with ice-cold 70% ethanol, and finally air dried. ChIP samples were resuspended in 30μL and 1:10 of the samples were used in qPCR for verifying the ChIP samples.

E2F/RB binding site identification was performed as follows. E2F/Rb proteins usually bind to their target loci within 2kb of the transcription start site.[118] Hence we designed ChIP primers every 250 bp within 2kb upstream to 500bp downstream of transcription start site, and tested them by qPCR after performing ChIP E2F1. These results identified primers that allowed the optimal detection of E2F binding, while primer pairs further away from These regions did not show an enrichment for E2F and RBL2 binding on WNT loci. All ChIP experiments also included negative binding regions on other loci such as Smad7, which did not show any enrichment for E2F/RB binding.

Transcriptomic analysis

500ng of total cellular RNA were amplified and purified using the Illumina TotalPrep-96 RNA Amplification kit (Life Technologies) according to the manufacturer's instructions. Three biological replicates for each condition were analyzed. Biotin-Labelled cRNA was normalized to a concentration of 150 ng/μL and 750 ng were hybridized to Illumina Human-12 v4 BeadChips for 16 h (overnight) at 58°C. Following hybridization, BeadChips were washed and stained with streptavidin-Cy3 (GE Healthcare). BeadChips were then scanned using the BeadArray reader, and image data was then processed using Genome Studio software (Illumina).

Differential expression analysis of transcriptomic data

Probe summaries for all arrays were obtained from the raw data using the method "Making Probe Summary" in Genome Studio. These values were transformed (variance stabilized) and quantile normalized using the R/Bioconductor package lumi.[119] Standard lumi QC procedure was applied and no outliers were identified. Differential expression between pairs of conditions was evaluated using the R/Bioconductor package limma.[120] A linear model fit was applied, and the top differentially expressed genes were tabulated for each contrast using the method of Benjamini and Hochberg to correct the p values.[121] Probes that failed to fluoresce above background in both conditions were removed. Differentially expressed probes were selected using a cutoff of adjusted p value < 0.01 and absolute fold-change > 2. The raw and processed microarray data are publicly available on ArrayExpress (Accession number: E-MTAB-3586).

Gene enrichment analysis

Gene enrichment analysis was performed using DAVID[122,123] to estimate the significant enrichment of terms in the Gene Ontology Biological Process and KEGG pathway databases. Multiple probes mapping to the same gene were collapsed, and significant enrichment was inferred when p value < 0.05.

Principal-component analysis

Principal-component analysis (11 principal components, capturing 95% of the total variability across samples) was performed with Perseus software using $\log_2$ normalized expression values of differentially regulated probes (one-way ANOVA significant with p value < 0.01). In order to obtain a biological interpretation of PC1 and PC2, the top 5% probes positively or negatively correlated with either axis were used for gene enrichment analysis as described above.

DAPT treatment assay

NOTCH signaling was inhibited pharmacologically using the small molecule DAPT, a well established gamma-secretase inhibitor. Cells were treated with 10 μM DAPT 24 h prior to the time of desired effect. DMSO was used as a control.

Recombinant proteins

The recombinant human WNT4 protein (RnD Systems; Cat. No. 6076-Wn; >60% purity), recombinant human WNT8A (Genemed PlexBio; Cat No. 90007-02; >95% purity). According to RnD Systems WNT4 product information, the typical ED_{50} for this WNT4 protein is 25–100 ng/mL, so we chose to proceed with the upper limit of ED_{50} in our experiments for WNT proteins.

DNA constructs

M50 Super 8x TOPFlash was a gift from Randall Moon (Addgene plasmid # 12456), M51 Super 8x FOPFlash (TOPFlash mutant) was a gift from Randall Moon (Addgene plasmid # 12457). 4xCSL-luciferase was a gift from Raphael Kopan (Addgene plasmid # 41726). 408 pSG5L HA E2F1 was a gift from William Sellers (Addgene plasmid # 10736; http://n2t.net/addgene:10736; RRID:Addgene_10736). 409 pSG5L HA RB (379–928) was a gift from William Sellers (Addgene plasmid # 10734; http://n2t.net/addgene:10734; RRID:Addgene_10734). 584 pSG5L HA RB 661W was a gift from William Sellers (Addgene plasmid # 10731; http://n2t.net/addgene:10731; RRID:Addgene_10731). 608 pSG5L HA RB del ex4 was a gift from William Sellers (Addgene plasmid # 10730; http://n2t.net/addgene:10730; RRID:Addgene_10730). 500 pSG5L HA RB del685 (NAAIRS) was a gift from William Sellers (Addgene plasmid # 10729; http://n2t.net/addgene:10729; RRID:Addgene_10729). 498 pSG5L HA RB del663 (NAAIRS) was a gift from William Sellers (Addgene plasmid # 10728; http://n2t.net/addgene:10728; RRID:Addgene_10728). 496 pSG5L HA RB del651 (NAAIRS) was a gift from William Sellers (Addgene plasmid # 10726; http://n2t.net/addgene:10726; RRID:Addgene_10726). 432 pSG5L HA RB 567L was a gift from William Sellers (Addgene plasmid # 10725; http://n2t.net/addgene:10725; RRID:Addgene_10725). 416 pSG5L HA RB del22 was a gift from William Sellers (Addgene plasmid # 10721; http://n2t.net/addgene:10721; RRID:Addgene_10721). 413 pSG5L HA RB was a gift from William Sellers (Addgene plasmid # 10720; http://n2t.net/addgene:10720; RRID:Addgene_10720).

Treatment of cells with NOTCH ligand DLL1

Purified DLL1 (RnD Systems) was bound to Protein G Agarose beads (1μg of DLL1 per 2.5μl of packed beads) for 2h at 4°C rotating and incubated with cells at a ratio of 2.5μl of packed beads (1xconcentration) per 12-well plate well, using 0.5mL of media per well. Beads were aspirated with media and cells were washed once with PBS before collection and analysis. We transfected cells with a NOTCH-responsive promoter construct containing 4xCSL binding sites (Addgene plasmid # 41726) to study the activation of NOTCH pathway. These results confirmed that DLL1 bound to agarose beads activates the NOTCH pathway.

Depletion of WNT ligands from media

WNT4 and WNT8A specific antibodies were bound to Protein G Agarose beads (1μg of antibody per 2.5 μl of packed beads) for 2h at 4°C rotating and used at a ratio of 1μg of each antibody per 1 mL of collected media. Control depletion was carried out with an IgG antibody and confirmed by western blotting. We analyzed a panel of WNT target genes axin2, sfrp1, frat by qPCR for evidence that the WNT pathway activation was decreased by WNT ligand depletion from the media. IWP2 conditioned medium showed a similar reduced effect as WNT depleted medium.

Cell incubation with collected media

Media was incubated with 70–80% confluent cells for 24h, before collection. The media collected from cells, aliquoted, stored at −80°C, and thawed freshly just before use. Cells were cultured in the collected media not more than 24h before substituting for a fresh aliquot. To avoid possible autocrine effects via WNT signaling due to inconsistent cell density (the more cells the higher levels of WNT in media), particular care should be taken for plating cells at the same density across the experimental samples. Since HESCs are passaged as small colonies (~25–50 cells per colony), growth/passaging conditions were kept as similar as possible by plating the same number of colonies for each condition. By counting the total cell numbers per sample we observed a less than 10% fluctuation in our passaging technique.

Luciferase assay

Cells were transfected with a SMAD2/3 reporter construct (SBE4-luciferase), SOX17 or GSC promoter constructs[117] and Renilla luciferase at a ratio of 10:1, using Lipofectamine 2000 (Invitrogen).[124] Luciferase activity was measured with the dual luciferase assay kit following (Promega) manufacturer instructions. Firefly luciferase activity was normalized to Renilla luciferase activity for cell numbers and transfection efficiency. Samples were analyzed on a Glomax Luminometer and software. We used a 500bp actin promoter region driving luciferase expression as a negative control for WNT/β-catenin responsive gene.

Transwell assays

Cancer cell invasiveness was analyzed by using a modified Boyden chamber-based assay, CultureCoat 96 Well High BME Cell Invasion Assay (Trevigen, Cat. No: 3483-096-K) or 24-well transwell inserts with 8μm pores (Sarstedt, Cat no. 83.3932.800) with EHS Matrix Extract as basal membrane (Merck) according to manufacturer's guidelines. 25,000 cells were used per well and incubated for 24h before analysis. Cancer cells were placed in the media collected from their corresponding cell lines.

Western blot analysis

Protein was isolated by lysing cells with RIPA Buffer (Sigma-Aldrich) supplemented by cOmplete EDTA-free protease inhibitor (Roche) and PhosSTOP (Sigma-Aldrich) and extracting the supernatant after high-speed centrifugation at 4°C. Protein quantification was performed using the Pierce BCA Protein Assay kit following the manufacturer's protocol. Isolated proteins were prepared for SDS-PAGE separation by dilution with 4× NuPAGE Sample buffer (Invitrogen), addition of NuPAGE Sample Reducing Agent ((10X), Invitrogen), 95°C for 5 min, and cooling. Isolated proteins were then analyzed by Western blotting. Protein separation via SDS-PAGE was performed on a NuPAGE 4%–12% or 12% Bis-Tris gel (Life Technologies) with NuPAGE MOPS SDS Running Buffer (Life Technologies). Proteins were transferred to a PVDF membrane, blocked with 5% milk in PBS and 0.05% tween 20, probed with protein-specific antibodies, incubated with horseradish peroxidase-conjugated secondary antibodies, and visualized via enhanced chemiluminescence using the SuperSignal West Pico Chemiluminescent Substrate (Thermo Scientific). All antibodies were diluted in 5% milk in PBS and 0.05% tween 20. Quantification was performed using ImageJ gel analysis tool.

Protein co-immunoprecipitation

Cells were harvested with trypsin and washed twice with cold PBS. For cytoplasmic lysis, cells were suspended in 5 times packed cell volume (1 μl PCV = 10^6 cells) equivalent of Isotonic Lysis Buffer (10 mM Tris HCl, pH 7.5, 3 mM $CaCl$, 2 mM $MgCl_2$, 0.32 M Sucrose, Complete protease inhibitors and phosphatase inhibitors), and incubated for 12 min on ice. Triton X-100 was added to a final concentration of 0.3% and incubated for 3 min. The suspension was centrifuged for 5 min at 1,500 rpm at 4°C and the supernatant (cytoplasmic fraction) transferred to a fresh chilled tube. For nuclear lysis, nuclear pellets were resuspended in 2 x PCV Nuclear Lysis Buffer+Triton X-100 (50 mM Tris HCl, pH 7.5, 100 mM NaCl, 50 mM KCl, 2 mM $MgCl_2$, 1 mM EDTA, 10% Glycerol, 0.3% Triton X-100, Complete protease inhibitors and phosphatase inhibitors) and dounce homogenized. The samples were incubated with gentle agitation for 30 min at 4°C and then centrifuged with a Ti 70.1 rotor at 22,000 rpm for 30 min at 4°C or with a Ti 45 rotor for 30 min at 20,000 rpm at 4°C. The chromatin pellets were dounce homogenized in 2 x PCV Nuclear Lysis Buffer+Triton X-100 and Benzonase

until the pellets gave much less resistance. The samples were incubated at RT for 30 min and centrifuged with either a Ti 70.1 rotor for 30 min at 22,000 rpm at 4°C or with a Ti 45 rotor for 30 min at 20,000 rpm at 4°C. Samples were incubated with 5 μg of cross-linked antibodies for 12 h at 4°C. Beads were washed five times with ten bead volumes of Nuclear Lysis Buffer and eluted in SDS western blotting buffer (30 mM Tris pH 6.8, 10% Glycerol, 2% SDS, 0.36 M beta-mercaptoethanol (Sigma), 0.02% bromophenol blue) by heating at 90°C for 5 min. Samples were analyzed by standard western blotting techniques. As an alternative method, we also used the nuclear complex co-IP kit (Active Motif, cat. 54001) according to the guidelines.

ATAC-sequencing

Cells were washed once with PBS, collected in Cell Dissociation Buffer (Gibco 13150-016) or TrypLE and centrifuged at 300g for 3 min. The cell pellets were then resuspended in 2 mL of 4°C PBS and counted by haemocytometer for using 100,000 cells in the subsequent step. Cells were centrifuged at 300g for 3 min, the supernatant aspirated, the cell pellet resuspended in 150 μl of Isotonic Lysis Buffer (10 mM Tris-HCl pH 7.5, 3 mM CaCl, 2 mM $MgCl_2$, 0.32 M Sucrose and Protease Inhibitors, Roche), and incubated for 12 min on ice. Triton X-100 from a 10% stock was then added at a final concentration of 0.5%, the samples were vortexed briefly and incubated on ice for 6 min. The samples were centrifuged for 5 min at 400 g at 4°C, and the cytoplasmic fraction removed from the nuclear pellet. The samples were resuspended gently in 625uL of PBS and transferred to a fresh 1.5 mL eppendorf tube. The nuclei were centrifuged at 1500g for 3 min at 4°C and the supernatant aspirated thoroughly from the nuclear pellet. This step was immediately followed by tagmentation (Nextera DNA Sample Preparation Kit for 24 Samples, FC-121-1030) by resuspending each sample in 100 μL Nextera mastermix (52.5 μL TD buffer, 42.5 μL of water and 5 μL of TDE1 per reaction). The nuclear pellet was resuspended thoroughly by pipetting and incubated at 37°C for 1 h shaking at 300rpm. The reaction was stopped with 300 μL of buffer PB from the Qiagen PCR purification kit, followed by Qiagen PCR clean up protocol using MinElute columns and eluting each sample in 18 μL buffer EB. For the control sample, the nuclear pellet was subjected to genomic DNA isolation with GenElute Mammalian Genomic DNA Miniprep Kit (Sigma, G1N70) according to manufacturer's protocol, and the purified genomic DNA was thereafter immediately used for tagmentation as for other ATAC-seq samples.

Next a PCR reaction (for all samples including control sample) was performed with the following constituents: 10 μL template from tagmentation, 2.5 μL I7 primer (Nextera Index Kit with 24 Indices for 96 Samples, FC-121-1011), 2.5 μL I5 primer, 10 μL Nudease Free H_2O 25μL NEBNext High-Fidelity 2x PCR Master Mix (New England Labs Cat #M054 and 10 μL Nuclease Free H_2O. The PCR settings were as follows: at 72°C for 5 min, initial denaturation at 98°C for 30 s, then 12 cycles of 98°C for 10 s, primer annealing at 63°C for 30 s and elongation at 72°C for 1 min, and holding at 4°C. After completing the PCR, the sample were stored at −20°C. The PCR primers were removed with 1 x 0.9:1 SPRI beads (Beckman Coulter, Cat no. A63880) according to manufacturer's protocol and samples eluted in 20 μL. 2 μL of the samples were run on Agilent HS Bioanalyzer HS for confirming the size selection of the ATAC libraries. ATAC-sequencing was performed by Illumina HiSeq 2000 sequencing with 75 bp PE for obtaining more than 40 million mapped reads per library.

ATAC-sequencing analysis

Sequencing reads from the ChIP-seq and ATAC seq experiment were aligned to the human genome (hg38) using bowtie with reporting mode," –best –strata –v2". Deeptools was used to generate covergae track(bigwig). Coverage track was visualized by using UCSC genome browser. Peak calling was performed by using macs2 peak caller with default parameters for ChIP seq, and with parameter "–nomodel –shift −100 –extsize 200" for ATAC seq. Peaks annotated with nearest gene information by using BEDTools. Peak distribution over different genomic features were summarized by using Bioconductor package ChiPpeakAnno. Motif enrichment analysis within peak regions was performed using HOMER. All plots were generated using R package 3.6.

ChIP-sequencing and ATAC-sequencing visualization

The genomic tracks of sequencing data were visualized by using the UCSC Genome Browser on Human (GRCh38/hg38) and NGS data available via the Cistrome Data Browser: Human E2F1 ChIP-seq,[52,53] E2F4 ChIP-seq (ENCODE: GSM935400),[54] β-catenin ChIP-seq[125] and ATAC-seq for neuroectoderm (post-mesendoderm competency loss).[126] ATAC-seq of undifferentiated H9 hESCs were performed in-house the at the University of Oxford.

The small molecule screening library

The screening library contained concentrated small molecule compounds with verified biochemical activity against their targets. Most of the compounds target epigenetic regulators with high specificity (Table S3).

Screening of the chemical compounds

The cells were grown in 96-well plates in standard growth medium with puromycin (1 μg/mL stock). Three technical replicates and three biological replicates were used for the screening. Cells were plated at a concentration of 10,000 cells in 100 μL of media per well in a 96-well plate. One day after plating the cells, the medium was changed to 90 μL standard growth medium supplemented with puromycin (0.5 μg/mL) and Activin A (10 ng/mL). On the same day, the compounds were added: first, 100x compound library dilutions were made, and 10μL of 100x diluted chemical was added to each well to obtain 1000x final dilution of the compounds. Cells were then cultured with chemical compounds for five days with media change at day 0, day 2 and day 4 supplemented by fresh

compounds. Each replicate was analyzed using Celigo Image Cytometer (Nexcelom) and flow cytometry. Cells were lifted and dissociated into single cells with Trypsin. Details on the antibodies that were used for flow cytometry are listed in Table S3. The cells were incubated with 0.5 μg/mL final concentration of conjugated antibodies in 1% BSA-PBS for 40 min on ice and washing was repeated as before. The cells were then suspended in 300 μL 1% BSA-PBS with DAPI (1:2000) for live/dead separation and kept on ice to be used for the flow cytometry analysis.

QUANTIFICATION AND STATISTICAL ANALYSIS

GraphPad Prism was used for statistical analysis as stated in figure legends. Unless otherwise indicated in the figure legends, we analyzed three biological replicates for each data point in all graphs. **** marks adjusted p value <0.0001, *** is adjusted p value <0.001, ** is adjusted p value <0.01, * is adjusted p value <0.05.

Cell Reports, Volume *42*

Supplemental information

RBL2-E2F-GCN5 guide cell fate decisions during tissue specification by regulating cell-cycle-dependent fluctuations of non-cell-autonomous signaling

Stefania Militi, Reshma Nibhani, Morteza Jalali, and Siim Pauklin

SUPPLEMENTARY INFORMATION

Supplementary Table 3. Antibodies, primers and chemical compounds.

**Antibody name**	**Techniques**	**Catalogue name**	**Company**
Goat anti-human Nanog	IF	AF1997	R&D Systems
Mouse anti-human Oct4	IF	sc-5279	Santa Cruz Biotechnology
Goat anti-human SOX2	IF	AF2018	R&D Systems
Rabbit anti-human Eomes	IF	ab23345	Abcam
Goat anti-human Brachyury	IF	AF2085	R&D Systems
Goat anti-human SOX17	IF	AF1924	R&D Systems
PAX6 rabbit polyclonal	IF, WB	PRB-278P-100	Cambridge BioScience
pRb mouse monoclonal	IF, WB	554136 (G3-245)	BD Pharmingen
RBL1/p107 (C-18) rabbit polyclonal	IF, WB	sc-318	Santa Cruz Biotechnology
RBL2/p130 (C-20) rabbit polyclonal	IF, WB, CHIP	sc-317	Santa Cruz Biotechnology
SOX1 goat polyclonal	IF	AF3369	R&D Systems
Actin mouse monoclonal	WB	MAB1501	Chemicon
E2F1 (C-20)	WB, CHIP	sc-193	Santa Cruz Biotechnology
E2F4 (A-20)	WB, CHIP	sc-1082x	Santa Cruz Biotechnology
SOX1 goat polyclonal	IF, WB	AF3369	R&D Systems
p75 (C-20) goat polyclonal	WB	sc-6188	Santa Cruz Biotechnology
WNT4 (m-70) rabbit polyclonal	WB	sc-13962	Santa Cruz Biotechnology
WNT5A (H-58) rabbit polyclonal	WB	sc 30224	Santa Cruz Biotechnology
WNT8A rabbit polyclonal	WB	SAB1411397	Sigma
HES5 rabbit polyclonal	WB	ab25374	Abcam
DLL1 H-265 rabbit polyclonal	WB	sc-9102	Santa Cruz Biotechnology
DLL3 H-110 rabbit polyclonal	WB	sc-67270	Santa Cruz Biotechnology
P-ser33-B-cat rabbit polyclonal	WB	sc-16743-R	Santa Cruz Biotechnology
B-catenin (H-102) rabbit polyclonal	WB, CHIP	sc-7199	Santa Cruz Biotechnology
Beta Tubulin 3/ Tuj1 [GT1338] mouse monoclonal	IF	GTX631831-GTX	Stratech
CD133-BV786, Mouse Anti-Human, clone W6B3C1	FACS	BD 747640	BD Biosciences
Mouse IgG1-BV786l	FACS, isotype control for CD133	BD 563330	BD Biosciences
Alexa647 Mouse anti-SSEA-4 clone	FACS	BD 560219	BD Biosciences
Alexa647 Mouse IgG3	FACS, isotype control for SSEA4	BD 560803	BD Biosciences

Primer name	Techniques	
pRB	qPCR	F ccaggccccctacctttgtcacc<br>R ttgttggtgttggcagaccttct
RBL1/p107	qPCR	F ccaagaaagcgctctgctgtacaa<br>R acagacgcgtttggcagggg
RBL2/p130	qPCR	F cccctctgatggagggacgcc<br>R ttggctgtgacagtggcggt
PBGD	qPCR	F ggagccatgtctggtaacgg<br>R ccacgcgaatcactctcatct
Nanog	qPCR	F catgagtgtggatccagcttg<br>R cctgaataagcagatccatgg
OCT4	qPCR	F agtgagaggcaacctggaga<br>R acactcggaccacatccttc
SOX2	qPCR	F tggacagttacgcgcacat<br>R cgagtaggacatgctgtaggt
Eomes	qPCR	F atcattacgaaacagggcaggc<br>R cggggttggtatttgtgtgtaagg
Gsc	qPCR	F gaggagaaagtggaggtctggtt<br>R ctctgatgaggaccgcttctg
SOX17	qPCR	F cgcacggaatttgaacagta<br>R ggatcagggacctgtcacac
Brachyury T	qPCR	F tgcttccctgagacccagtt<br>R gatcacttctttcctttgcatcaag
Mesp1	qPCR	F gaagtggttccttggcagac<br>R tcctgcttgcctcaaagtgt
SOX1	qPCR	Sigma Quantitect primers
PAX6	qPCR	F ctttgcttgggaaatccgag<br>R agccaggttgcgaagaactc
SOX9	qPCR	F aggaagtcggtgaagaacggg<br>R ctctcgcttcaggtcagcct
SOX10	qPCR	F atccaggcccactacaagagc<br>R actggtccaactcagccacat
P75	qPCR	F acaagacctcatagccagcac<br>R ctgttggctccttgcttgttc
HNK1	qPCR	F cgacgacgacaacacctaca<br>R cctggtagcctcccttcaca
Olig3	qPCR	F agccgtctcaactcggtct<br>R catggctaggttcaggtcgtg
Otx2	qPCR	F atcccttgtccacctcctct<br>R gctggtgatgcataggggtc
HES5	qPCR	Sigma Quantitect primers
Delta1	qPCR	F acctcgcaacagaaaaccca<br>R gtgttcgtcacacacgaagc
Delta3	qPCR	F cggatgcactcaacaacct<br>R gaagatggcaggtagctcaa
WNT4	qPCR	F cgtcttcggcaaggtggtga<br>R ctgaccccatgcactgtcct
WNT5A	qPCR	F tgtgccacttgtatcaggacc<br>R tgcctatctgcatcaccctg
WNT8A	qPCR	F ctgtggctgtgatgggtcaa<br>R ttcatcagggctctggcatc
WNT3	qPCR	F ttgttccaactattgggggc<br>R gctgtgagcccagagatgtg
SFRP1	qPCR	F gtcatgcagttcttcggcttc<br>R ctcgttgtcacagggaggac
FRAT2	qPCR	F gtgctctcgggaaacctcat<br>R caaggagcctgagggctg
FOXD3	qPCR	F actgcttcgtcaagatcccc<br>R ctgtaagcgccgaagctct

KAL1	qPCR	F ttcaaagacgacgacccact<br>R ttttcgtgggtcatgccaga
TFAP2A	qPCR	F ccaagtccaacagcaatgcc<br>R gttgagacactcgggtggtg
TFAP2C	qPCR	F gccgtaatgaaccccactga<br>R accggcctccatttttcgat
OLIG3	qPCR	F atgcacgacctgaacctagc<br>R ccccatagatctcgccaacc
ROR1	qPCR	F cccagaagctgcgaactgta<br>R gtgtgtgtggggatactggg
CXCL12	qPCR	F cgtcagcctgagctacagat<br>R tagcttcgggtcaatgcaca
ZIC3	qPCR	F gcaaagtgtgcgacaagtcc<br>R tgcacagtaggttcggcatt
ASCL1	qPCR	F ctcaacttcagcggctttgg<br>R cgcagtgtctccaccttact
NEUROD1	qPCR	F cgctttgcaagggcttatcc<br>R aggcgactggtaggagtagg
NEUROG1	qPCR	F aatatctcccgggcgtctga<br>R gttcaagttgtgcatgcggt
FGF8	qPCR	F gctgcagaatgccaagtacg<br>R ggcgggtagttgaggaactc
SOX3	qPCR	F gataagcctacccttcccgc<br>R gtgtccctacggggttcttg
FOXD1	qPCR	F tctgtgagttcatcagcggc<br>R gcaggaagctgccgttgtc
ID1	qPCR	F ccagcacgtcatcgactaca<br>R acacaagatgcgatcgtccg
RORB	qPCR	F acgtcattgacctgcccaag<br>R cctggtgctaactgcccatt
PAX6	ChIP-qPCR	F ttcgggtctctccgatgaag<br>R gaaactagtcttgccgagtgc
SOX1	ChIP-qPCR	F gctagaagttgcagcctccga<br>R caagttgcagctccgcttttg
SOX9	ChIP-qPCR	F ggcattccgagagtacgaca<br>R gcagctgattggacccgatt
SOX10	ChIP-qPCR	F cctgtgttgctgcatcccc<br>R agggagtaggcgcttaagga
Olig3	ChIP-qPCR	F ctccgggtgagtcaaaccac<br>R gactcccgttttagctgactt
P75	ChIP-qPCR	F gagagtgaaccctgtggcg<br>R ttagagcctctcacccatccc
HoxD1	ChIP-qPCR	F tcaagggaaagacgtgagcc<br>R ttaccggggacgggtgagat
HoxA2	ChIP-qPCR	F acagaacttatgtggctggga<br>R gcattgtttgggactgtcgg
HES5	ChIP-qPCR	F gaagaagggtggggtccttg<br>R aagctagtgaaaggcctggg
DLL1	ChIP-qPCR	F cgcttgcatttcctccttgc<br>R ttttcagtcccgtgtttccg
DLL3	ChIP-qPCR	F gtttgggagggtggctttgt<br>R gtgcgctggtttgtgtgaat
WNT4	ChIP-qPCR	F gccaagagagcttccctaaact<br>R atccgaaacctcgcttctgg
WNT3	ChIP-qPCR	F tgaacccctcaaggaggaga<br>R acggagccgagtgtcatttg
WNT8A	ChIP-qPCR	F ctgggtggccctaaggttg<br>R ccatctctgcaacagtccct
SFRP1	ChIP-qPCR	F gaggccttggagaggaacat<br>R cagacatcacgcctacgcaa

Otx2	ChIP-qPCR	F cctttaccctttctggccgt<br>R gcgggttagggagtgactg
DLL1 F	Luciferase assay	TCTTACGCGTGAGGCGTAGTTACTTGGCTTTGCCTTAGAGCGGA
DLL1 R	Luciferase assay	TCTCGAGCTAGTGTATCTATGGGTTCCCCCTCACCATTTTCTTGTTT TTTCCTTGC
DLL3 F	Luciferase assay	TCTTACGCGTGATGTGAAGACGGAATTTCCTGCCCATTTGCTCCTC
DLL3 R	Luciferase assay	TCTCGAGCTAGTGAGAGAATGGCCCCGCCCCTTCAGGC
WNT4 F	Luciferase assay	TCTTACGCGTGCTAGTATGGGAGGATAAATCGAACAAGCACATAGAA AACCAAATGCAAA
WNT4 R	Luciferase assay	TCTCGAGCTAGCGGCGGCGGAGGCGGGCG
WNT8A F	Luciferase assay	TCTTACGCGTGTAAATAAATAAATAAATAAATAAATAAATAAAATAAAATAAAAGACAGATTCCCACCTAAGGAGCTGAAGTCCTAGTAA
WNT8A R	Luciferase assay	TCTCGAGCTAGATTTCTTTCTTCTATATGTCAATTTTGTCTCTTCCTGCTTCTTTGGCCC

**Source Name**	**[Working] uM**	**Class/Target**
(+)-JQ1	1	Bromodomains - BRD2, BRD3, BRD4, BRDT (BET)
(-)-JQ1 (inactive)	1	Bromodomains - Negative control
PFI-1	5	Bromodomains - BRD2, BRD3, BRD4, BRDT (BET)
I-BET	1	Bromodomains - BRD2/3/4
Bromosporine	1	Bromodomains - pan-Bromodomain
CBP/BRD4 (0383)	5	Bromodomains - CBP, BRD4(1)
SGC-CBP30	1	Bromodomains - CREBBP, EP300
I-CBP112	1	Bromodomains - CREBBP, EP300
RVX-208	5	Bromodomains - BRD2, BRD3, BRD4, BRDT (BET, BD2)
SMARCA	2.5	Bromodomains - SMARCA, PB1
PB1/SMARCA	1	Bromodomains - SMARCA, PB1
PFI-3	1	Bromodomains - SMARCA2/4, PB1(5)
GSK2801	1	Bromodomains - BAZ2A, BAZ2B
PFI-4	1	Bromodomains - BRPF1B
TRIM24/BRPF	10	Bromodomains - TRIM24/BRPF
OF-1	5	Bromodomains - pan-BRPF
Belinostat	5	HDAC - hydroxamic acids
CXD101	1	HDAC
Valproic acid	1000	HDAC - aliphatic acid compounds
Entinostat	0.5	HDAC - ortho-amino anilides
SAHA	2.5	HDAC - hydroxamic acids
Trichostatin A	0.5	HDAC - hydroxamic acids - Class I & II
SRT1720	1	HDAC - SIRT1 activator
EX 527	1	HDAC - SIRT1
CI-994	1	HDAC - 1,2,3,(8)

CPI-360	10	Histone methyltransferase - EZH2 and EZH1
CPI-413	10	Histone methyltransferase - EZH2 and EZH1
UNC0638	1	Histone methyltransferase - G9a, GLP
UNC0642	1	Histone methyltransferase - G9a, GLP
A-366	2	Histone methyltransferase - G9a, GLP
Chaetocin	0.05	Histone methyltransferase - SUV39H1
PFI-2	2	Histone methyltransferase - SETD7
SGC0946	7.5	Histone methyltransferase - DOT1L
GSK343	3	Histone methyltransferase - EZH2
UNC1999	1	Histone methyltransferase - EZH2
LLY-507	1	Histone methyltransferase - SMYD2
Tranylcypromine	20	Lysine demethylases - LSD1
GSK-LSD1 (irreversible)	0.5	Lysine demethylases - LSD1
GSK690	5	Lysine demethylases - LSD1
GSK J4	10	Lysine demethylases - JMJD3, UTX, JARID1B
GSK J5 (inactive)	10	Lysine demethylases - Negative control
IOX1 (5-carboxy-8HQ)	40	Lysine demethylases - pan-2-OG
Methylstat (Ester)	2.5	Histone demethylase
(E)-JIB-04	0.05	Histone demethylase - Pan JmjC
ML324	5	Histone demethylase - JMJD2E
IOX2	10	Prolyl-Hydroxylases - PHD2 (EGLN1)
OICR-9429	1	Methyl Lysine Binder - WDR5
UNC1215	5	Methyl Lysine Binder - L3MBTL3
5-Azacitidine	10	DNA methyltransferase (DNMT)
5-Azadeoxycitidine	5	DNA methyltransferase (DNMT) - DNMT1/3
Olaparib	1	Poly ADP ribose polymerase (PARP)
Rucaparib	10	Poly ADP ribose polymerase (PARP)
K00135	1	Kinase inhibitor - ATP competitive - PIM
5-Iodotubercidin	1	Kinase inhibitor - ATP mimetic - Haspin
C646	1	Histone acetyltransferase (HAT) - p300/CBP
DUAL1946	1	
GSK484	1	Peptidyl arginine deiminase (PAD4)
KDOBA67	10	Histone demethylase
BAZ2-ICR	1	Bromodomains - BAZ2A, BAZ2B
NI-57	1	Bromodomains - pan-BRPF
LP99	1	Bromodomains - BRD9, BRD7
SGC707	1	Arginine methyltransferase - PRMT3
RGFP966	10	HDAC - HDAC3
PCI-34051	5	HDAC - HDAC8

Rocilinostat	10	HDAC - HDAC6
Tubastatin A HCl	10	HDAC - HDAC6
KDOAM-25a	1	Lysine demethylases - JARID
KDM5-C70	10	Histone demethylase - JARID1
MAZ1805	1	t-RNA sythetase
MAZ1392	1	t-RNA sythetase
BI-9564	1	Bromodomains - BRD9, BRD7
NVS-CECR2-1	1	Bromodomains - CECR2
GSK106	1	Peptidyl arginine deiminase (PAD4)
J556-42R	1	Arginine methyltransferase - PRMT5
J556-63R	1	Arginine methyltransferase - PRMT5
J556-70R	1	Arginine methyltransferase - PRMT5
A-196	1	Histone methyltransferase - SUV420H1/H2
BAY-598	1	Histone methyltransferase - SMYD2
J556-143	1	Arginine methyltransferase - PRMT5
MS049	1	Arginine methyltransferase
MS023	1	Arginine methyltransferase - Type I PRMTs
MS003	1	Arginine methyltransferase - negative control
SGI-1776	10	Kinase inhibitor - Haspin
CHR-6494	1	Kinase inhibitor - Haspin
CPI-169	10	Histone methyltransferase - EZH2, EZH1
UNC2400	1	Histone methyltransferase - EZH2
GSK864	5	Dehydrogenase
GSK8814	10	Bromodomains - ATAD2
GSK8815	10	Bromodomains - ATAD2
GSK959	1	Bromodomains - BRPF1
NVS-CECR2-C	1	Bromodomains - CECR2
BAY-299	1	Bromodomains - BRD1, TAF1
PCI-24781	10	HDAC
Romidepsin	1	HDAC
Mocetinostat	10	HDAC
Santacruzamate	50	HDAC
KDOAM32	1	Lysine demethylases - JARID
MS409N	1	Arginine methyltransferase - PRMT4, PRMT6 inactive control
TP-064	1	Arginine methyltransferase - PRMT4
TP-064N	1	Arginine methyltransferase - PRMT4
A-395	1	Methyl Lysine Binder - EED
A-395N	1	Methyl Lysine Binder - EED
I-BRD9	10	Bromodomains - BRD9

TP-472	1	Bromodomains - BRD9
TP-472N	1	Bromodomains - BRD9
KDOPZ-32a	1	Lysine demethylases - KDM5
KDOOA012000	1	Lysine demethylases - KDM2
AMI-1	50	Arginine methyltransferase - PRMT
TMP269	10	HDAC - 4, 5, 7, 9
AGK2	10	HDAC - SIRT2
GSK6853	1	Bromodomains - BRPF1/2/3
GSK9311	1	Bromodomains - BRPF1/2/3
LLY-283	1	Arginine methyltransferase - PRMT5
TD001851a	1	Methyl Lysine Binder/tudor domain - Spin1
TDOSI000058a	1	Methyl Lysine Binder/tudor domain - Spin1
TD001863a	1	Methyl Lysine Binder/tudor domain - Spin1
TDOSI000062a	1	Methyl Lysine Binder/tudor domain - Spin1
TD001857a	1	Methyl Lysine Binder/tudor domain - Spin1
TD001856a	1	Methyl Lysine Binder/tudor domain - Spin1
TD001858a	1	Methyl Lysine Binder/tudor domain - Spin1
TMP195	1	HDAC - 4,5,7,9
GSK2879552	10	Lysine demethylases - LSD1
TDO20821a	1	Methyl Lysine Binder/tudor domain - Spin1
TDO20824a	1	Methyl Lysine Binder/tudor domain - Spin1
TDO20823a	1	Methyl Lysine Binder/tudor domain - Spin1
A-485	1	Histone acetyltransferase (HAT) p300/CBP
A-486	1	Histone acetyltransferase (HAT) p300/CBP
GSK4027	1	Bromodomains - PCAF, GCN5
GSK4028	1	Bromodomains - PCAF, GCN5
L-Moses	1	Bromodomains - PCAF, GCN5
D-Moses	1	Bromodomains - PCAF, GCN5
PFI-5	1	Histone methyltransferase - SMYD2
YX39-31b	1	Methyl Lysine Binder/tudor domain - Spin1
TDO208229	1	Methyl Lysine Binder/tudor domain - Spin1
TDO01856a	1	Methyl Lysine Binder/tudor domain - Spin1
TDO20826a	1	Methyl Lysine Binder/tudor domain - Spin1
Bortezomib	0.1	Protesome
Carfilzomib	0.1	Protesome
RTS-V5	1	Protesome and HDAC
dBRD9	1	Bromodomains - BRD9
BI-7273	0.1	Bromodomains - BRD9/7
CPI-621	0.1	Lysine demethylases - KDM5

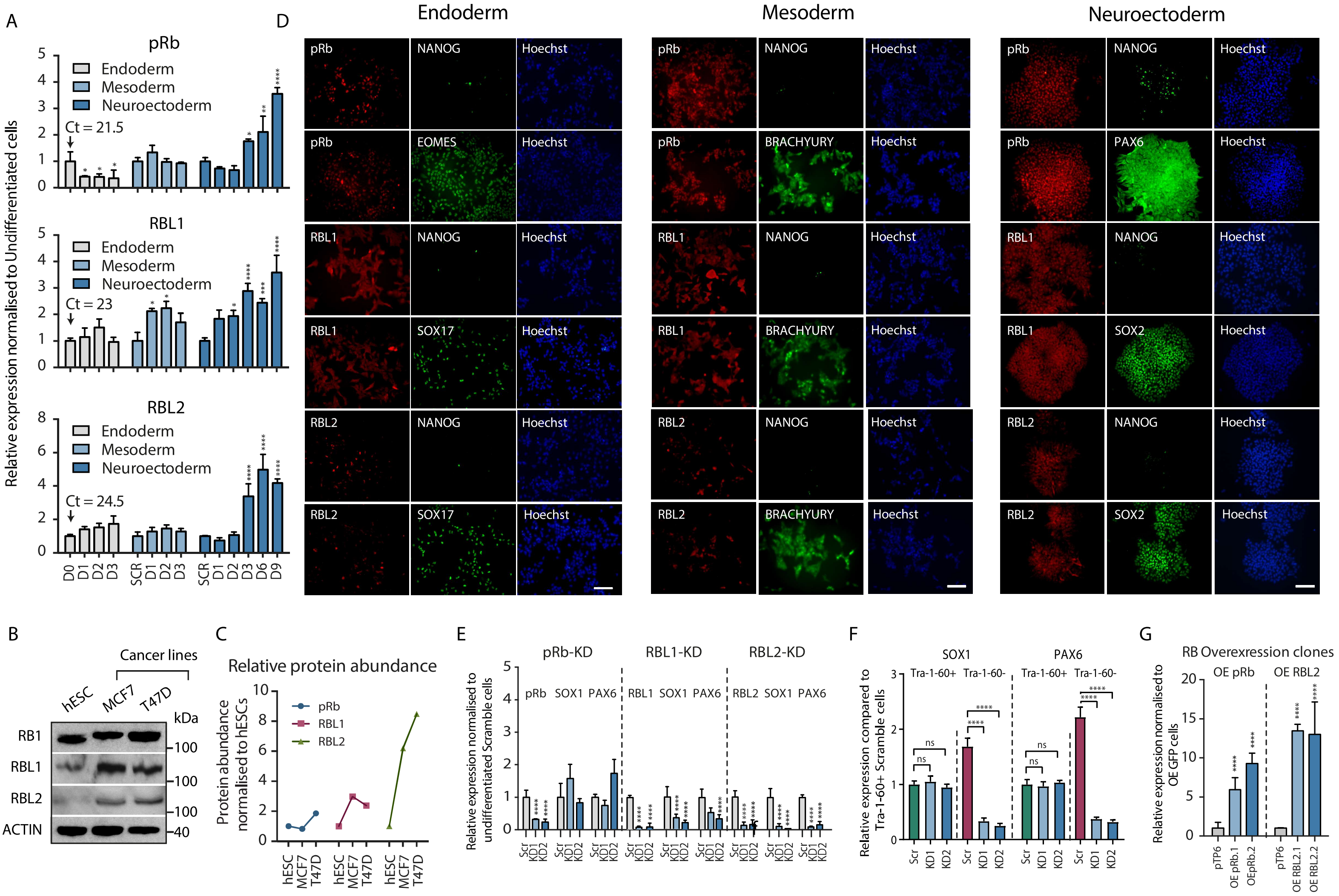

Figure S1: Functional studies of Retinoblastoma-family proteins in hPSCs, Related to Figure 1. (A) Rbl2 is upregulated during specification of human pluripotent stem cells to neuroectoderm. RB expression was analysed by Q-PCR. Ct values correspond to pRb, Rbl1 or Rbl2 expression in hPSCs. (B-C) RBs have different levels of expression in hPSCs. Western blot of RBs. Breast cancer lines were used as a comparison for relative protein expression. (C) Quantification of RB western blots by protein densitometry. (D) Immunostaining of RBs and germ layer specific markers during differentiation into endoderm, mesoderm and neuroectoderm. Scale bar 100 µm. (E) Effects of RB knockdown on background differentiation of hPSCs to neuroectoderm. Differentiation marker analysis by Q-PCR. (F) Effects of RBL2 knockdown on SOX1 and PAX6 expression in Tra-1-60 positive and negative cells. Cells were sorted or Tra-1-60 expression and analysed by qPCR. (G) Relative expression of pRb and Rbl1 in stable overexpression clones. All data are shown as mean+/-s.d. (n=3 biological replicates). Statistical analysis was performed by 2-way ANOVA with multiple comparisons with Tukey correction and **** marks adjusted P-value <0.0001, *** is adjusted P-value <0.001, ** is adjusted P-value <0.01, * is adjusted P-value <0.05.

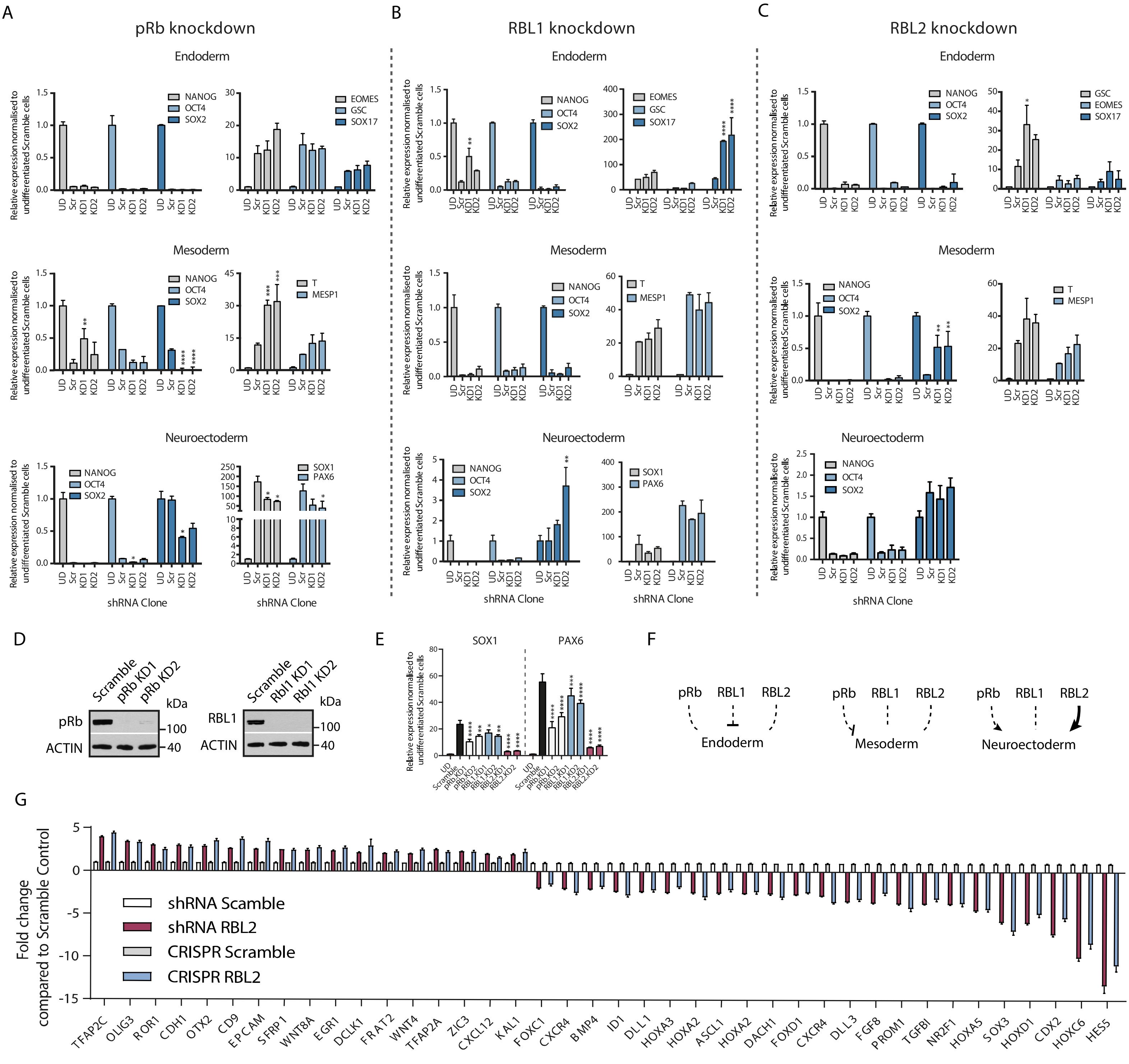

Figure S2: Analysis of RB KD on germ layer specification indicates a distinct function for RBL2 in neuroectoderm formation, Related to Figure 2. (A-C) Effect of RB KD during differentiation into three germ layers. Analysis of pluripotency and differentiation markers in (A) Rb KD cells, (B) RBL1 KD cells and (C) RBL2 KD cells. Significant differences compared to differentiated Scramble shRNA cells calculated by two-way ANOVA are marked. (D) Confirmation of pRb and RBL1 knockdown in stable knockdown HESCs. Western blot analysis of two knockdown lines compared to Scramble shRNA cells. (E) Comparison of the effects of Rb genes on neuroectoderm differentiation. Scramble and RB knockdown cells were side-by-side differentiated to neuroectoderm for 9 days and analysed by Q-PCR. Significant differences compared to differentiated Scramble shRNA cells calculated by two-way ANOVA are marked. (F) Schematic overview of the effects of Retinoblastoma-family proteins on germ layer differentiation. (G) Comparison of RBL2 knockdown effects on gene expression in RBL2 knockdowns achived by shRNA and CRISPR/Cas9. Cells were collected at day 4 of neuroectoderm differentiation and gene expression was normalised to control Scramble cells. All data are shown as mean+/-s.d. (n=3 biological replicates). Statistical analysis was performed by 2-way ANOVA with multiple comparisons with Tukey correction and **** marks adjusted P-value <0.0001, *** is adjusted P-value <0.001, ** is adjusted P-value <0.01, * is adjusted P-value <0.05.

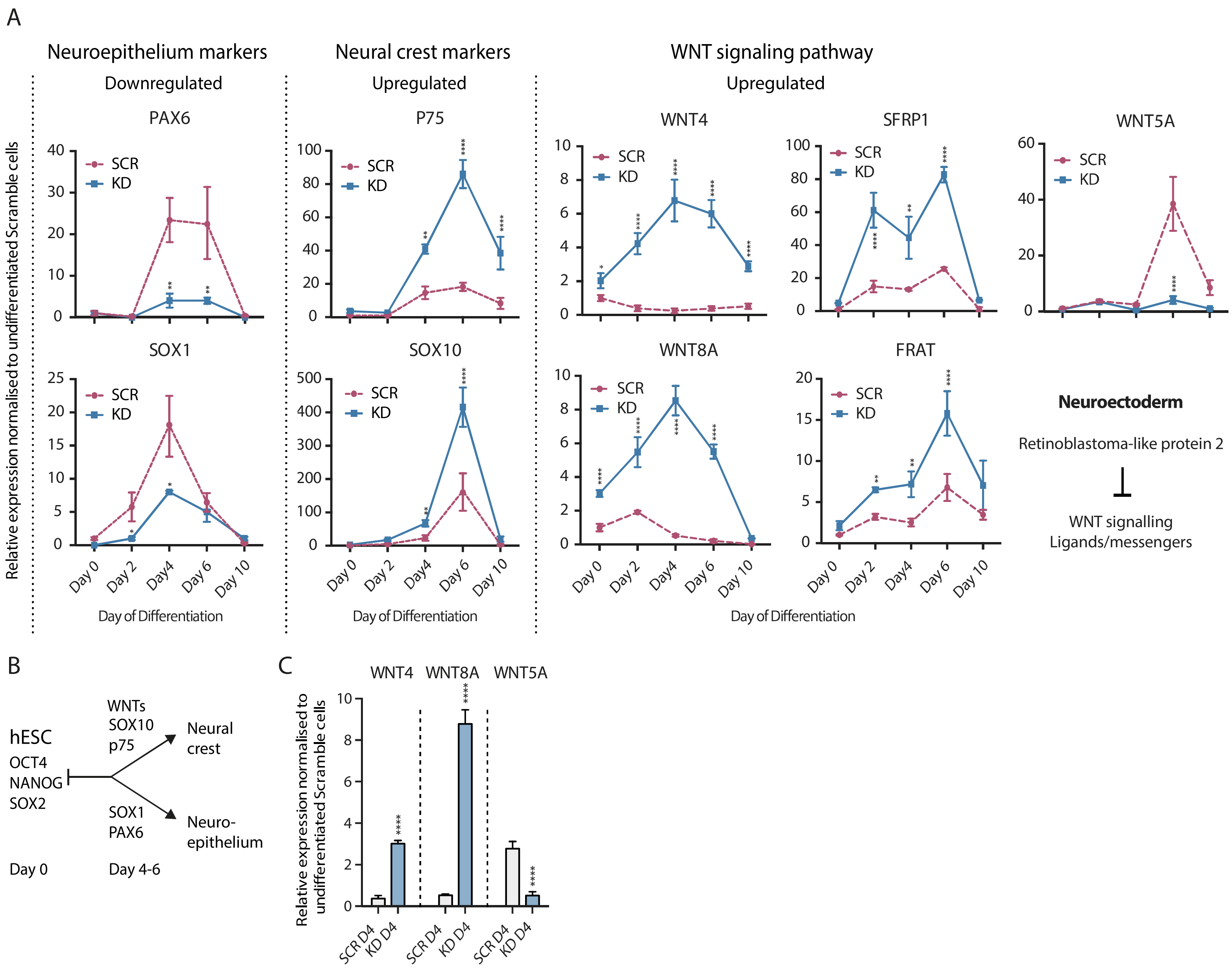

Figure S3: RBL2 represses WNT ligands WNT4 and WNT8A but leads to increased p75 and SOX10 expression, Related to Figure 2. (A) RBL2 controls the balance between neuroepithelial versus neural crest specification. Q-PCR throughout neuroectoderm differentiation of neuroepithelial markers SOX1, PAX6 and neural crest markers markers p75 and SOX10. Significant differences compared to Scramble shRNA cells calculated by two-way ANOVA are marked. (B) Schematic overview of the differentiation process and the markers characterising different cell types. (C) RBL2 KD increases the expression of canonical WNT pathway ligands WNT4 and WNT8A but decreases non-canonical WNT pathway ligand WNT5A during neuroectoderm differentiation. Significant differences compared to day 4 neuroectoderm Scramble shRNA cells calculated by two-way ANOVA are marked. All data are shown as mean+/-s.d. (n=3 biological replicates). Statistical analysis was performed by 2-way ANOVA with multiple comparisons with Tukey correction and **** marks adjusted P-value <0.0001, *** is adjusted P-value <0.001, ** is adjusted P-value <0.01, * is adjusted P-value <0.05.

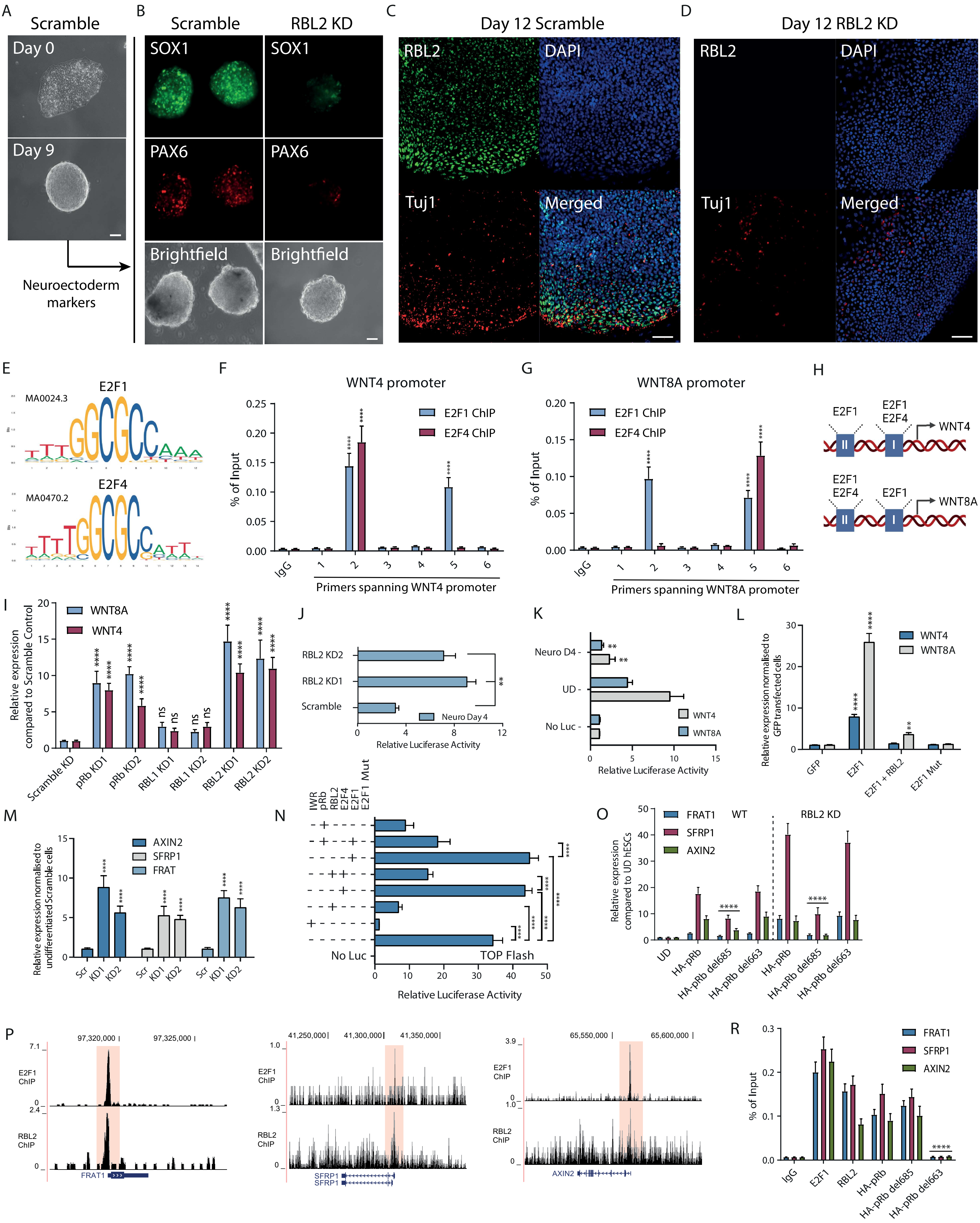
A
Scramble
Day 0
Day 9
Neuroectoderm markers
B
Scramble
RBL2 KD
SOX1
PAX6
Brightfield
C
Day 12 Scramble
RBL2
DAPI
Tuj1
Merged
D
Day 12 RBL2 KD
E
E2F1
MA0024.3
E2F4
MA0470.2
F
WNT4 promoter
% of Input
E2F1 ChIP
E2F4 ChIP
IgG
Primers spanning WNT4 promoter
G
WNT8A promoter
Primers spanning WNT8A promoter
H
E2F1
E2F4
WNT4
WNT8A
I
WNT8A
WNT4
Relative expression compared to Scramble Control
Scramble KD
pRb KD1
pRb KD2
RBL1 KD1
RBL1 KD2
RBL2 KD1
RBL2 KD2
J
RBL2 KD2
RBL2 KD1
Scramble
Neuro Day 4
Relative Luciferase Activity
K
Neuro D4 -
UD -
No Luc -
WNT4
WNT8A
Relative Luciferase Activity
L
WNT4
WNT8A
Relative expression normalised to GFP transfected cells
GFP
E2F1
E2F1 + RBL2
E2F1 Mut
M
AXIN2
SFRP1
FRAT
Relative expression normalised to undifferentiated Scramble cells
Scr
KD1
KD2
N
IWR
pRb
RBL2
E2F4
E2F1
E2F1 Mut
No Luc
TOP Flash
Relative Luciferase Activity
O
FRAT1
SFRP1
AXIN2
WT
RBL2 KD
Relative expression compared to UD hESCs
UD
HA-pRb
HA-pRb del685
HA-pRb del663
P
E2F1 ChIP
RBL2 ChIP
FRAT1
SFRP1
AXIN2
R
% of Input
IgG
E2F1
RBL2
HA-pRb
HA-pRb del685
HA-pRb del663

Figure S4: RBL2 represses WNT ligands WNT4 and WNT8A through their proximal promoter regions, Related to Figure 3. (A) Representative images of pluripotent H9 hESCs at day 0 and neural organoids at day 9. Scale bar 50 µm. (B) Immunostaining of SOX1 and PAX6 proteins in neural organoids at day 9 shows reduced expression of SOX1 and PAX6 in RBL2 KD cells. Scale bar 50 µm. (C) RBL2 expression is elevated in Tuj1 expressing cells in day 12 organoids. Scale bar 50 µm. (D) RBL2 KD causes spatial effects on Tuj1 expressing cell patterning of day 12 organoids compared to control Scramble cells (C). Scale bar 50 µm. (E) The similarity of E2F1 and E2F4 binding motifs. (F-G) E2F1 and E2F4 binding to two sites on WNT4 and WNT8A loci. (H) Schematic model of E2F1 and E2F4 competitive binding on WNT4 and WNT8A loci. (I) Comparison of pRb, RBL1 and RBL2 knockdown effects on WNT8A and WNT4 expression. (J) β-catenin is transcriptionally more active in RBL2 KD cells. Scramble and RBL2 KD cells were transfected with a TOP Flash luciferase construct containing a β-catenin responsive element. Significant differences compared to day 4 neuroectoderm Scramble shRNA cells calculated by two-way ANOVA are marked. (K) Relative promoter activity of WNT4 and WNT8A in HESCs and day 4 neuroectoderm cells. Luciferase assay of promoter constructs. Significant differences compared to undifferentiated (UD) HESCs calculated by two-way ANOVA are marked. (L) E2F1 induced endogenous WNT4 and WNT8A expression while RBL2 represses this effect. Cells transfected with E2F1, RBL2 and E2F1 mutant constructs were analysed 48 hours after transfection by Q-PCR. Significant differences compared to GFP transfected cells calculated by two-way ANOVA are marked. (M) RBL2 KD results in upregulation of known target genes of β-catenin. Gene expression was analysed by Q-PCR. Significant differences compared to day 4 neuroectoderm Scramble shRNA cells calculated by two-way ANOVA are marked. (N) RBL2 regulates the activity of β-catenin. RBL2 KD cells were cotransfected with a TOP Flash luciferase construct containing a β-catenin responsive element and a combination of expression constructs for RBL2 and E2F proteins. (O) Effects of pRb domain mutants on the expression of WNT pathway components FRAT1, SFRP1 and AXIN2. (P) Genomic views of E2F1 and RBL2 binding on FRAT1, SFRP1 and AXIN2. (R) The binding of E2F1, RBL2 and pRb mutants to FRAT1, SFRP1 and AXIN2 loci. All data are shown as mean+/-s.d. (n=3 biological replicates). Statistical analysis was performed by 2-way ANOVA with multiple comparisons with Tukey correction and **** marks adjusted P-value <0.0001, *** is adjusted P-value <0.001, ** is adjusted P-value <0.01, * is adjusted P-value <0.05.

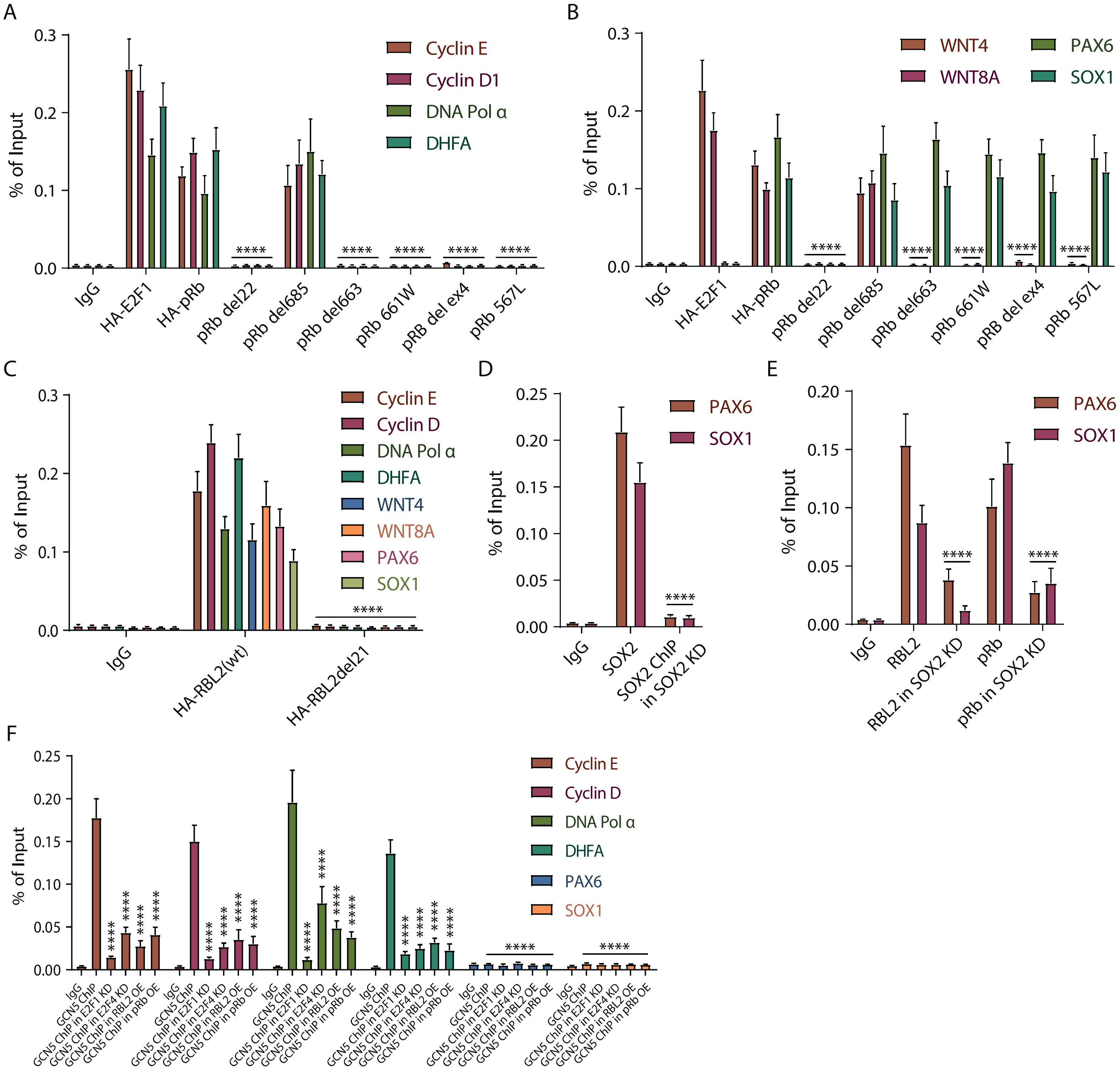

Figure S5: Characterising the regulation of RB target genes involved in cell cycle regulation and neuroectoderm differentiation, Related to Figure 3. (A) The binding of E2F1 and pRb mutants to cell cycle regulatory loci Cyclin E, Cyclin D1, DNA Pol α and DHFA loci. (B) The binding of E2F1 and pRb mutants to WNT ligand loci WNT4, WNT8A, and neuroectoderm differentiation genes PAX6 and SOX1. (C) RBL2 binds to cell cycle regulatory loci, WNT4, WN8A and neuroectoderm genes PAX6 and SOX1. (D) SOX2 binds to PAX6 and SOX1 loci during neuroectoderm differentiation at day 4. (E) SOX2 recruits RBL2 to PAX6 and SOX1. (F) GCN5 competes with RBL2 and pRb to bind to cell cycle regulatory loci via E2F1/4. All data are shown as mean+/-s.d. (n=3 biological replicates). Statistical analysis was performed by 2-way ANOVA with multiple comparisons with Tukey correction and **** marks adjusted P-value <0.0001, *** is adjusted P-value <0.001, ** is adjusted P-value <0.01, * is adjusted P-value <0.05.

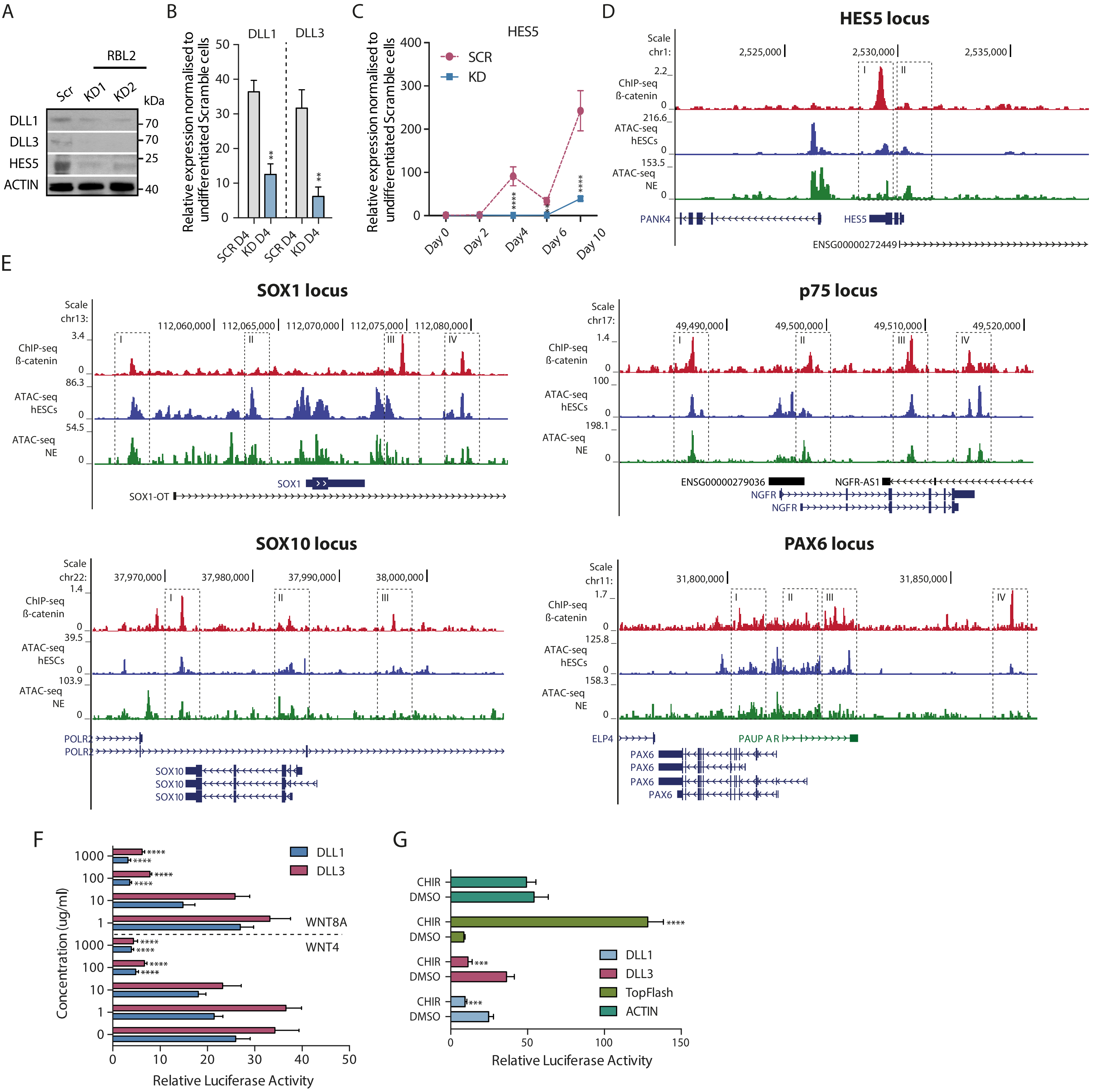

Figure S6: RBL2 regulates cell fate specification between neuroepithelium and neural crest through WNT and NOTCH ligands, Related to Figure 4. (A) Expression of NOTCH ligands is decreased in the absence of RBL2. Western blot analysis of NOTCH ligands and NOTCH target gene HES5 in Scramble and RBL2 KD cells. (B-C) RBL2 KD reduces the expression of NOTCH ligands DLL1, DLL3 and their target gene HES5 during neuroectoderm specification. Q-PCR analysis of NOTCH pathway members (B) in day 4 neuroectoderm or (C) during the time course of neuroectoderm differentiation. Significant differences compared to Scramble shRNA cells calculated by two-way ANOVA are marked. (D-E) Genomic regions of DLL1 and DLL3 loci. Genomic region of HES5 locus (D) and SOX1, p75, SOX10 and PAX6 locus (E) showing β-catenin ChIP-seq binding data in a cancer cell line together with hESC and neuroectoderm differentiating cells analysed by ATAC-seq. Genomic regions showing β-catenin binding peaks are highlighted with numbered dashed boxes. (F) WNT/β-catenin signalling reduces the promoter activity of DLL1 and DLL3 genes. Cells transfected with NOTCH promoter constructs were treated with a range of WNT4/WNT8A concentrations for 24h. Significant differences compared to DMSO treated samples calculated by two-way ANOVA are marked. (G) β-catenin activation by GSK3 inhibitor CHIR reduces the promoter activities of NOTCH genes. Cells were treated with CHIR for 24h. All data are shown as mean+/-s.d. (n=3 biological replicates). Statistical analysis was performed by 2-way ANOVA with multiple comparisons with Tukey correction and **** marks adjusted P-value <0.0001, *** is adjusted P-value <0.001, ** is adjusted P-value <0.01, * is adjusted P-value <0.05.

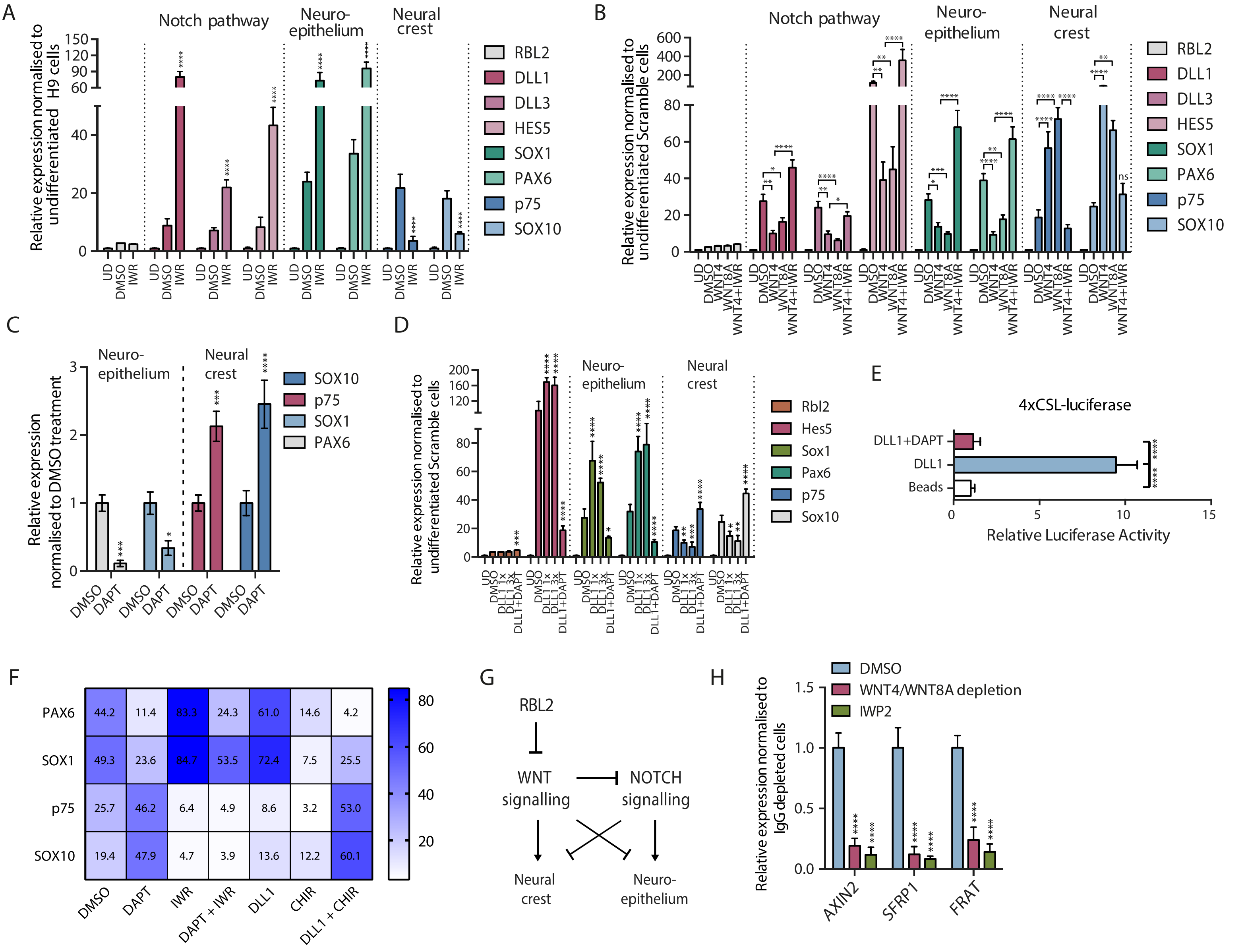

Figure S7: NOTCH signalling is indirectly regulated by RBL2 and mediates its effects on cell fate decisions in neuroectoderm, Related to Figure 4. (A) WNT inhibition reduces neural crest specification while increasing neuroepithelial fate and NOTCH pathway members. Day 3 neuroectoderm cells were treated for the days with WNT inhibitor and analysed by Q-PCR for NOTCH ligands, neuroepithelial markers and neural crest markers. Significant differences compared control DMSO treated cells calculated by two-way ANOVA are marked. (B) Purified WNT ligands WNT4 and WNT8A shift cellular specification toward neural crest fate while blocking NOTCH ligand expression. Differentiation marker analysis by Q-PCR after treatment of day 3 neuroectoderm cells with purified WNT4 and WNT8A for 3 days. (C) Inhibition of NOTCH signalling shifts the expression of developmental genes from neuroepithelial markers to neural crest markers. Q-PCR analysis of differentiation marker expression in day 4 neuroectoderm cells after NOTCH inhibition for 24h with 10μM DAPT. Significant differences compared to DMSO treated samples calculated by two-way ANOVA are marked. (D) Purified agarose-bound NOTCH ligand DLL1 shifts specification toward neuroepithelial cell fate. Differentiation marker analysis after treatment of cells with purified DLL1 for 24 hours. (E) Confirmation of NOTCH signalling activation by agarose-bound DLL1. Cells were transfected with a NOTCH responsive 4xCSL-luciferase construct were treated with agarose-bound DLL1 alone or with 10μM DAPT for 24h. Significant differences compared to control agarose beads calculated by two-way ANOVA are marked. (F) WNT and NOTCH cooperate to regulate the cell fate choice between neuroepithelium and neural crest. Cells were differentiated to day 3 neuroectoderm and then treated with WNT/NOTCH activators and inhibitors for 3 days followed by Q-PCR analysis. Significant differences compared to DMSO treated samples calculated by two-way ANOVA are marked. (G) Schematic overview of NOTCH signalling in regulating cell fate decisions. (H) Confirmation of WNT4/WNT8A depletion on the expression of known β-catenin target genes. Cells were incubated for 24h with media depleted from WNT4/WNT8A proteins or conditioned with WNT inhibitor IWP2 and analysed by Q-PCR. All data are shown as mean+/-s.d. (n=3 biological replicates). Statistical analysis was performed by 2-way ANOVA with multiple comparisons with Tukey correction and **** marks adjusted P-value <0.0001, *** is adjusted P-value <0.001, ** is adjusted P-value <0.01, * is adjusted P-value <0.05.
